# Antarctic blackfin icefish genome reveals adaptations to extreme environments

**DOI:** 10.1038/s41559-019-0812-7

**Published:** 2019-02-25

**Authors:** Bo-Mi Kim, Angel Amores, Seunghyun Kang, Do-Hwan Ahn, Jin-Hyoung Kim, Il-Chan Kim, Jun Hyuck Lee, Sung Gu Lee, Hyoungseok Lee, Jungeun Lee, Han-Woo Kim, Thomas Desvignes, Peter Batzel, Jason Sydes, Tom Titus, Catherine A. Wilson, Julian M. Catchen, Wesley C. Warren, Manfred Schartl, H. William Detrich, John H. Postlethwait, Hyun Park

**Affiliations:** 1 Unit of Polar Genomics, Korea Polar Research Institute, Incheon, Korea.; 2 Institute of Neuroscience, University of Oregon, Eugene, OR, USA.; 3 Department of Polar Life Science, Korea Polar Research Institute, Incheon, Korea.; 4 Polar Science, University of Science and Technology, Daejeon, Korea.; 5 Department of Animal Biology, University of Illinois, Champaign, IL, USA.; 6 McDonnell Genome Institute, Washington University, St. Louis, MO, USA.; 7 Department of Developmental Biochemistry, Biocenter, University of Wuerzburg, Wuerzburg, Germany.; 8 Hagler Institute for Advanced Study, Texas A&M University, College Station, TX, USA.; 9 Department of Biology, Texas A&M University, College Station, TX, USA.; 10 Department of Marine and Environmental Sciences, Northeastern University Marine Science Center, Nahant, MA, USA.

## Abstract

Icefishes (suborder Notothenioidei; family Channichthyidae) are the only vertebrates that lack functional haemoglobin genes and red blood cells. Here, we report a high-quality genome assembly and linkage map for the Antarctic blackfin icefish *Chaenocephalus aceratus*, highlighting evolved genomic features for its unique physiology. Phylogenomic analysis revealed that Antarctic fish of the teleost suborder Notothenioidei, including icefishes, diverged from the stickleback lineage about 77 million years ago and subsequently evolved cold-adapted phenotypes as the Southern Ocean cooled to sub-zero temperatures. Our results show that genes involved in protection from ice damage, including genes encoding antifreeze glycoprotein and zona pellucida proteins, are highly expanded in the icefish genome. Furthermore, genes that encode enzymes that help to control cellular redox state, including members of the *sod3* and *nqo1* gene families, are expanded, probably as evolutionary adaptations to the relatively high concentration of oxygen dissolved in cold Antarctic waters. In contrast, some crucial regulators of circadian homeostasis (*cry* and *per* genes) are absent from the icefish genome, suggesting compromised control of biological rhythms in the polar light environment. The availability of the icefish genome sequence will accelerate our understanding of adaptation to extreme Antarctic environments.

Antarctic icefishes inhabit the Earth’s coldest marine environment. These remarkable animals are the only vertebrates that lack functional red blood cells and functional haemoglobin genes; they are ‘white blooded’^1,2^. Icefish blood carries oxygen solely in physical solution, resulting in an oxygen-carrying capacity per unit of blood volume of less than 10% of that in closely related red-blooded Antarctic notothenioid fishes^1,3^. Blackfin icefish (*Chaenocephalus aceratus*), along with 5 other species among the 16 recognized species of icefishes (Channichthyidae) within the notothenioid teleosts, also lack cardiac myoglobin^4–6^. Icefishes evolved mechanisms that appear to compensate for loss of these oxygen-binding proteins, including enormous hearts with increased stroke volume relative to body size^7^, enhanced vascular systems^4^, and changes in mitochondrial density and morphology^4^.

Ancestral notothenioids were red-blooded but had no myoglobin in their skeletal muscle; they lived on the ocean floor and lacked a buoyancy-generating swim bladder. As Antarctica cooled, finally reaching −1.9 °C in the high Antarctic about 10–14 million years ago (Ma)^8^, ecological niches opened into which notothenioids radiated due to adaptive changes for cold tolerance^9^, including antifreeze glycoproteins (AFGPs) in larvae and adults^10,11^, and ice-resistant egg chorion proteins surrounding embryos^12^. Notothenioids, living in constant cold, evolved a substantially non-conventional heat-shock response^13,14^. From these benthic ancestors, eight notothenioid taxa, including the icefishes, evolved to exploit the food-rich water column through increased buoyancy, which was achieved by reducing densely mineralized elements such as bones and scales^15–17^ and increasing deposits of lipids^18^. Some icefishes became ambush feeders^9,19^ concomitant with craniofacial adaptations, and possibly a decrease in metabolic oxygen demand^7,17,20^. To help investigate the genomic basis for these extreme evolutionary adaptations, we sequenced the genome of the blackfin icefish.

## Results

### Genome assembly and annotation.

The genome of a female Antarctic blackfin icefish from the Antarctic Peninsula (Supplementary Figs. 1 and 2) was sequenced by single-molecule real-time technology with a PacBio Sequel instrument, yielding ~90× genome coverage and a 13-kilobase average read length (Supplementary Table 1). The genome size was estimated, by *k*-mer analysis using Jellyfish software, to be 1.1 gigabase pairs (Supplementary Fig. 3). The FALCON-Unzip assembled genome contained 3,852 contigs totalling 1.06 gigabase pairs with a contig N50 size of 1.5 megabase pairs (Mb) (Table 1). Evaluation of the genome for completeness based on BUSCO^21^ identified 89.9% complete and 3.6% fragmented genes from the 4,584gene Actinopterygii dataset (Supplementary Table 2). The icefish genome contains 30,773 inferred protein-coding genes based on combined ab initio gene prediction, homology searching and transcript mapping (Table 1 and Supplementary Tables 3 and 4). Small-RNA transcriptomics from 5 tissues facilitated annotation of microRNAs (miRNAs), identifying 290 miRNA genes that produced 334 unique mature miRNAs (Table 1 and Supplementary Tables 5 and 6). The icefish genome contains 50.4% repetitive sequences, most of which (47.4% of the total genome) are transposable elements (Supplementary Table 7 and Supplementary Note 1). Inferring the history of repeat elements by calculating the relative age of transposable element copies through Kimura distance analyses and comparisons with other teleosts (Supplementary Note 1) revealed a recent burst of DNA transposons and long and short interspersed elements. This result is consistent with the hypothesis^22^ that exposure to strong environmental changes, such as cooling to sub-zero temperatures and a series of glaciation and deglaciation cycles, led to massive mobilization of transposable elements.

### Genetic linkage map and genome assembly integration.

To make a chromonome (a chromosome length genome assembly)^23^, we constructed a genetic map for blackfin icefish. RAD-tag sequencing^24^ produced 20 million reads each for male and female parents, and an average of 2.4 million reads for each of 83 individual progeny. Stacks software^25^ identified 60,038 RAD-tags, of which 56,256 (93.7%) were present in at least 10 progeny. Of 7,215 polymorphic RAD-tags, 4,952 (55.8%) were present in at least 60 of 83 progeny and 4,023 localized to the male map, the female map or both at a minimum logarithm of the odds (LOD) of 12. JoinMap 4.1 assigned markers to 24 linkage groups (Supplementary Fig. 4, accession SRP118539)—one for each cytogenetic chromosome^26^. Because the map showed that each icefish chromosome was an orthologue of each medaka chromosome, we numbered icefish linkage groups to match their medaka counterparts. *C. aceratus* linkage group 6 (Cac6) had the most markers (202) and Cac2 had the fewest (131). Cac21 was the longest (65.8 cM) and Cac12 was the shortest (46.7 cM). Chromonomer software (http://catchenlab.life.illinois.edu/chromonomer) aligned contigs to the genetic map. Of 3,852 contigs in the assembly, 1,063 (27%) aligned on the genetic map by at least one marker for a total length of 820 Mb of the 1,065 Mb (77%) assembly. Only one contig was chimeric (Ice_000013): one end mapped to Cac7 and the other to Cac14 (Supplementary Fig. 4).

**Fig. 1 |:**
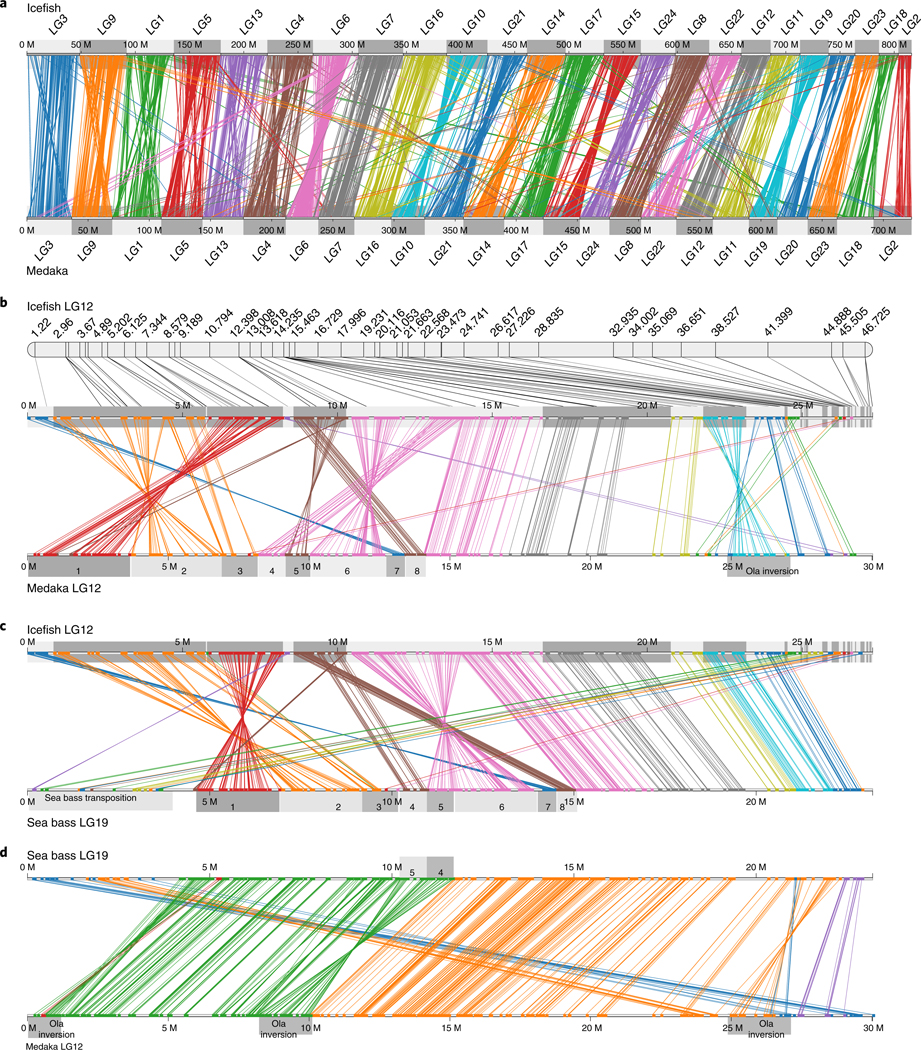
Chromosome stability of blackfin icefish with respect to teleost outgroups. **a**, Gene content in icefish chromosomes supports a one-to-one correspondence between icefish and medaka chromosomes. Each line represents orthologous genes in icefish and medaka, colour-coded by icefish chromosome. The few lines that cross linkage groups (LGs) probably represent paralogues. **b**, A comparison of orthologous gene orders in icefish LG12 (Cac12) and medaka LG12 (Ola12) illustrates icefish-specific chromosome inversions and transpositions (see text). Each line represents orthologous genes in the icefish and medaka chromosome, colour-coded by icefish genomic scaffold. Conserved syntenic blocks are labelled 1–8. **c**, Comparison of orthologous gene order in Cac12 and European sea bass LG19 (Dla19). Conserved syntenic blocks are labelled 1–8. **d**, Comparison of orthologous gene order between sea bass Dla19 and medaka Ola12 reveals that most chromosome rearrangements occurred after the divergence of the icefish lineage from the sea bass lineage. M, megabase position along the chromosome.

Synolog^27^ displayed conserved syntenies, revealing that each icefish chromosome is orthologous to a single chromosome in both medaka (*Oryzias latipes*, Ola; Fig. 1a) and European sea bass (*Dicentrarchus labrax*, Dla; Supplementary Fig. 5a). We detected a single small internal translocation (Supplementary Fig. 5d,e), but no reciprocal chromosomal translocations were found in the lineages of icefishes, sea bass and medaka since their lineages diverged ~113 Ma^28^. Chromosome stability in teleost fish is remarkable compared with mammals, where, for example, different deer species have between 3 and 40 haploid chromosomes and different rodents have between 5 and 51 (ref. ^29^). Although the blackfin icefish retains the ancestral chromosome number, many Antarctic notothenioids do not; for example, different species in the genus *Notothenia* have 13, 12 or 11 chromosomes rather than the ancestral 24 due to centromeric fusions of entire ancestral chromosomes^30^.

**Fig. 2 |:**
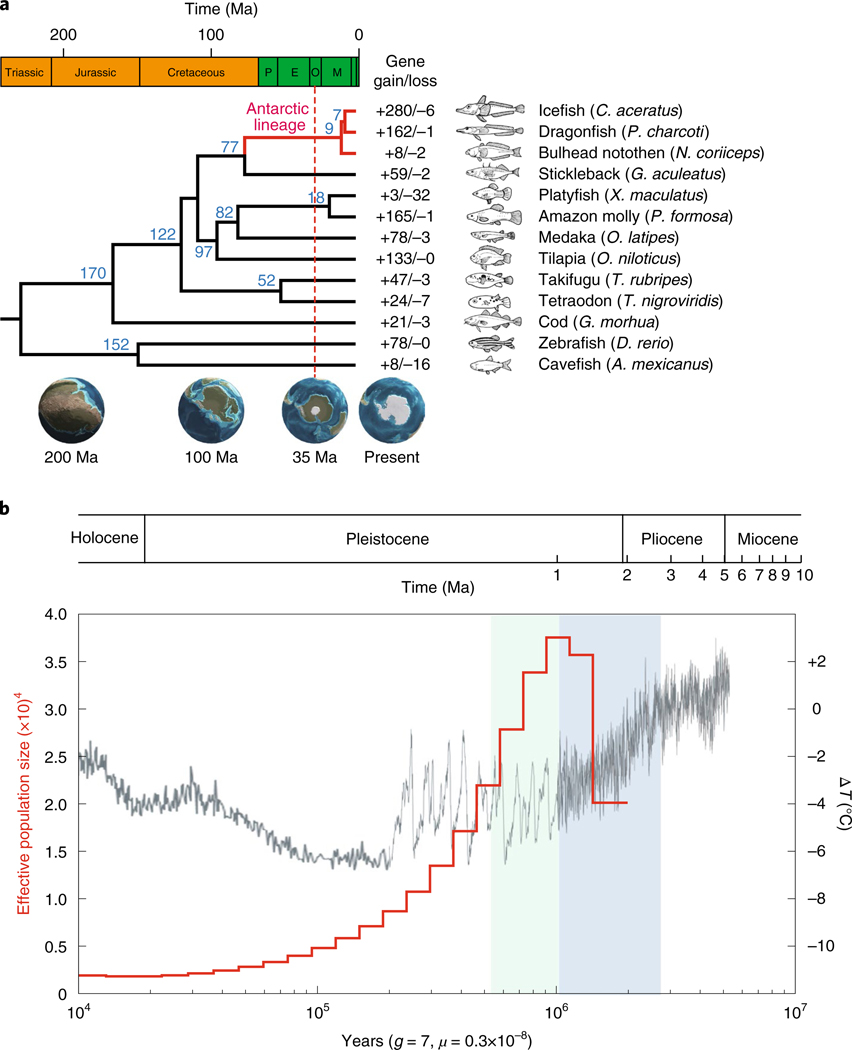
Comparative analysis of the *C. aceratus* genome assembly. **a**, Phylogenetic tree and gene family gain-and-loss analysis, including the number of gained gene families (+) and lost gene families (−). Blue numbers specify divergence times between lineages. The red dotted line indicates the appearance of Antarctic ice sheets (35 Ma), which allowed the circum-Antarctic current to form after the opening of the Drake Passage. Subsequent cooling of the Southern Ocean drove local extinction of most fish taxa and adaptive radiation of the Antarctic notothenioid suborder. E, Eocene; M, Miocene; O, Oligocene; P, Palaeocene. **b**, Inferring icefish population history by PSMC analysis. The left *y* axis represents the demographic history of *C. aceratus* (red line). During the Plio-Pleistocene (3–0.9 Ma), which is shaded blue, Antarctic sea-surface temperatures dropped by around 2.5 °C, judged by a proxy for marine palaeo-temperature changes based on oxygen isotope ratios^91,92^ (right *y* axis). Concomitant decreases in marine temperatures (black line) probably allowed the cold-adapted *C. aceratus* populations to increase in size. The green shading represents the mid-Pleistocene transition, during which temperature fluctuations were large. g, generation time; μ, mutation rate.

Although orthologous chromosomes in icefish, sea bass and medaka chromosomes share gene content, gene order was often not well conserved. For example, Cac12 and Ola12 contain multiple conserved syntenic blocks (Fig. 1b, blocks 1–8) rearranged by inversions and transpositions. Most of those blocks have the same order in Ola12 and Dla12, showing that rearrangements occurred in the icefish lineage after it separated from the sea bass lineage. Conserved blocks ‘4’ and ‘5’ appear in the opposite order in sea bass and stickleback chromosomes (Fig. 1c and Supplementary Fig. 5b), and comparisons between sea bass and medaka (Fig. 1d) or stickleback (Supplementary Fig. 5c) suggest an inversion in the medaka lineage. Other lineage-specific rearrangements in Cac12 are evident, and analysis of other chromosomes confirms that, despite the paucity of translocations over more than 100 Myr (Fig. 2a), multiple rearrangements within chromosomes occurred in the icefish lineage after it separated from the sea bass lineage (Supplementary Fig. 6).

### Phylogenomics and genome expansion.

A comparison of genome sequences by OrthoMCL showed that the blackfin icefish has 18,636 of 24,159 orthologous gene clusters identified in 13 teleosts. A genome-wide set of 3,718 one-to-one orthologues provided a phylogenetic tree of 13 teleosts using maximum likelihood (Supplementary Tables 8 and 9). According to the time-calibrated phylogeny, the common ancestor of the three Antarctic fishes with genome sequences (*C. aceratus, Parachaenichthys charcoti* (Charcot’s dragonfish) and *Notothenia coriiceps* (bullhead notothen)) diverged from the stickleback lineage ~77 Ma, and icefishes diverged from the dragonfish lineage ~7 Ma (Fig. 2a and Supplementary Fig. 7). Gene family analysis identified a core set of 9,647 gene families that were shared among 6 represented fishes (three Antarctic species, stickleback, medaka and zebrafish) and 445 blackfin icefish-specific gene families (Supplementary Fig. 8).

The icefish has 373 significantly expanded and 346 significantly contracted gene families based on the *z* score of gene count differences among 13 teleosts (Supplementary Tables 10 and 11). The blackfin icefish lineage experienced the largest gene family turnover among the 13 species after it diverged from the dragonfish (significant gains: 280 genes; significant losses: 6 genes) (Fig. 2a and Supplementary Table 12). Gene families with a significant number of genes gained were enriched for sensory perception (Supplementary Note 2), oxidoreductase activity and ion binding (Supplementary Table 13). Forty genes appeared to be positively selected specifically in the icefish lineage after divergence from the dragonfish lineage (Supplementary Table 14). Positively selected genes were enriched in two functional categories: oxidoreductase activity (presumably related to life without haemoglobin) and lipid binding (presumably related to buoyancy increase connected to changes from a strictly benthic lifestyle) (Supplementary Table 15).

### Genomic variation and population history.

We identified 9,365,677 heterozygous single nucleotide polymorphisms in the genome of the sequenced icefish female, resulting in a frequency of heterozygous sites in the sequenced fish of 8.79 × 10^−3^, which is greater than other individual genomes of marine fish such as the Atlantic cod (2.09 × 10^−3^)^31^ and ocean sunfish (0.78 × 10^−3^)^32^. Analysis using the pairwise sequentially Markovian coalescent (PSMC) model^33^ suggested two epochs that shaped icefish demographic history. First, icefish populations appeared to reach maximum size ~1 Ma at the end of the Plio-Pleistocene cooling event (3.0–0.9 Ma), after Antarctic ocean surface temperatures had dropped by 2.5 °C^34^. Adaptations made during the slow cooling of the Plio-Pleistocene may have allowed the icefish lineage time to achieve its maximum effective population size. Second, icefish populations appeared to decline during temperature fluctuations in the mid-Pleistocene transition (~1.2–0.55 Ma)^35^ (Fig. 2b), which probably presented a physiological burden for the thermally sensitive icefish^35^.

### Expansion of AFGP and zona pellucida gene families.

**Fig. 3 |:**
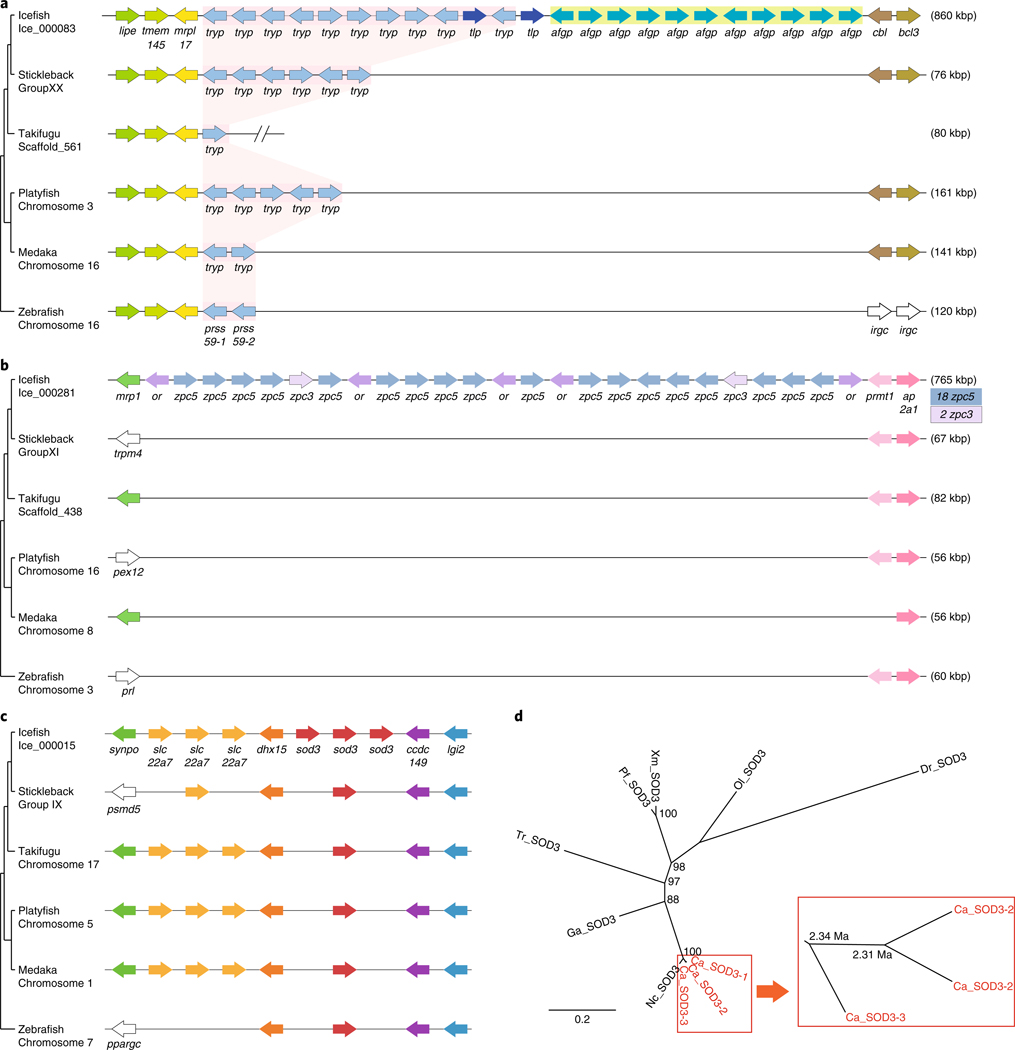
Conserved syntenies for expanded gene clusters identified in the blackfin icefish genome. **a**, AFGP and trypsinogen gene loci. The pink-shaded area indicates the trypsinogen gene locus. kbp, kilobase pair. **b**, Zona pellucida c5 (*zpc5*) locus. **c**, *sod3* gene cluster. Genomic neighbourhoods are shown within representative sequenced teleost genomes. Each arrow indicates a complete gene orientated in the (5′ → 3′) direction. **d**. Phylogenetic analysis of vertebrate *sod3* genes. Divergence times were calculated by applying the mutation rate formula *μ* = *D*/2*t* = 3.28 × 10−^9^. Ca, *C. aceratus* (icefish); Dr, *D. rerio* (zebrafish); Ga, *G. aculeatus* (stickleback); Nc, *N. coriiceps* (bullhead notothen); Ol, *O. latipes* (medaka); Pf, *P. Formosa* (Amazon molly); Tr, *T. rubripes* (fugu); Xm, *X. maculatus* (platyfish).

AFGP genes, which evolved from trypsinogen genes^36^, were tandemly duplicated in icefish as they are in Antarctic toothfish (*Dissostichus mawsoni*)^37^ (Fig. 3a). Our results show that the Antarctic fish AFGP–trypsinogen locus is situated between mitochondrial ribosomal protein L (*mrpl*) and E3 ubiquitin-protein ligase CBL (*cbl*), consistent with the location of the trypsinogen gene in several percomorph teleosts (Fig. 3a). A low-coverage Illumina-based draft assembly of the *C. aceratus* genome annotated 4 copies of AFGP genes^38^, whereas our results revealed 11 copies of AFGP genes adjacent to 10 tandem copies of trypsinogen genes and 2 copies of trypsinogen-like protease genes.

Antarctic fish embryos do not appear to express AFGP genes^12,39^, which raises the question of how these embryos resist freezing^12^. Zona pellucida egg-coat proteins play roles in fertilization and preventing polyspermy in mammals^40^, and provide thickness and hardness to fish eggshells^41^. Zona pellucida proteins from Antarctic toothfish depress the melting point of ice^12^. The zona pellucida protein family expanded extensively in the *C. aceratus* genome: 131 zona pellucida genes, including 109 tandemly duplicated genes on 20 contigs (Supplementary Table 16), fell into 11 subfamilies based on sequence similarities (Supplementary Table 17). In contrast with our icefish sequence, 2 other Antarctic species—the bullhead notothen *N. coriiceps*^42^ and dragonfish *P. charcoti*^43^—had only 18 or 30 zona pellucida genes, respectively, and only 16–35 zona pellucida genes appeared in 7 non-Antarctic teleosts based on Ensembl queries. Furthermore, transcripts of only 18 zona pellucida genes were found in the Antarctic toothfish^12^. Blackfin icefish had many more copies of the *zpax1*, *zpc1*, *zpc2* and *zpc5* genes than other fish (Supplementary Table 16). For instance, 18 *zpc5* genes were tandemly duplicated in a single contig (Ice_000281) that also contained two *zpc3* paralogues (Fig. 3b). Another contig (Ice_000114) had five *zpc3* and three *zpc5* genes, which suggests conserved synteny of these paralogues, in agreement with their location in the genomes of stickleback and medaka (Supplementary Fig. 9). The ovaries and liver express zona pellucida genes in vertebrates^44^, and most *C. aceratus* zona pellucida genes were strongly expressed in the ovaries, similar to three other Antarctic fish^12^ (Supplementary Fig. 10), although transcription of several zona pellucida genes was detected in other *C. aceratus* organs. It is possible that the extraovarian expression observed for some zona pellucida paralogues represents adaptive, *C. aceratus*-specific neofunctionalization^45^ of this expanded gene family.

### Genes for oxygen-binding proteins.

Most teleost genomes have two globin gene clusters that arose during the teleost genome duplication: the LA cluster (with *lcmt1* and *aqp8* on one side and *rhbdf1b* on the other) and the MN cluster (flanked by *mpg* and *nprl3* on one side and *kank2* on the other)^46^. Within each teleost *hb* cluster, α- and β-chain genes generally alternate, in contrast with mammals, in which α- and β-gene clusters reside on different chromosomes^46^. The loss of *hbb* genes and pseudogenation of *hba* genes is an icefish synapomorphy; 15 of 16 icefish species retained only a 3′ fragment of an α-globin gene^2^. The sixteenth species retained an intact but unexpressed *hba* gene fused to two β-pseudogenes, which was probably inherited from ancestors due to incomplete lineage sorting^2^. The results show that the residual α-globin fragment in blackfin icefish mapped to the LA cluster, and the blackfin icefish genome possesses no trace of the MN cluster, although surrounding genes were preserved intact (Supplementary Fig. 11). In contrast, intact genes for myoglobin^47^, cytoglobin^48^ and neuroglobin^49^ appear in the *C. aceratus* genome in the context of conserved synteny among teleosts (Supplementary Fig. 12). Our sequence provides the substrate to learn the molecular genetic mechanisms that inhibit myoglobin expression, which remain to be elucidated.

### Oxidative stress.

Some icefishes, including *C. aceratus*, are more sensitive to oxidative stress than red-blooded notothenioids are^50–52^. The volume of polyunsaturated-fatty-acid-rich mitochondria per volume of skeletal or cardiac muscle cell in icefishes is approximately twice as large as in red-blooded Antarctic fishes, which may make icefishes more susceptible to reactive oxygen species (ROS) formation and lipid peroxidation at current environmental temperatures^52^. Furthermore, the lower thermal tolerance of icefishes relative to red-blooded notothenioids may be due to increased protein and lipid damage in icefish cardiac muscle^50^. If disrupted, the respiratory chain in icefish mitochondria generates more ROS than red-blooded Antarctic fish^51^. Finally, levels of antioxidants in icefishes are low relative to red-blooded notothenioids^52^. These data suggest that some icefishes are probably under selective pressure to enhance their antioxidant defence systems^53^.

Gene families associated with ROS homeostasis (Supplementary Table 18), including those encoding superoxide dismutase (SOD) and NAD(P)H:quinone acceptor oxidoreductase (NQO), were expanded in the *C. aceratus* genome. We found that blackfin icefish has five *sod* genes (*sod1*, *sod2* and three tandemly repeated copies of *sod3*) compared with just three *sod* genes typical for other percomorph teleosts (Fig. 3c and Supplementary Table 19). Mutation rate analysis suggested that the *sod3* gene duplicates arose as recently as ~2.3 Ma (Fig. 3d). Because the multiple *sod3* genes appear to encode extracellular SOD3 enzymes (each protein possesses an apparent secretory signal peptide), an understanding of their roles in extracellular versus intracellular ROS homeostasis will require further study.

The expansion of *nqo1* genes in the icefish genome was striking: we found a total of 33 genes, in contrast with the 2–10 *nqo1* genes annotated in most fish genomes (Supplementary Fig. 13). Teleosts generally have two loci containing *nqo1* genes in the genomic contexts *vang*-*nqo1s*-*ackr3* and *il17*-*nqo1*-*gabarapl*; both regions appear to have been ancestrally linked, as they are today in pufferfish (*Takifugu rubripes*) and medaka, and the icefish *nqo1* genes conformed to this pattern. However, *C. aceratus* possessed 26 additional *nqo* genes on 3 contigs that did not appear to be part of the 2 conserved *nqo1* loci (Supplementary Fig. 13). In addition, the icefish is the only sequenced teleost to have two tandem copies of 8-oxoguanine DNA glycosylase (*ogg1*), which encodes a protein that excises from DNA a modified base that arises from reactive oxygen damage, whereas other sequenced teleost genomes have just one *ogg1* copy (Supplementary Fig. 14).

### Circadian adaptation to extremely fluctuating photoperiods.

**Fig. 4 |:**
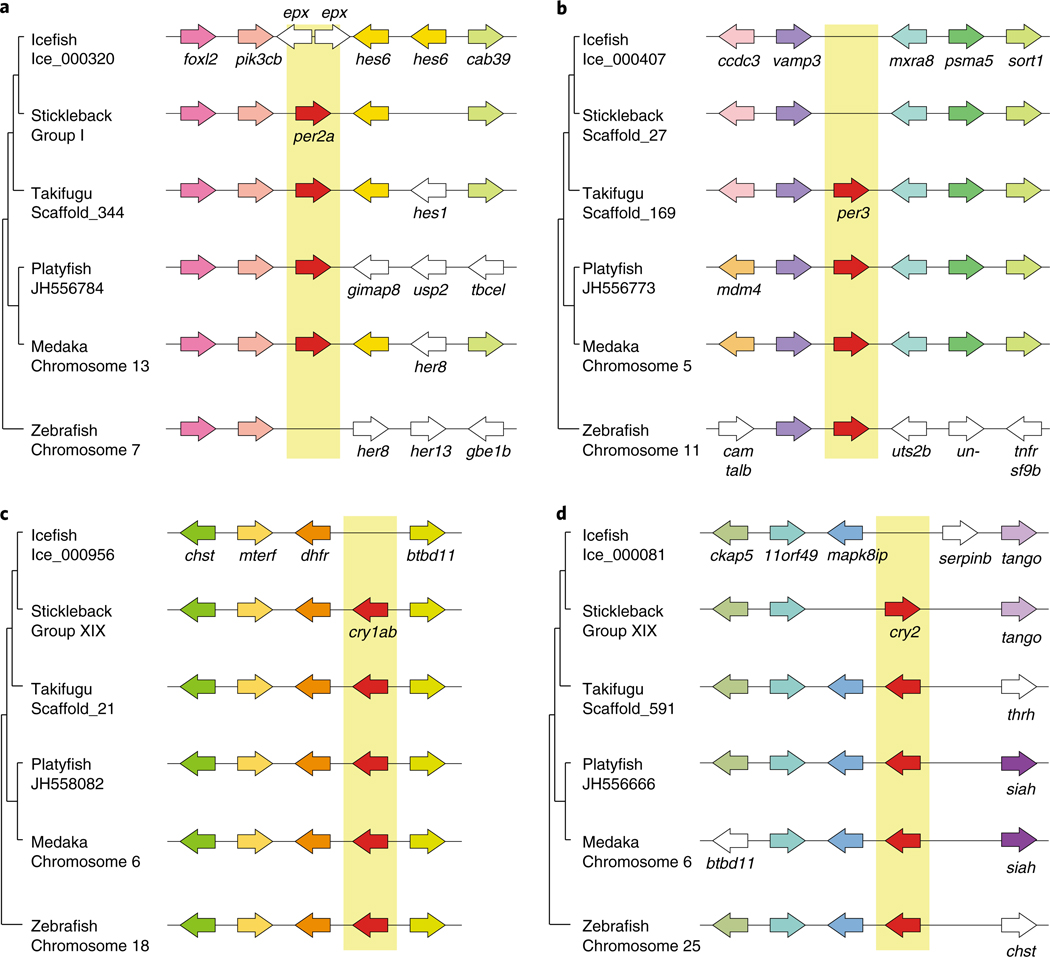
Genomic evidence supporting gene loss events for blackfin icefish circadian rhythm-related genes. **a**,**b**, Genomic structures and syntenic comparisons of the period genes *per2a* (**a**) and *per3* (**b**). **c**,**d**, Cryptochrome gene clusters for the *cry1ab* (**c**) and *cry2* (**d**) are shown within representative sequenced teleost genomes.

Polar species inhabit an environment with extreme annual fluctuations of day length, raising questions regarding the role of circadian rhythm genes in these organisms. The *cry* and *per* genes regulate a phylogenetically conserved circadian feedback loop by reciprocal transcriptional controls^54^. Teleost genomes have various numbers of *cry* genes (for example, seven in zebrafish and five in stickleback)^55^. Although icefish maintained the genomic structure around *cry* and *per* genes that is strongly conserved in teleost genomes, *cry1*, *cry2*, *per2a and per3* sequences appeared to be specifically deleted in icefish evolution (Fig. 4a and Supplementary Table 20). The icefish genome possesses only three *cry* genes—the smallest number identified in any teleost. Although the bullhead notothen and dragonfish genome assemblies are incomplete, they also possess and lack the same circadian rhythm genes as blackfin icefish; thus, available evidence promotes the hypothesis that the extremes of winter darkness and summer light may have reduced the utility of, and hence decreased the pressure to retain, some circadian rhythm regulators in Antarctic fish. Behavioural studies on Antarctic icefishes and other notothenioid species will be necessary to validate this hypothesis.

## Concluding remarks

Here, we report a high-quality genome assembly and linkage map, which together provide a chromonome for the Antarctic blackfin icefish. This assembly reveals the remarkable stability of teleost chromosome contents across at least 110 Myr, with all 24 chromosomes mutually orthologous in medaka, European sea bass and blackfin icefish. Comparative analysis of the icefish chromonome revealed more inversions and intrachromosomal transpositions when comparing icefish and European sea bass than between the more anciently diverged European sea bass and medaka. Whether this rate change accompanied the chilling of Antarctica or is restricted to the icefish radiation requires comparisons with equivalently high-quality genome sequences for other notothenioid fish. The icefish genome sequence places into a genomic context the expansion of genes in the AFGP/trypsinogen/trypsinogen-like protease locus, the expansion of presumably ice-protective zona pellucida-encoding genes, and the loss of active haemoglobin genes. Also, we provide evidence for the expansion of gene families involved in the cellular redox state, including *sod3* and *nqo1*, which might represent evolutionary adaptations to ROS production associated with mitochondrial expansion in this white-blooded clade. Uniquely, we find that some genetic regulators of circadian rhythms (*cry* and *per* paralogues) that are present in all or most other teleosts are missing from the icefish genome, fitting a hypothesis that extremes in polar day lengths altered circadian regulation in Antarctic fish. Finally, the blackfin icefish genome provides an elegant natural model to facilitate exploration of genomic contributions to a wide range of evolutionary, ecological, metabolic, developmental and biochemical features of Antarctic fish as they adapted to the extreme low temperatures, high oxygen levels and greatly fluctuating day lengths of Antarctica.

## Methods

### Sample collection for genome sequencing.

Antarctic blackfin icefish *C. aceratus* (length: ~30 cm) (Supplementary Fig. 1) were collected from depths of 20–30 m in Marian Cove, near King Sejong Station, on the northern Antarctic Peninsula (62° 14′ S, 58° 47′ W) (Supplementary Fig. 2) in January 2017 using a baited hook and line. Local water temperatures averaged 1.6 ± 0.8 °C. Genomic DNA was isolated from a single female specimen. Additional *C. aceratus* were collected for construction of a genetic linkage map, and for miRNA sequencing in May of 2012–2015 by bottom trawling from the Antarctic Research and Supply Vessel *Laurence M. Gould* at depths of 160–200 m southwest of Low Island (Antarctic Specially Protected Area 152, Western Bransfield Strait; latitudes 63° 15′ S–63° 30′ S; longitudes 62° 00′ W–62° 45′ W, bounded on the northeast by Low Island)). These fish were transported alive to Palmer Station, Antarctica, where they were maintained in seawater aquaria at −1–0 °C following best practices for maintaining icefishes in captivity^56^.

### Genome sequencing and de novo assembly.

Genomic DNA of a single female icefish (Supplementary Fig. 1) was extracted from muscle tissue. Genomic DNA libraries were prepared according to the manufacturer’s instructions, and the libraries were sequenced using a PacBio Sequel System using P6-C4 sequencing chemistry (DNA Link). The average coverage of single-molecule real-time sequences was ~90-fold and the average subread length was 10.6 kilobases from genomic DNA libraries. The FALCON-Unzip assembler provided a de novo assembly^57^. We used BUSCO to evaluate genome completion using the 4,584-Actinopterygii gene dataset^21^. For genome size estimation from DNA libraries, *k*-mer analysis was performed using Jellyfish^58^ (Supplementary Table 3).

### Organ-specific RNA sequencing.

The RNeasy Mini Kit (Qiagen) was used, according to the manufacturer’s instructions, to extract total RNA from 12 tissues (the brain, eye, gill, heart, intestine, kidney, liver, muscle, ovary, skin, spleen and stomach) from the individual used for genome sequencing (Supplementary Table 3). The quality of the total RNA was evaluated using an Agilent Bioanalyzer (integrity values ≥ 8). Transcriptome library construction and sequencing were performed using an Illumina HiSeq 2500 system. Illumina paired-end reads (2 × 100) from each organ were mapped to icefish genomic contigs using TopHat^59^, and the transcriptome of each tissue was assembled using Cufflinks (http://coletrapnell-lab.github.io/cufflinks).

### Gene annotation.

We used MAKER to perform genome annotation^60^. We constructed a de novo repeat library using RepeatModeler (version 1.0.3)^61^, including the RECON and RepeatScout^62^ software with default parameters. Tandem Repeats Finder^63^ was used to predict consensus sequences, classifying information for each repeat and tandem repeat, including simple repeats, satellites and low-complexity repeats. Repetitive elements were identified using RepeatMasker^64^. Subsequently, the repeat-masked genomes with BLASTn and protein information from tBLASTx were used for ab initio gene prediction with SNAP software^65^. Reference proteins from teleosts with sequenced genomes (*Danio rerio, Gasterosteus aculeatus, Gadus morhua, Tetraodon nigroviridis, T. rubripes, Astyanax mexicanus, O. latipes, Poecilia formosa, Oreochromis niloticus* and *Xiphophorus maculatus*) and two sequenced Antarctic fishes (*P. charcoti* and *N. coriiceps*) were included in the comparative analyses with transcripts of *C. aceratus* (Supplementary Table 3). Exonerate software, which provides integrated information for SNAP annotation, was applied to polish MAKER alignments. Next, MAKER selected and revised final gene models considering all information. MAKER predicted a total of 30,773 icefish genes. To identify noncoding RNAs in icefish contigs, we used the Infernal software package (version 1.1)^66^ and covariance models from the Rfam database^67^. Putative transfer RNA genes were identified using tRNAscan-SE^68^, which uses a covariance model that scores candidates based on their sequence and predicted secondary structures.

### miRNA sequencing and annotation.

Organs for miRNA annotation originated from the same male specimen of *C. aceratus* used in the study of erythropoietic miRNAs in Antarctic icefishes^69^. Samples of pronephric (head) kidney, pectoral girdle bone, heart ventricle, pectoral adductor muscle and skeletal muscle were dissected and stored in RNAlater at −80 °C until further use at the University of Oregon. Procedures were performed according to protocols approved by the Institutional Animal Care and Use Committee (IACUC) of the University of Oregon (10–26) and the IACUC of Northeastern University (12–0306R).

Total RNAs were extracted using the Zymo Research Direct-zol RNA MiniPrep kit according to the manufacturer’s instructions. Five tissue-specific small RNA libraries were then prepared and barcoded using the Bioo Scientific NEXTflex Small RNA sequencing kit with 15 PCR cycles, and sequenced by Illumina HiSeq 2500 at the University of Oregon Genomics and Cell Characterization Core Facility. Raw single-end 50-nucleotide-long reads were deposited in the National Center for Biotechnology Information Short Read Archive under accession number SRP069031. Reads from the five libraries were processed together using the bioinformatic tool Prost! (https://github.com/uoregon-postlethwait/prost)^70^. Briefly, raw reads were trimmed from adaptor sequences, filtered for quality using the FASTX-Toolkit, bioinformatically filtered for length between 17 and 25 nucleotides, filtered for a minimum of 5 identical reads, and grouped by genomic location. Groups of sequences were then annotated against mature and hairpin sequences present in miRBase release 21 (ref. ^71^), the extended zebrafish miRNA annotation^72^, the spotted gar annotation^73^ and the three-spined stickleback annotation. Gene nomenclature follows recent conventions^74^, including those for zebrafish^75^.

### Construction of the *C. aceratus* genetic linkage map.

#### Animals and in vitro fertilization.

The *C. aceratus* linkage map was produced from Low Island specimens obtained in 2012. Eggs from a single female and sperm from a single male were stripped, gametes were mixed, and progeny from this single in vitro fertilization event were maintained at about −1 °C until embryos were 2 months old, when they were euthanized and stored in 100% EtOH at −20 °C. We euthanized male and female parents by MS-222 overdose, then collected samples of muscle, liver, fin and spleen, which we stored in 100% EtOH at −20 °C. From each individual embryo and each individual parent, we isolated genomic DNA using the DNeasy Blood & Tissue Kit (Qiagen). The University of Oregon IACUC approved all protocols (13–27RR).

#### Creation of RAD-tag libraries.

Genomic DNA was purified from the male and female parents and 91 of their progeny. RAD-tag libraries were created as described^24,30^. DNA from each embryo was digested with high-fidelity SbfI (New England Biolabs) restriction enzyme overnight and each sample was separately barcoded with P1 and P2 adaptor ligations overnight, using 91 different 5-nucleotide barcodes. Pooled, size-selected DNA (50 ng) was amplified by PCR for 12 cycles, and the PCR product was gel purified by excising a 200–500 base pair (bp) fraction. Libraries were sequenced on the University of Oregon’s Illumina HiSeq 2000 to obtain 100-nucleotide single-end reads.

#### Marker genotyping.

Sequenced reads from the Illumina runs were sorted by barcode, allowing up to one mismatched nucleotide in the barcode sequence. Reads containing uncalled bases and those that had an average Phred quality score that fell below 10 over 15% of the read length were discarded. Retained reads were genotyped using Stacks software version 1.30 (ref. ^25^). Progeny with fewer than 1,000,000 reads were excluded from Stacks analysis. The parameters for Stacks provided to denovo_map.pl were: a minimum of 20 reads for a stack (-m 20), up to 4 differences when merging stacks into loci (-M 4) and up to 2 fixed differences when merging loci from the parents into the catalogue (-n 2). Stacks exported data into JoinMap 4.1 (ref. ^76^) for linkage analysis.

#### Map construction.

Of the 91 F1 individual progeny sequenced, 8 fish had too little coverage or too many missing genotypes (>20%) to use for mapping. Linkage analysis was performed with JoinMap 4.1 (ref. ^76^), with markers present in at least 60 of the 83 remaining individuals. Markers were initially grouped in JoinMap 4.1 using the ‘independence LOD’ parameter under ‘population grouping’ with a minimum LOD value of 12.0. Markers that remained unlinked at LOD < 12 were excluded. Marker ordering was performed using the maximum-likelihood algorithm in JoinMap 4.1 with default parameters. Putative double recombinants were identified using the ‘genotype probabilities’ feature in JoinMap 4.1 and by visual inspection of the colourized graphical genotypes. After visual inspection of the individual sequences in Stacks, markers were corrected as needed. For example, if a double recombinant was a homozygote with a small number of reads, the genotype was eliminated because it might represent a heterozygote that by chance lacked sufficient depth to provide a sequence for the second allele. Likewise, if a double recombinant was a heterozygote with only one sequence for the second allele, the genotype was eliminated because the second sequence might represent a sequencing error. The new dataset with corrected genotypes was loaded again into JoinMap 4.1, and linkage analysis was repeated until JoinMap identified no suspicious genotypes. The ‘expected recombination count’ feature in JoinMap 4.1 was used to identify individuals with more recombination events than expected. Visual inspection of the marker order was performed, and when necessary, the marker order was manually optimized by moving a marker or group of markers to a new position that reduced the total number of recombination events.

#### Integration of the genome assembly and linkage map.

The nucleotide sequence of the 4,023 polymorphic markers localized on the genetic linkage map was used as a search query against the sequence of assembled icefish contigs using GSNAP^77^, requiring unique alignments, and allowing up to 10% mismatches (-m 0.1). Markers from the genetic linkage map were integrated with the contigs from the icefish genome assembly to create a chromosome level assembly (or chromonome) using the software Chromonomer (http://catchenlab.life.illinois.edu/chromonomer/) as described^27^.

#### Analysis of conserved syntenies.

We used J. Catchen’s software Synolog^27^ to visualize the location of orthologous and paralogous genes among the genomes of icefish, medaka, European sea bass and stickleback, and to identify regions of conserved synteny between the different species.

### Comparative genomics analyses.

We identified orthologous gene clusters using the OrthoMCL^78^ pipeline, which applies the Markov clustering algorithm. Default options were used in all steps for genome sequences from 13 species (Supplementary Table 8). Analysis utilized available well-annotated and well-assembled genomes of species selected to represent clades critical for the analyses. For icefish, we used coding sequences based on the MAKER annotation pipeline. The orthologous gene clusters were categorized from one-to-one to many-to-many. Single-copy genes and species-specific genes were considered as singletons. Phylogenetic tree construction was performed based on one-to-one, single-copy orthologous genes. The sequences of protein-coding genes were aligned using the Probabilistic Alignment Kit (PRANK)^79^ with the codon alignment option. Poorly aligned regions with gaps were removed using Gblocks^80^ with a codon model for subsequent procedures. The maximum-likelihood method was applied to construct a phylogenetic tree using RAxML with 1,000 bootstraps, and divergence times were calibrated with TimeTree (median estimates of the pairwise divergence time for *D. rerio* and *G. morhua*: 230.4 Ma)^81^ using MCMCtree implemented in PAML packages^82^. The likelihood analysis for gene gain and gene loss was identified using CAFÉ 4.0 (ref. ^83^) with *P* < 0.05. The separate birth (*λ*) and death (*μ*) rates were estimated using the lambdamu command implemented in CAFÉ 4.0, with –s and –t options.

### Positively selected genes.

We identified 6,715 orthologous groups shared by 13 teleosts. A total of 3,718 single-copy gene families were used to construct a phylogenetic tree and to estimate times since lineage divergence using the methods described above. Using PRANK, orthologous gene sequences were aligned, and poorly aligned sequences with gaps were removed using Gblocks^80^. Alignments showing less than 40% identity and genes shorter than 150 bp were eliminated in subsequent procedures. Using the Codeml programme, the values of *dN* (ratio of nonsynonymous), *dS* (ratio of synonymous) and *ω* (average *dN*/*dS* ratio) were estimated for each gene implemented in the PAML package with the free-ratio model^82^ under F3X4 codon frequencies. The orthologues with *ω* ≤ 5 and *dS* ≤ 3 were retained^84^. We applied basic and branch-site models to define positively selected genes, and likelihood ratio tests were used to remove genes under relaxation of selective pressure. To identify functional categories and pathways that were enriched in the positively selected genes, we performed the Blast2GO enrichment test^85^ with the Fisher’s exact test (cutoff: *P* ≤ 0.05).

### Inference of demographic history.

The PSMC analysis^33^, based on a hidden Markov model, has been used to estimate the history of effective population sizes based on genome-wide heterozygous sequence data^32,86^. To obtain consensus diploid sequences, error-corrected reads from the female icefish used for genome sequencing were aligned back to the reference genome using BWA-MEM (BWA version 0.7.15)^87^ with default settings. Heterozygous variants were called using SAMtools mpileup (version 1.2) with bcftools call (version 1.2) and vcfutils.pl vcf2fq pipeline (https://github.com/lh3/psmc) with the following settings: the excessive mismatch containing reads filtering (-C) was set to 50, the minimum read depth (-d) was set to 8 (which is one-third of the average genome coverage of 24) and the maximum read depth (-D) was set to 49 (which is twice the average genome coverage). Genome-wide consensus fastq files were converted to PSMC modelling input files (psmcfa) using the fq2psmcfa tool provided in the PSMC package. Inference of population history was carried out using the psmc tool with the options ‘-N25 -t15 -r5 -p “4 + 25*2 + 4 + 6’ and plotted setting the generation time to 7 (because *C. aceratus* first spawns eggs at an age of 6–8 years) and a mutation rate of 3.28 × 10^−9^ (which is the rate obtained in this study). The mutation rate was estimated based on the Jukes–Cantor distance (*D*) between *C. aceratus* and *P. charcoti* among 3,718 one-to-one orthologous genes using the mutation rate formula *μ* = *D*/2*t* = 3.28 × 10^−9^ (*D* = 0.011 and *t* = 6.7 Myr were obtained in this study).

### Gene family analyses.

Genes belonging to families of interest were curated using manual gene search methods. Target gene sequences from the genomes of several teleost fishes were used to directly search the icefish genome database. The protein sequences of these manually curated genes were aligned using Clustal Omega^88^ and MUSCLE^89^. Phylogenetic trees were constructed using FastTree^90^ or RAxML with 1,000 bootstraps. The timing of gene duplication events was estimated using the mutation rate formula *μ* = *D*/2*t* = 3.28 × 10^−9^.

### Reporting Summary.

Further information on research design is available in the Nature Research Reporting Summary linked to this article.

## Supplementary Material

Kim Icefish genome supplementary material

## Figures and Tables

**Table 1 |:** Icefish assembly and annotation statistics

**Assembly**	
Number of contigs	3,852
Total genome length from contigs (bp)	1,065,645,509
Longest contig (bp)	9,422,831
N50 contig length (bp)	1,500,626
**Annotation**	
Number of genes	30,773
Exon number	277,249
Total length of exons (bp)	50,279,998
Total length of repeats (bp)	523,290,133
G + C (%)	42.08
