# Supplementary material for: Antarctic blackfin icefish genome reveals adaptations to extreme environments
Source: Nat Ecol Evol. Author manuscript; Available in PMC 2020 Jun 22. (PMC7307600; doi:10.1038/s41559-019-0812-7)
Supplement: Kim Icefish genome supplementary material [file NIHMS1585566-supplement-Kim_Icefish_genome_supplementary_material.pdf]

nature ecology & evolution
SUPPLEMENTARY INFORMATION
https://doi.org/10.1038/s41559-019-0812-7

In the format provided by the authors and unedited.

Antarctic blackfin icefish genome reveals adaptations to extreme environments

Bo-Mi Kim[1], Angel Amores[2], Seunghyun Kang[1], Do-Hwan Ahn[1], Jin-Hyoung Kim[1], Il-Chan Kim[3], Jun Hyuck Lee[1,4], Sung Gu Lee[1,4], Hyoungseok Lee[1,4], Jungeun Lee[1,4], Han-Woo Kim[1,4], Thomas Desvignes[2], Peter Batzel[2], Jason Sydes[2], Tom Titus[2], Catherine A. Wilson[2], Julian M. Catchen[5], Wesley C. Warren[6], Manfred Schartl[7,8,9]*, H. William Detrich III[10]*, John H. Postlethwait[2]* and Hyun Park[1,4]*

[1]Unit of Polar Genomics, Korea Polar Research Institute, Incheon, Korea. [2]Institute of Neuroscience, University of Oregon, Eugene, OR, USA. [3]Department of Polar Life Science, Korea Polar Research Institute, Incheon, Korea. [4]Polar Science, University of Science and Technology, Daejeon, Korea. [5]Department of Animal Biology, University of Illinois, Champaign, IL, USA. [6]McDonnell Genome Institute, Washington University, St. Louis, MO, USA. [7]Department of Developmental Biochemistry, Biocenter, University of Wuerzburg, Wuerzburg, Germany. [8]Hagler Institute for Advanced Study, Texas A&M University, College Station, TX, USA. [9]Department of Biology, Texas A&M University, College Station, TX, USA. [10]Department of Marine and Environmental Sciences, Northeastern University Marine Science Center, Nahant, MA, USA. *e-mail: phch1@biozentrum.uni-wuerzburg.de; w.detrich@northeastern.edu; jpostle@uoneuro.uoregon.edu; hpark@kopri.re.kr

Antarctic blackfin icefish genome reveals adaptations to extreme environments

Bo-Mi Kim[1], Angel Amores[2], Seunghyun Kang[1], Do-Hwan Ahn[1], Jin-Hyoung Kim[1], Il-Chan Kim[3], Jun Hyuck Lee[1,4], Sung Gu Lee[1,4], Hyoungseok Lee[1,4], Jungeun Lee[1,4], Han-Woo Kim[1,4], Thomas Desvignes[2], Peter Batzel[2], Jason Sydes[2], Tom Titus[2], Catherine Wilson[2], Julian M. Catchen[5], Wesley C Warren[6], Manfred Schartl[7,8,*], H. William Detrich III[9,*], John H. Postlethwait[2,*], Hyun Park[1,4,*]

S1 Transposable element annotation

The icefish genome contains 50.36% repeat sequences including 47.41% TE elements such as LTR (5.58%), SINEs (0.58%), LINEs (7.25%) and DNA transposons (15.07%) (Supplementary Figure 15 and Supplementary Table 7). The size of the icefish genome is the largest reported to date for Antarctic notothenioid fishes. As the relative TE contribution impacts genome size, the relative contribution of major types of TEs, that is, LTR, LINE, and SINE retrotransposons as well as DNA transposons, was estimated in several fish genomes. According to their TE composition, most genomes showed a predominance of DNA transposons except tetraodon and takifugu. Some TE superfamilies, including gypsy, DIRs, L2, Rex retrotransposon, as well as Harbinger, hAT, P, PIF, PiggyBac and Heliton DNA transposons were detected in the majority of *C. aceratus*, but DRE, R1, R2 retrotransposon and IS3EU DNA transposon were not detected in the icefish genome (Supplementary Figure 16). Kimura distances (K-values) were calculated for all TE copies of each element in order to estimate the "relative age" and transposition history of TEs[1]. Copy divergence is correlated with the age of activity: Very similar copies (low K-values) are indicative of rather recent activity (on the left part of the graph), while divergent copies (high K-values) have been generated by more ancient transposition events (on the right part of the graph). Results were grouped for the four different types of TEs (DNA transposons, LTR, LINE, and SINE retrotransposons) (Supplementary Figure 17). Teleost genomes generally contain fewer ancient copies (K-values >25), and significant interspecific differences in profiles were observed with generally one or two general bursts of transposition. Antarctic fishes and stickleback genome are dominated by rather recent copies (K-values <5) and strongly shaped by DNA transposons, which can be taken as indication of recent bursts of transposition in Antarctic fishes. TE expansion may have facilitated gene duplication and other events of genome evolution in particular periods of evolutionary history and thus may have contributed to adaptation to the specifics of Antarctic environments.

S2 Expansion of Olfactory receptors

Olfactory receptor (*or*) genes were annotated by manual inspection from the blackfin icefish genome. In total, 212 *or* genes were annotated and classified into 11 subclasses based on phylogenetic distance. Of the 212, 161 genes (≈ 76%) were classified into the 'delta' class, which is known to detect water-soluble odorants[2]. When the total number of blackfin icefish *or* genes was compared to those of five teleosts that were previously annotated[2], we found that blackfin icefish has the largest number of *or* genes among these fish (Supplementary Table 21). In addition, sensory system related GO terms such as *sensory perception of chemical stimulus* (GO:0007606) and *sensory perception* (GO:0007600) in the biological process category and *olfactory receptor activity* (GO:0004984) in the molecular function category were enriched in the expanded *or* gene dataset of the blackfin icefish genome (Supplementary Table 10).

Supplementary Fig. 1. Photograph of a female specimen of the Antarctic blackfin icefish, *Chaenocephalus aceratus.*

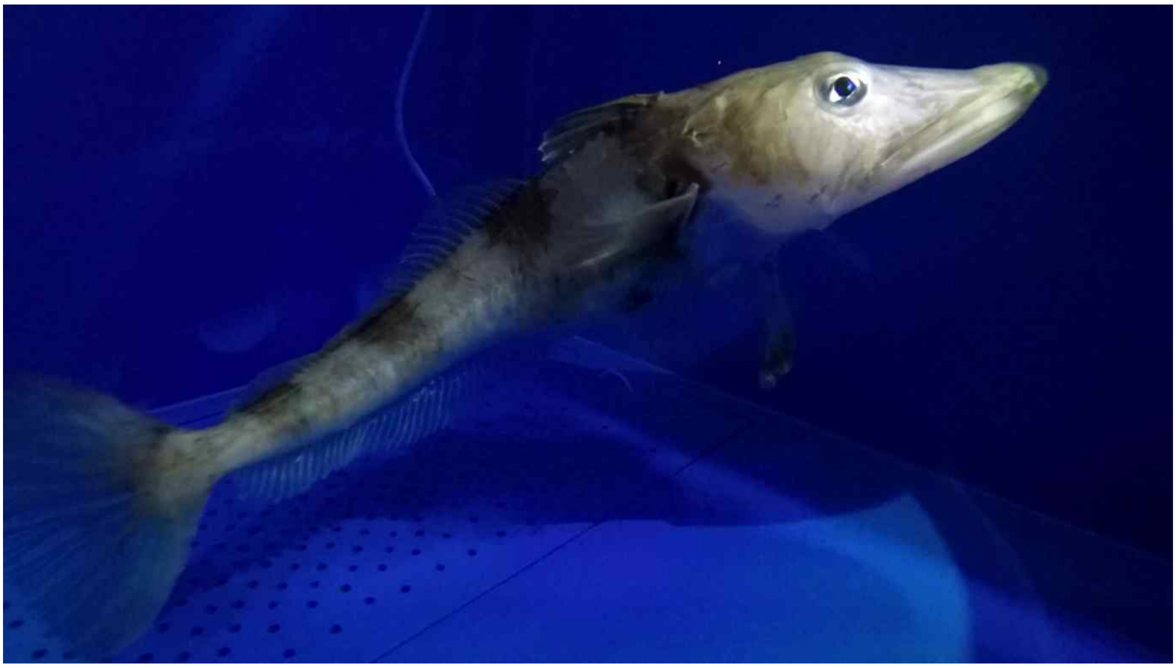

Supplementary Fig. 2. Sampling sites for Antarctic blackfin icefish. The red dot shows the sampling location for the specimen used in genome and transcriptome sequencing, and the red triangle represents the sampling site for fish used to make the genetic linkage map and for the microRNA-seq experiments. Map was based on the updated Figure 1 of Park et al. (2010)[3].

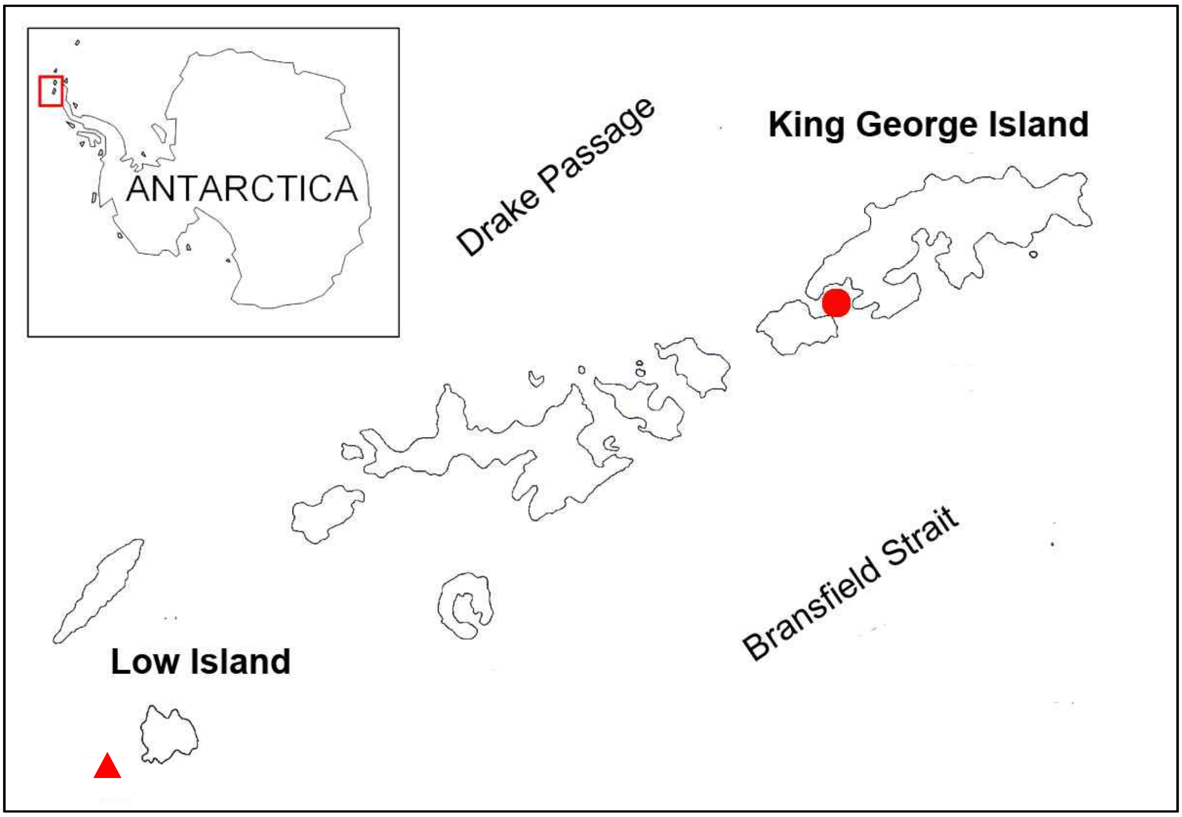

Supplementary Fig. 3. Estimation of genome size by K-mer analysis. Genome size was calculated as total error-corrected PacBio sequence. The estimated genome size of *C. aceratus* is 1.1 Gb (81-mers).

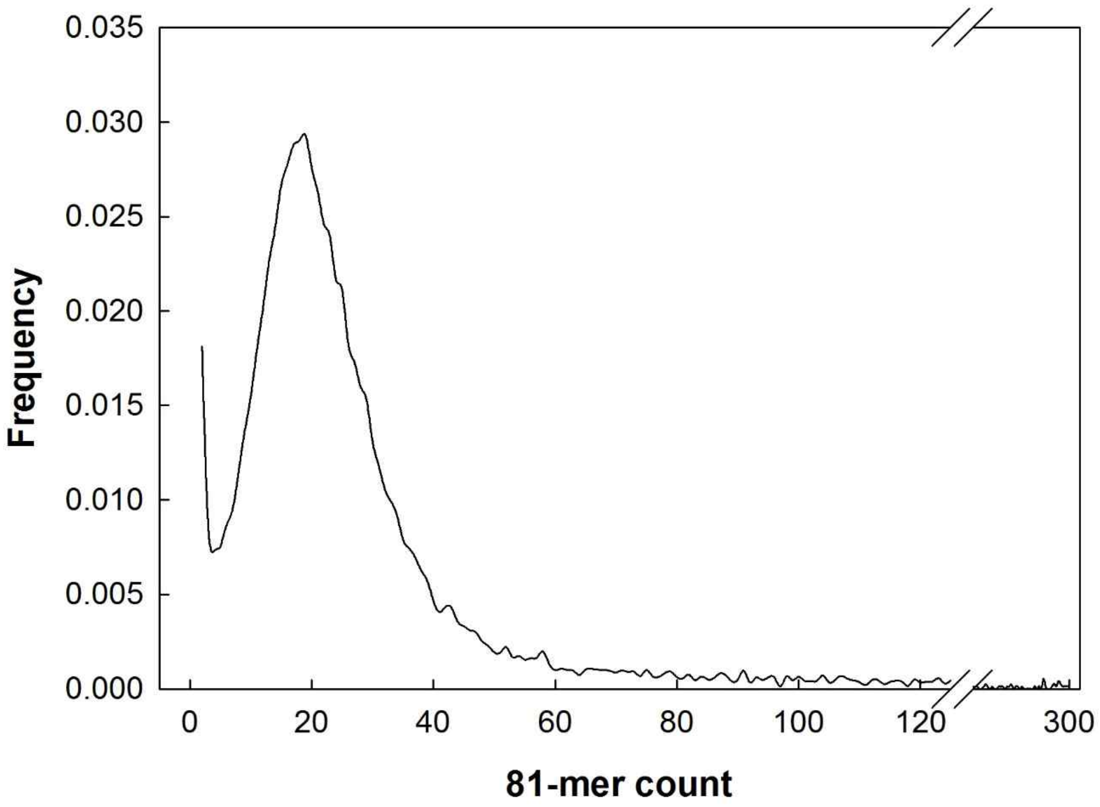

Supplementary Fig. 4. Genetic linkage map for the blackfin icefish.

Supplementary Fig. 4. Genetic linkage map for the blackfin icefish.

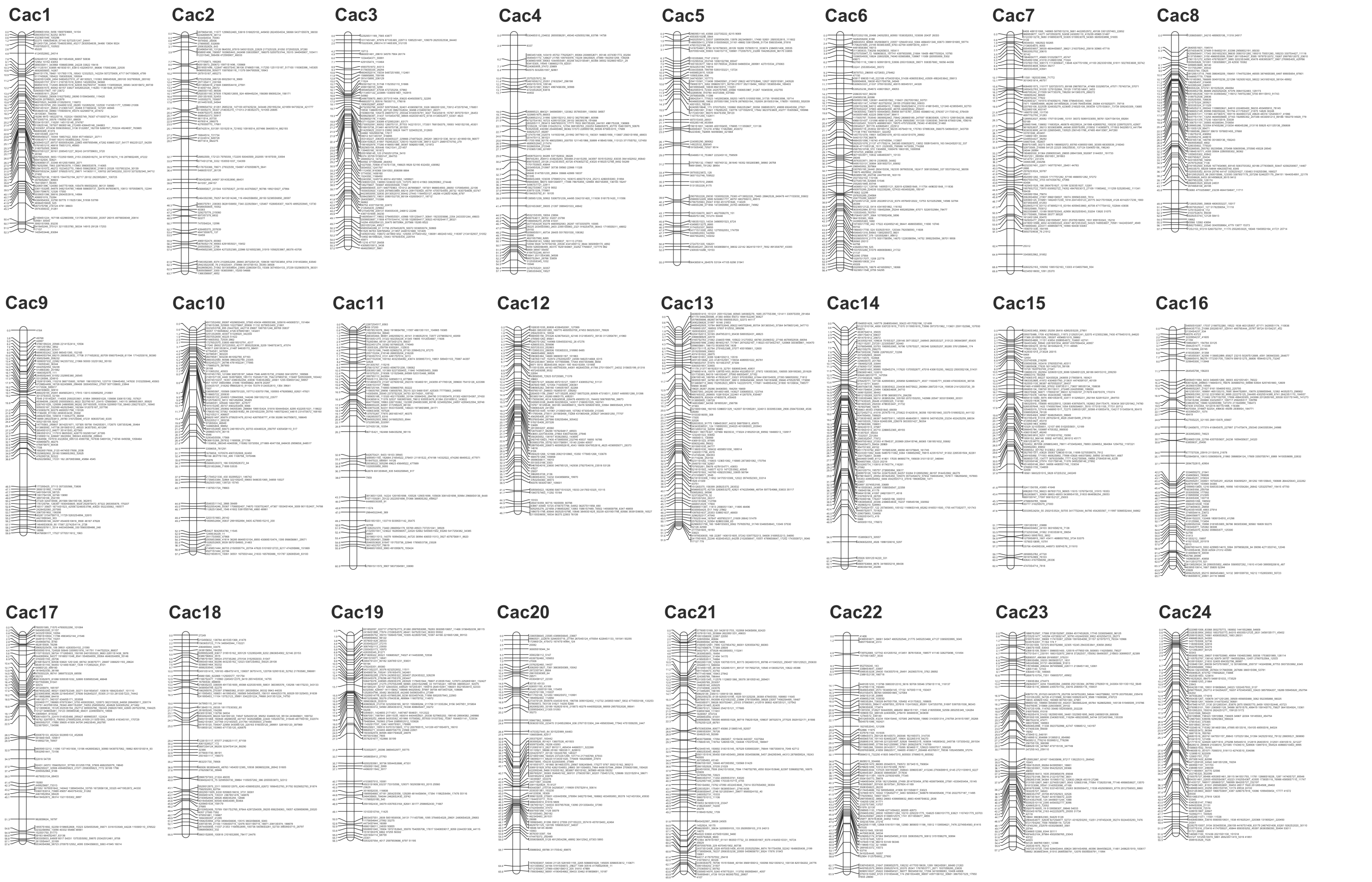

Supplementary Fig. 5. Conserved synteny of *C. aceratus* chromosomes with other teleosts. a. Analysis of gene content shows a one-to-one correspondence between icefish and European sea bass chromosomes. Icefish-specific color-coded lines connect predicted orthologous genes and show that no translocations occurred in the icefish lineage after it diverged from the European sea bass lineage. b. Conservation of synteny between icefish chromosome Cac12 (LG12) and stickleback linkage group LGXIV (Gac14). Lines connecting individual orthologous genes are color coded based on the icefish genomic contig. c. Conserved synteny between sea bass LG19 (Dla19) and stickleback LGXIV (Gac14), which reveals that inversions and transpositions were frequent in the icefish lineage *after* it diverged from the sea bass lineage. d., e. The only detectable translocation in the icefish lineage involved a small section of the ancestral chromosome corresponding to medaka Ola6 and sea bass Dla6 being moved to a portion corresponding to Ola7 and Dla22-25 to provide icefish chromosome Cac7. Dot plots from Synolog (http://catchenlab.life.illinois.edu/chromonomer/) show orthologs and paralogs of genes along Cac7 in the order they appear on Cac7 with a dot directly above the positon of the icefish gene. Orthologous chromosome segments have many orthologs, while paralogous chromosome segments have a few paralogs plotted.

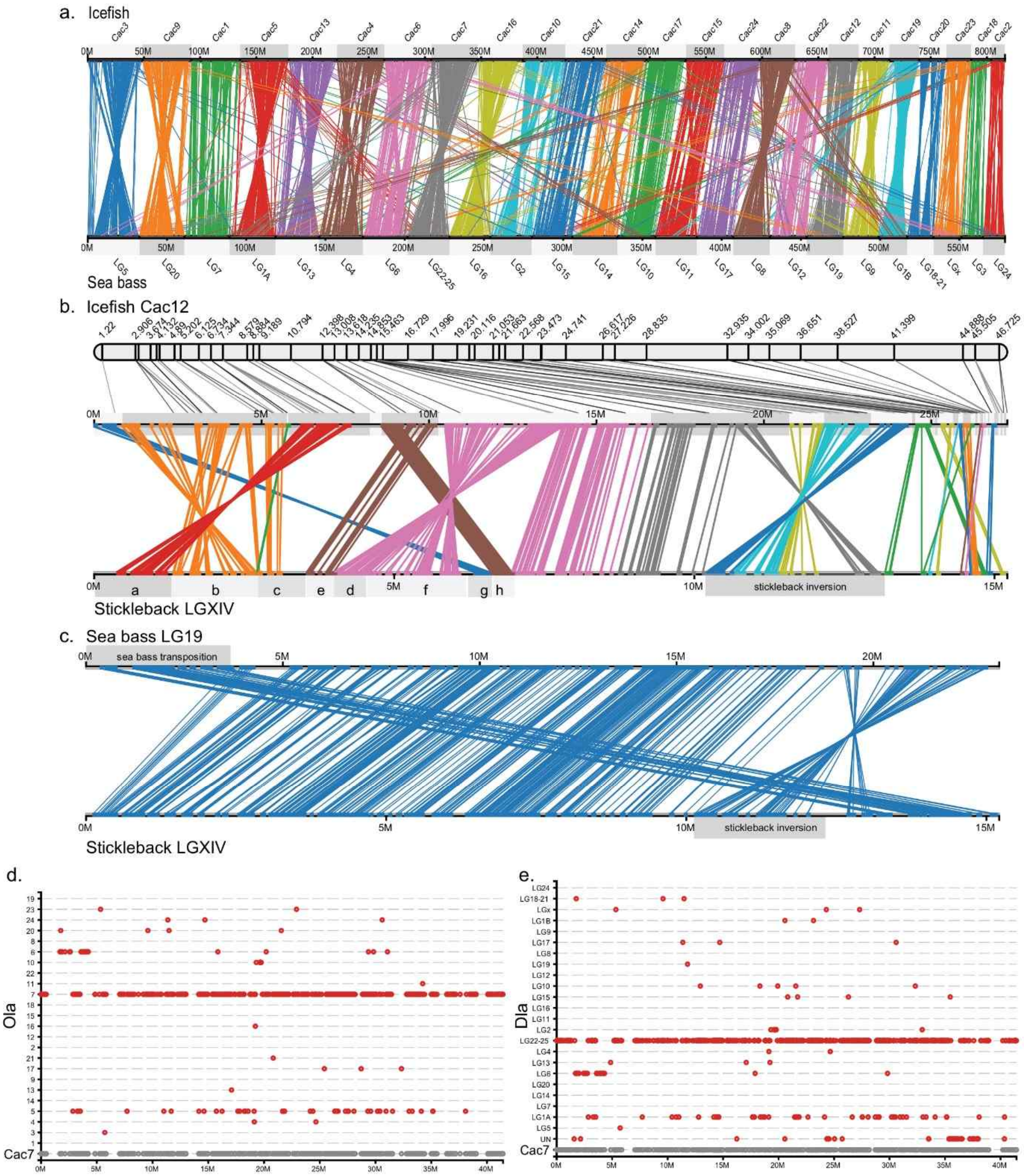
a. Icefish
Cac3 Cac9 Cac1 Cac5 Cac13 Cac4 Cac6 Cac7 Cac16 Cac10 Cac21 Cac14 Cac17 Cac15 Cac24 Cac8 Cac22 Cac12 Cac11 Cac19 Cac20 Cac23 Cac18 Cac2
0M 50M 100M 150M 200M 250M 300M 350M 400M 450M 500M 550M 600M 650M 700M 750M 800M
0M 50M 100M 150M 200M 250M 300M 350M 400M 450M 500M 550M
LG5 LG20 LG7 LG1A LG13 LG4 LG6 LG22-25 LG16 LG2 LG15 LG14 LG10 LG11 LG17 LG8 LG12 LG19 LG9 LG1B LG18-21 LGx LG3 LG24
Sea bass
b. Icefish Cac12
0M 5M 10M 15M 20M 25M
0M a b c e d 5M f g h 10M stickleback inversion 15M
Stickleback LGXIV
c. Sea bass LG19
0M sea bass transposition 5M 10M 15M 20M
0M 5M 10M stickleback inversion 15M
Stickleback LGXIV
d.
Ola
Cac7
0M 5M 10M 15M 20M 25M 30M 35M 40M
e.
Dla
LG24 LG18-21 LGx LG1B LG9 LG17 LG8 LG19 LG12 LG10 LG15 LG16 LG11 LG2 LG22-25 LG4 LG13 LG6 LG20 LG14 LG7 LG1A LG5 UN
Cac7
0M 5M 10M 15M 20M 25M 30M 35M 40M

Supplementary Fig. 6. Conservation of gene order between orthologous icefish and sea bass chromosomes. a, icefish LG1. b, icefish LG9. c, icefish LG16. d, icefish LG20. Lines connecting orthologous genes are color coded relative to the icefish genomic contig. Comparisons show many inversions and a few transpositions that distinguish these two species, whose lineages diverged more than 100 million years ago.

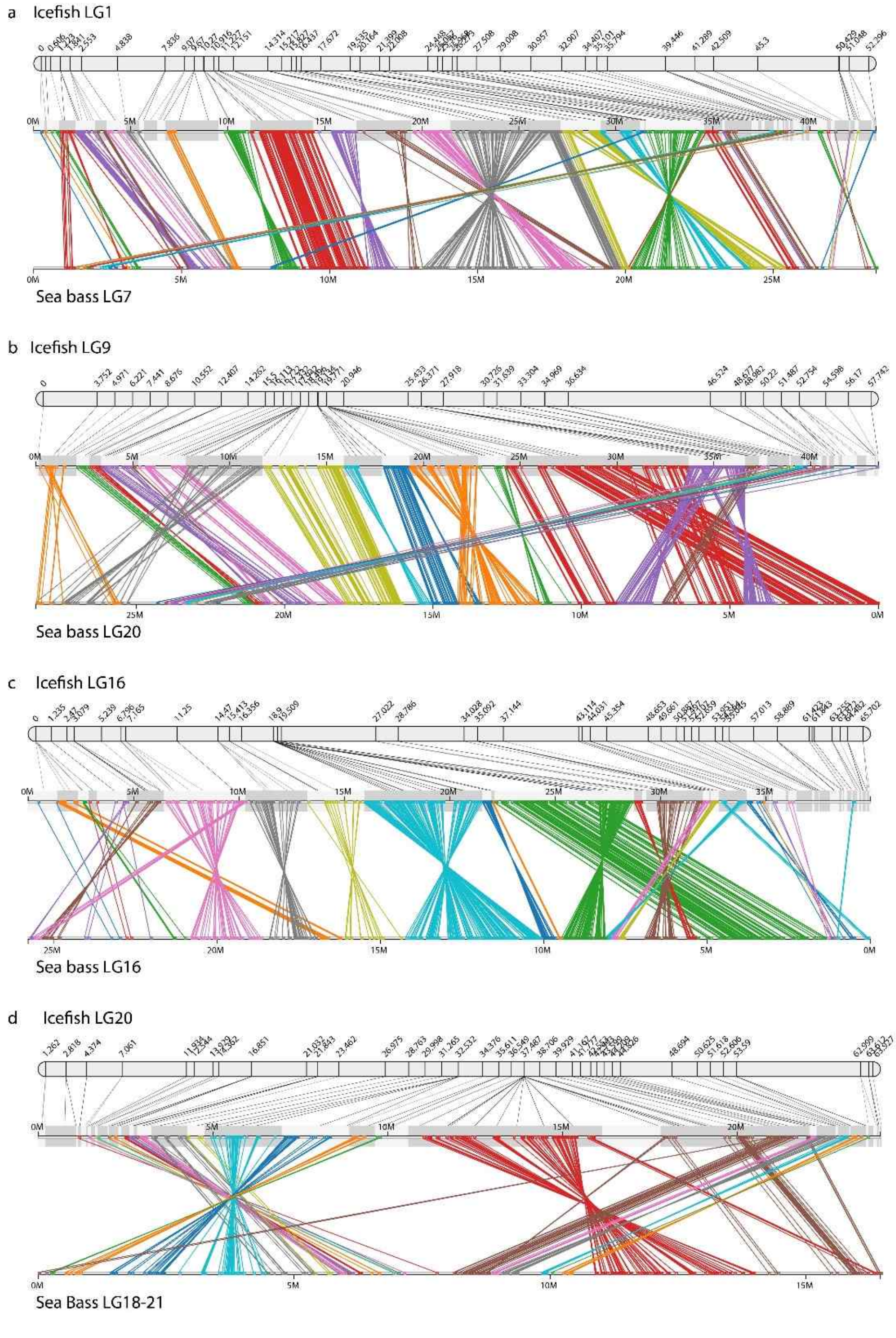

Supplementary Fig. 7. Divergence time estimation of teleost fish. Blue bars depict 95% highest posterior density (HPD) time estimates (time scale: Million years ago).

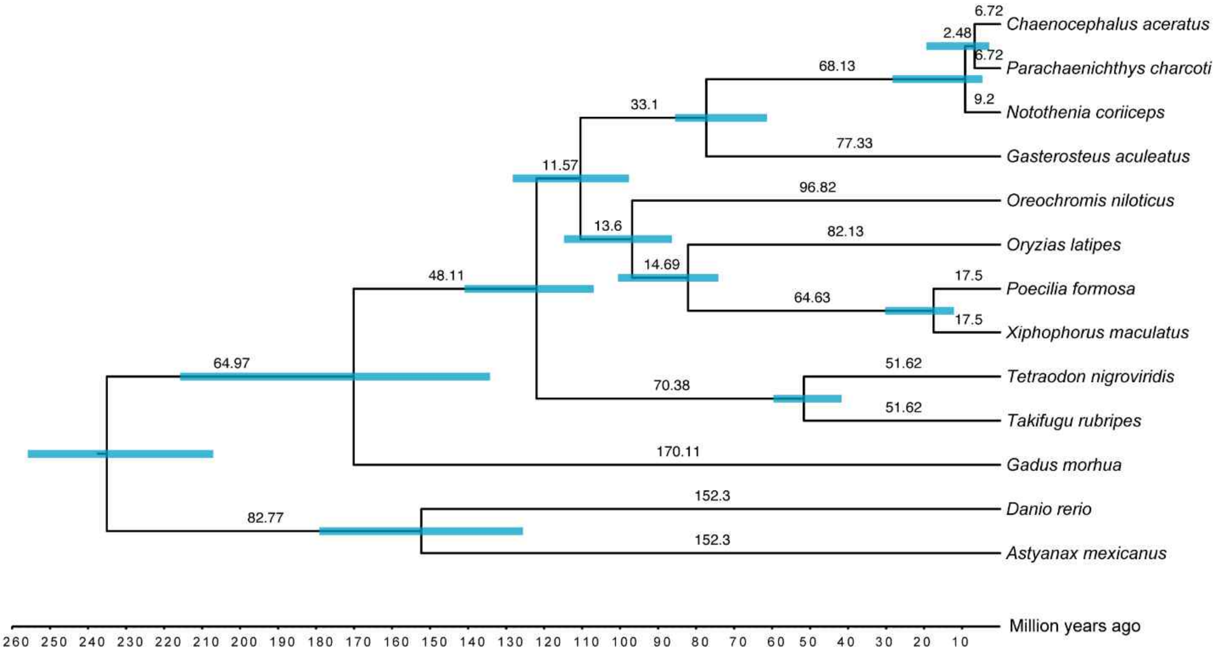

Supplementary Fig. 8. Venn diagram of orthologous gene clusters for six teleost fishes.

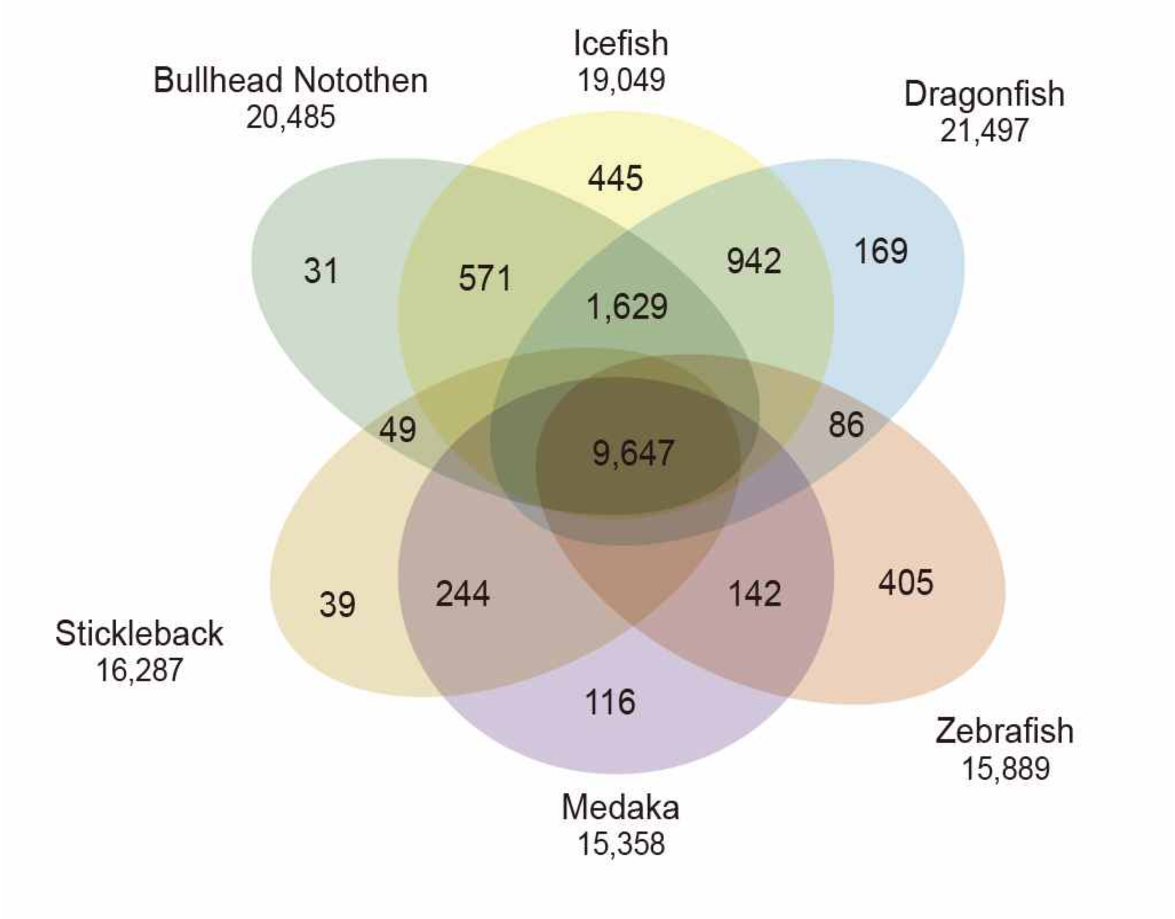

Supplementary Fig. 9. **Conserved syntenies for zpc3 and zpc5 genes identified in the icefish genome**. The icefish *zpc3 and zpc5* genes are tandemly duplicated in Ice_000114.

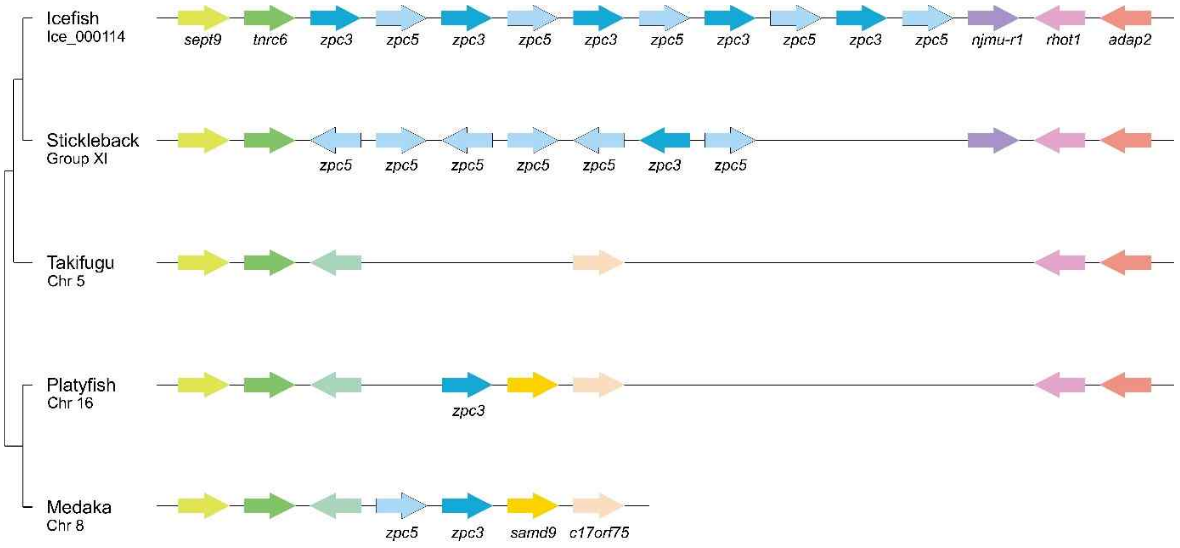

Supplementary Fig. 10. **Transcriptional profile of zona pellucida (zp) protein family genes in blackfin icefish organs**. The transcriptional profile of *zp* genes was measured in twelve organs of icefish (i.e. brain, eye, gill, heart, intestine, kidney, liver, muscle, ovary, skin, spleen, and stomach). The level of expression for each mRNA is represented by color gradations in the heat map.

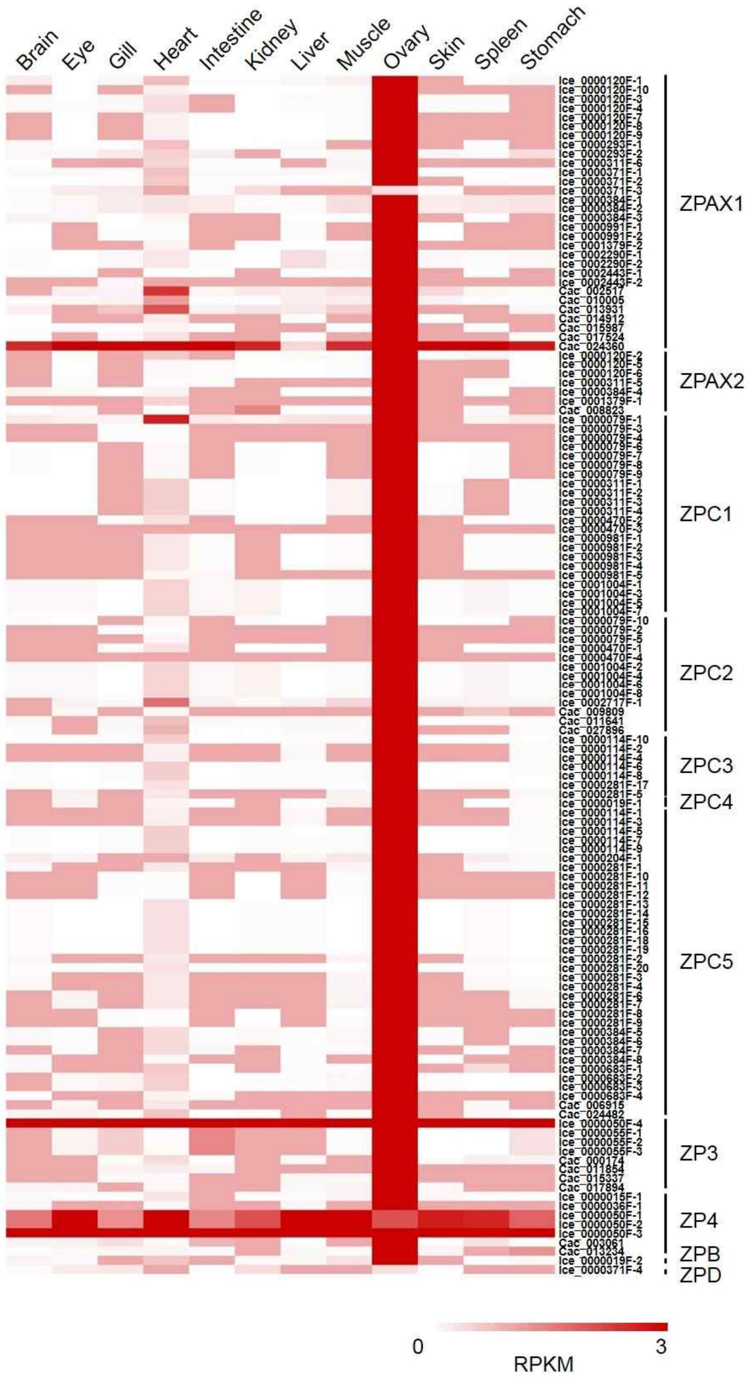

Supplementary Fig. 11. **Syntenic comparisons of icefish globin gene clusters**. Teleosts have two globin gene clusters. The LA cluster, which encodes both hemoglobin α- and β-subunits, is adjacent to the *lcmt1* and *aquaporin* genes on one side and *rhbdf1b* on the other side. The MN cluster, which also encodes both hemoglobin α- and β-subunits, has *rhbdf1a, mpg,* and *nrpl3* on one side and *kank2* on the other side. Loss of complete copies of α- and β-globin genes is observed for both the LA and MN clusters in the icefish genome. Each arrow indicates a gene in the 5'→3' direction. α- and β-globin clusters are shown by green and red boxes. The partial alpha globin gene containing only exon 3 is identified in the icefish LA cluster by a dashed box . This figure was updated from figure 1 of Opazo et al.[4].

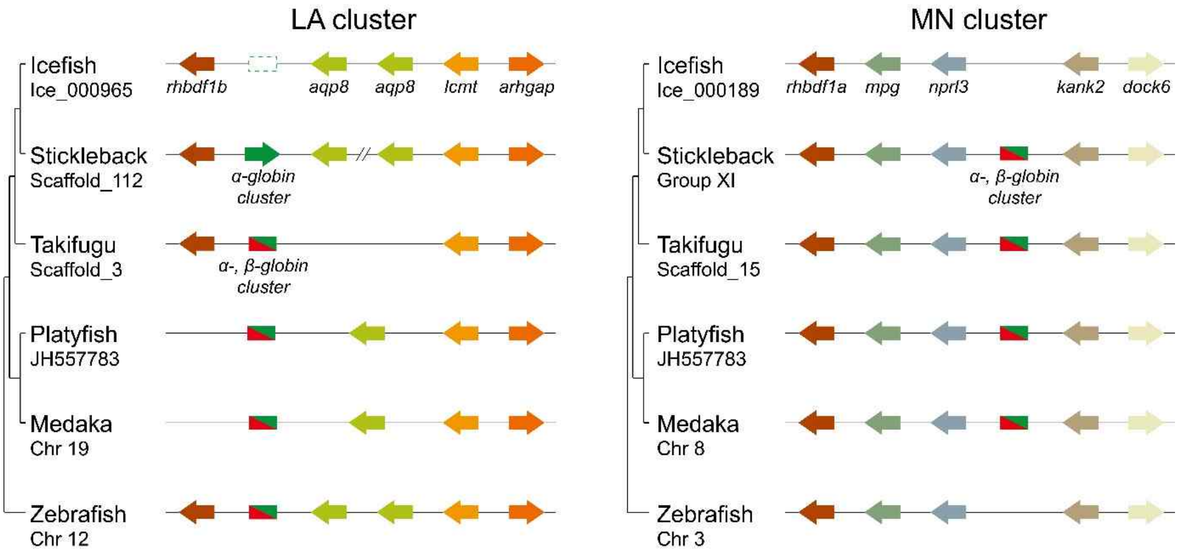

Supplementary Fig. 12. **Syntenic comparison of myoglobin and cytoglobin genes identified in icefish genome**. Genomic structure and syntenic comparisons for **a**. cytoglobin (*cygb1*) **b**. *cygb2*, **c**. myoglobin (*mb*) and **d**. neuroglobin (*ngb*) genes are shown within representative sequenced teleost genomes.

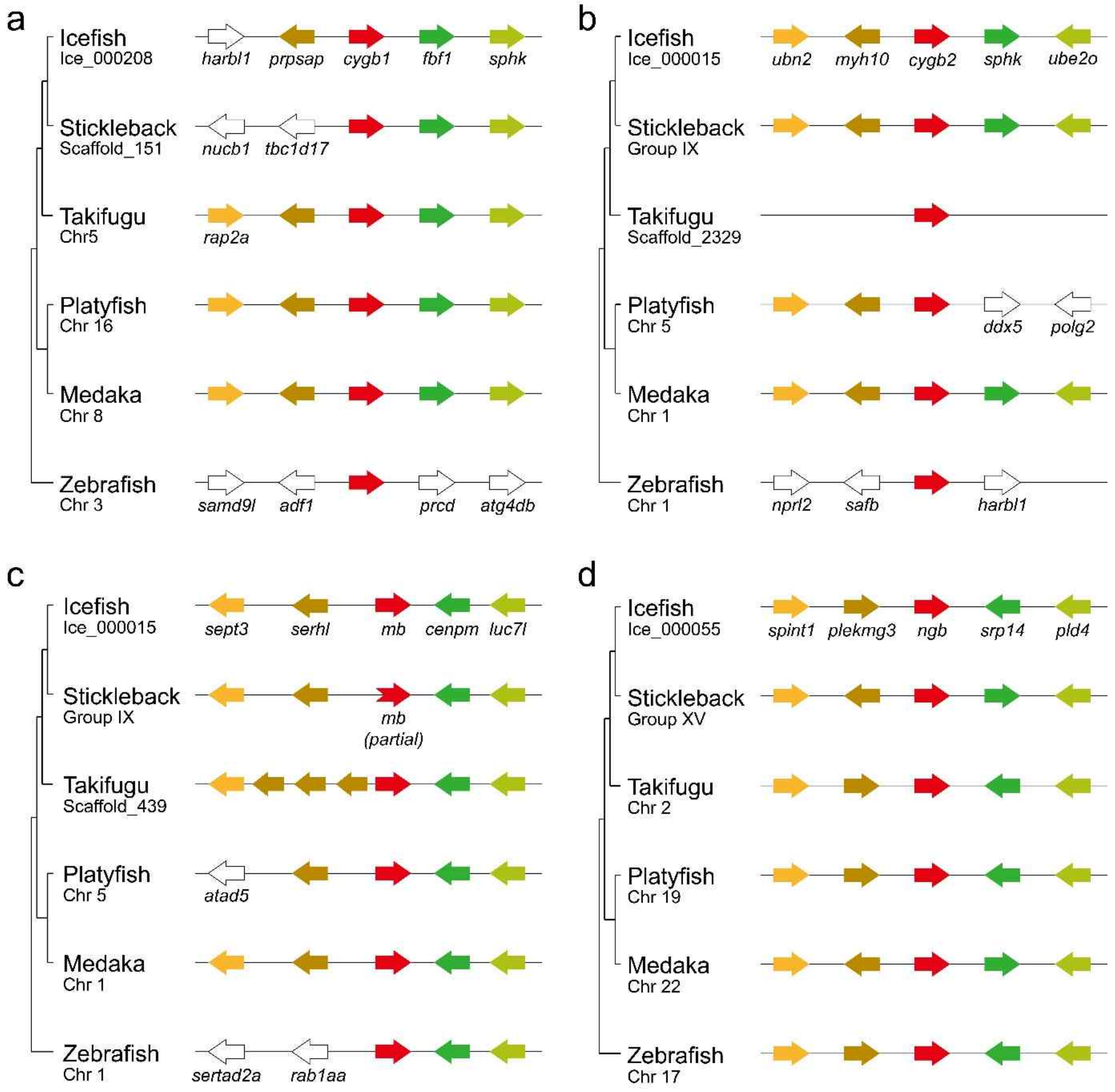

Supplementary Fig. 13. Cladogram depicting expansion of NAD(P)H quinone dehydrogenase 1 (*nqo1*) genes in the icefish genome. Comparison of genomic structure, conserved synteny, and phylogenetic relationships of icefish *nqo1* genes shown within representative sequenced teleost genomes. Icefish *nqo1* genes are distributed on five different contigs.

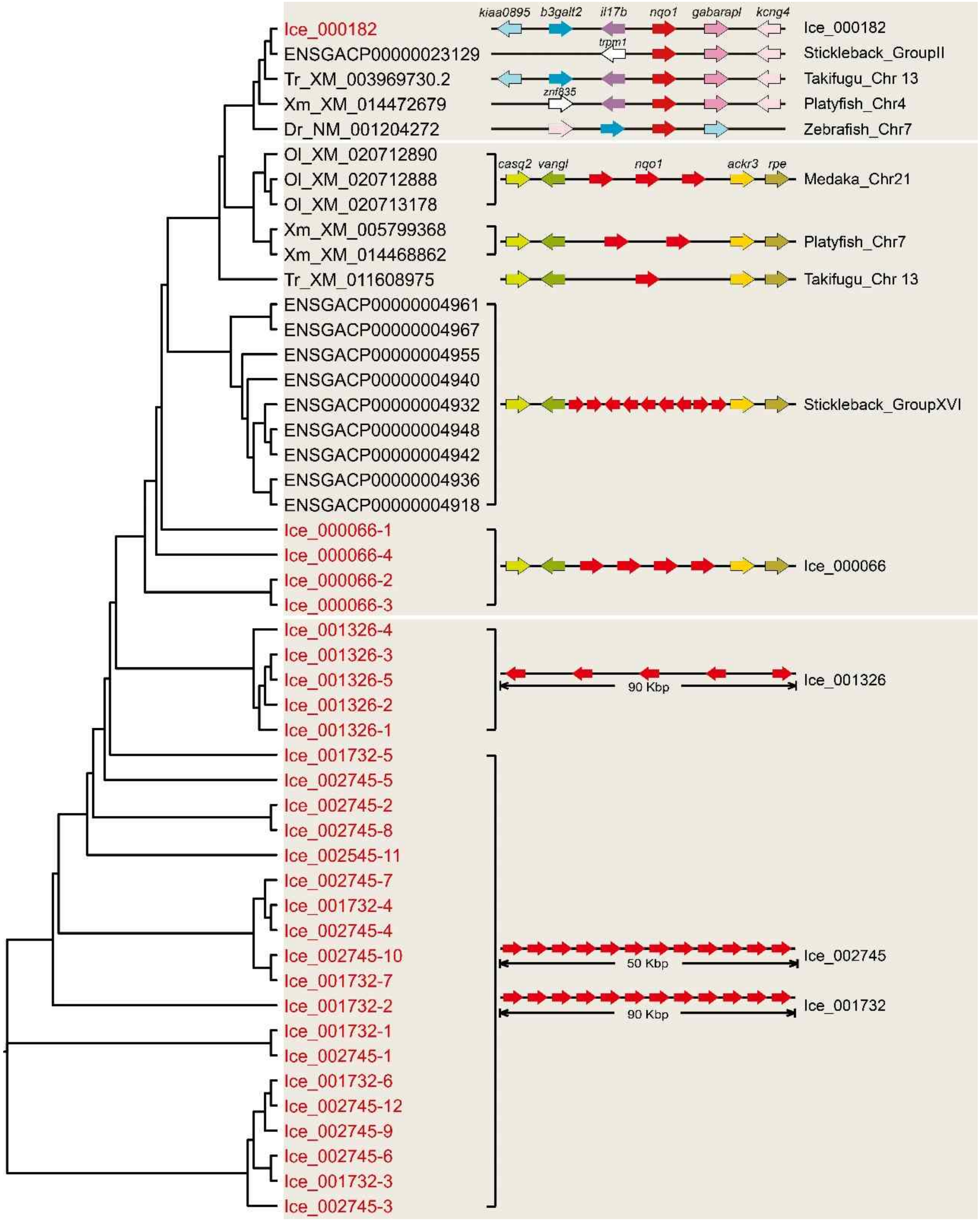

Supplementary Fig. 14. **The 8-oxoguanine DNA glycosylase (*ogg1*) gene is duplicated in the icefish genome**. Other teleost genomes contain a single gene.

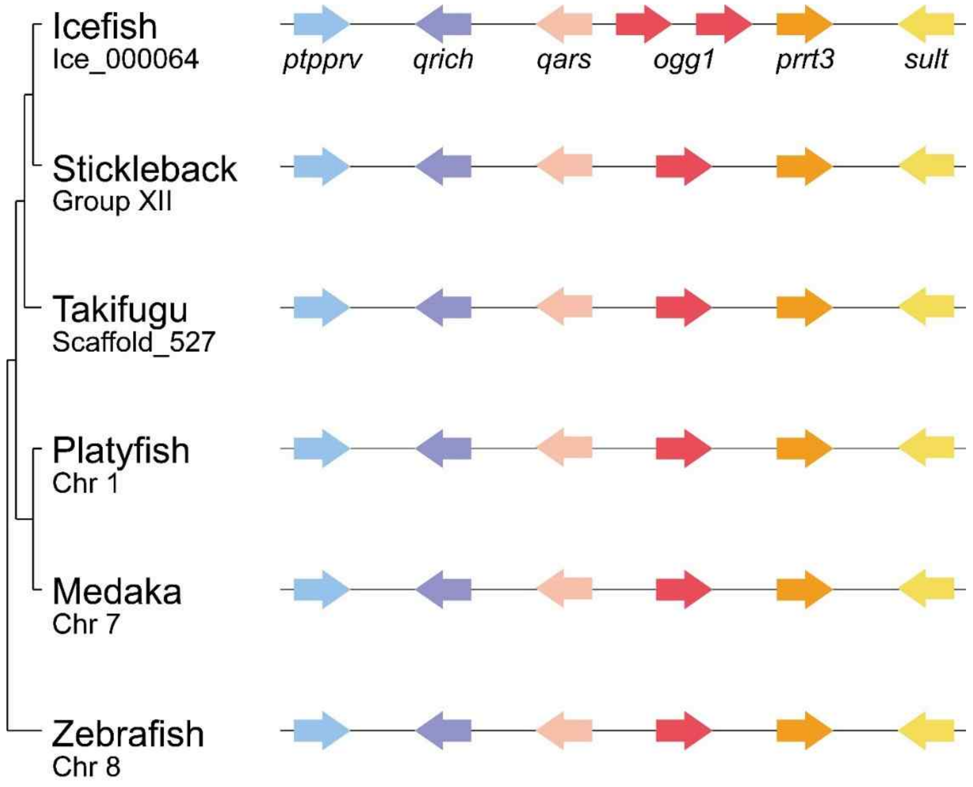

Supplementary Fig. 15. **Percentages of TEs in teleost genomes.** The amounts of LTR, SINE and LINE retrotransposons, DNA transposons, and unclassified elements (Unknown), as well as their respective proportions were estimated based on RepeatMasker outfiles.

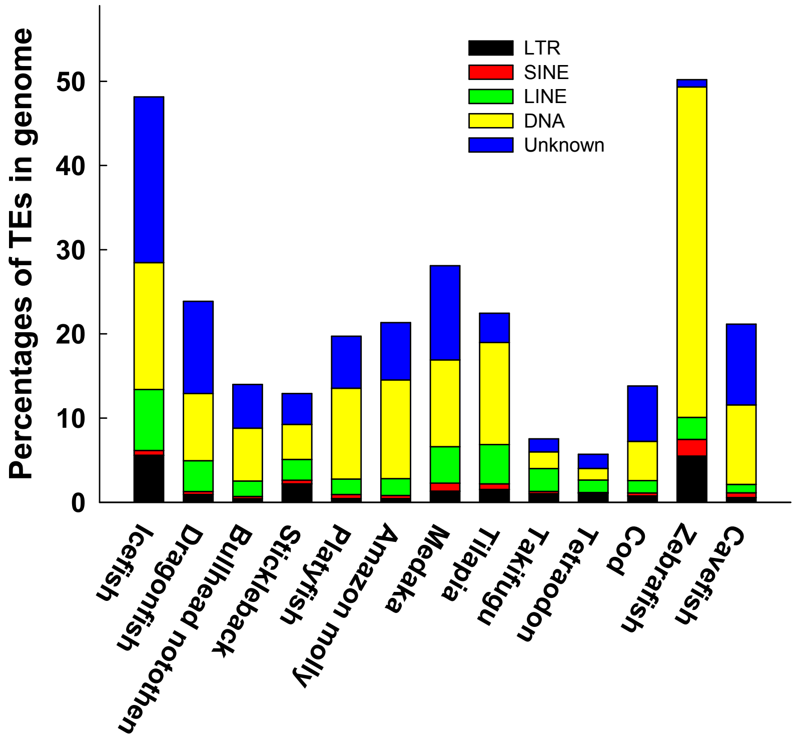

Supplementary Fig. 16. **Diversity and abundance of TE superfamilies in teleosts.** Presence or absence of TE superfamilies was determined using automatic annotation, manual verification, and literature information. The relative proportion of superfamilies is shown in squares according to the gradient scale (log) at the bottom.

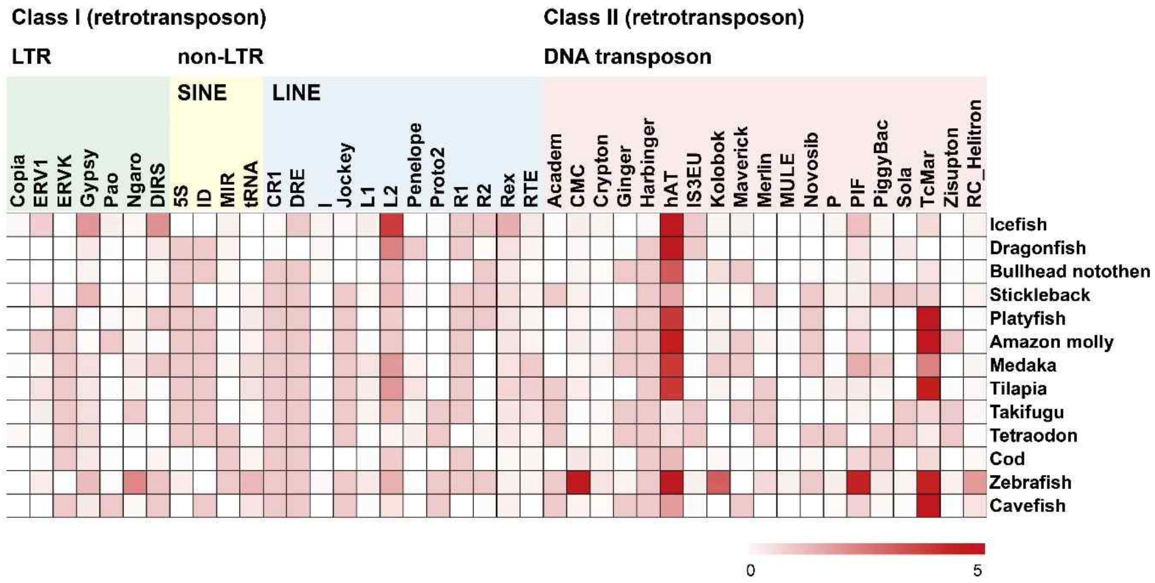

Supplementary Fig. 17. **Kimura distance-based copy divergence analysis of transposable elements in teleost genomes.** Graphs represent genome coverage (Y-axis) for each type of TE (DNA transposons, SINE, LINE, and LTR retrotransposons) in the different genomes analyzed, clustered to their corresponding consensus sequence according to Kimura distances (X-axis, K-value from 0 to 50). Right inserted square shows axes for all graph.

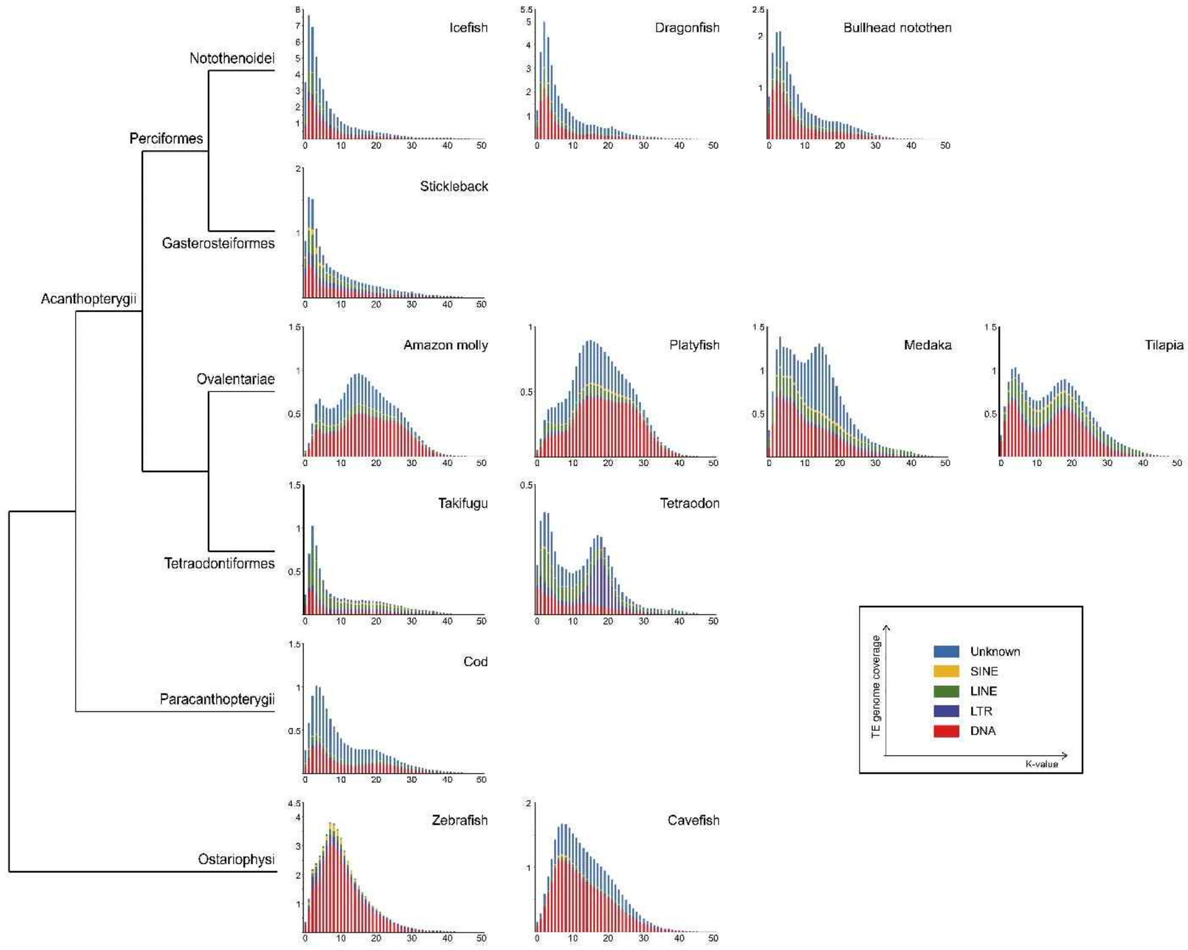

Supplementary Table 1. Statistics for icefish genome sequencing using PacBio Sequel.

Library	No. of Cells	No. of Reads	Total data (Mbp)	N50 read length (bp)	Mean read length (bp)
Polymerase read	16	6,562,134	89,275	22,250	13,605
Subread		8,434,974	89,045	16,203	10,557

Supplementary Table 2. BUSCO scores for the completeness of the icefish genome sequence. (http://busco.ezlab.org)

	Actinopterygii Gene Set	%
Complete BUSCOs (C)	4,123	89.9
Complete and single-copy BUSCOs (S)	3,930	85.7
Complete and duplicated BUSCOs (D)	193	4.2
Fragmented BUSCOs (F)	164	3.6
Missing BUSCOs (M)	297	6.5
Total BUSCO groups searched	4,584	

Supplementary Table 3. Sequencing statistics for icefish transcriptome analysis.

Organ	No. reads	Total data (bp)	% reads mapping*
Brain	42,517,602	4,294,277,802	94.76
Eye	48,799,334	4,928,732,734	95.45
Gill	51,072,374	5,158,309,774	94.35
Heart	56,781,088	5,734,889,888	92.23
Intestine	51,113,066	5,162,419,666	96.66
Kidney	53,863,870	5,440,250,870	90.35
Liver	78,598,880	7,938,486,880	86.68
Muscle	45,374,108	4,582,784,908	87.23
Ovary	49,080,678	4,957,148,478	91.09
Skin	45,497,130	4,595,210,130	89.49
Spleen	43,778,336	4,421,611,936	89.57
Stomach	57,809,718	5,838,781,518	86.44

*Data from each organ was mapped to assembled icefish genome using Tophat.

Supplementary Table 4. General statistics of the functional annotation.

		Number	Percent (%)
Total		30,773	
Annotated	Blastp	23,923	77.74
	Interpro	20,684	67.21
	GO	16,671	54.17
	KEGG	19,893	64.64

Supplementary Table 5. Sequencing statistics for icefish small-RNA transcriptome analysis.

Organ	Number of reads retained for analysis*	Number of reads being annotated	% reads being annotated
Pronephric kidney	4,818,658	4,281,988	88.86
Pectoral girdle bone	9,459,845	7,820,112	82.67
Heart ventricle	596,018	545,265	91.48
Pectoral adductor muscle	2,715,548	2,343,132	86.29
Skeletal muscle	5,750,328	4,822,711	83.87

*Passed sequence quality filter; passed size filter; have minimum of 5 counts

Supplementary Table 6. Information of miRNA sequence of blackfin icefish.

Gene Name	Sequence	Location	Strand
cac-let-7a-2	GCTCCCAGGTTGAGGTAGTAGGTTGTATAGTTGAGAATTACACCCCGGG AGATAACTGTACAACCTCCTAGCTTTCCCTGAGGTAC	LG13:9475996-9476081	+
cac-let-7a-3	TCCTTTTGGGTGAGGTAGTAGGTTGTATAGTTTTAGGGTCAATCCCGAG CTGTAAAATGACTATACAATTTACTGTCTTTCCTGAAGCGACC	LG23:16144495-16144586	+
cac-let-7a-4	TCCCTCAGGTTGAGGTAGTAGGTTGTATAGTTCAGAGTGACACCTCAGG AGATAACTGTACAGCCTCCTAGCTTTCCCTGAGGAAAC	LG14:29507401-29507487	-
cac-let-7a-5	TCTTTCAGGGTGAGGTAGTAGGTTGTATAGTTTGGTGGGTGGGACTGCA CCCTGCTCAGGTGATAACTATACAGTCTATTGCCTTCCCTGAGGAGTC	LG7:23011503-23011599	+
cac-let-7a-6	CCTGGCAAGGTGAGGTAGTAGGTTGTATAGTTTGTGGGATGGAGTAAA TCCTACTCAGGCGATAACTATACAGTCTAATACCTTCTTGAGGGGTACA ATGA	LG5:14697071-14697171	+
cac-let-7a-7	TCCTTTGGGGTGAGGTAGTAGGTTGTATAGTTTTAGGGTCATACCCTTC CTGTCAGATAACTATACAACTTACTGTCTTTCCTGAAGTGGCTG	LG6:31060983-31061075	-
cac-let-7b-1	TCGTACAGGGTGAGGTAGTAGGTTGTGTGGTTTCAGGGTTGTGATTGTA CCCCATCAGGAGCTAACTGTACAACCTACTGCCTTCCCTGGAGGGC	LG23:16145238-16145332	+
cac-let-7b-2	TCGAACAGGGTGAGGTAGTAGGTTGTGTGGTTTCAGGGTAGTGATTTTG CCCAATAAGGAGATAACTGTACAACCTACCGCCTTCCCTGAAGGGCA	LG6:31060415-31060510	-
cac-let-7c-2	GCATCCGGGTTGAGGTAGTAGGTTGTATGGTTTAGAATGACATCCTGGG AGTTAACTGTACAACCTTCTAGCTTTCCTTGGAGCAC	LG14:5184609-5184694	+
cac-let-7d-1	CTCTGCAGTGTGAGGTAGTTGGTTGTATGGTTTCGCATAATAAACAGCA CGGAGATAACTCTACAACCTTCTAGCTTTCCCTGCGGAGTC	LG16:19224686-19224775	+
cac-let-7d-2	CTCTGCAGTTTGAGGTAGTTGGTTGTATGGTTTCGCATCATAAACAGCC TGGAGATAACTGTACAACCTTCTAGCTTTCCCTGCGGCGT	Ice_Fa_000667F:208836-208924	-
cac-let-7e-1	TCCTTGGGGCTGAGGTAGTAGATTGAATAGTTGTGGGGTTGTGTAACCT CTTTTTGAGATAACTATACAATCTACTGTCTTTCCCAAGGAGAC	LG7:23011252-23011344	+
cac-let-7e-2	TCCTTAAGGCTGAGGTAGTAGATTGAATAGTTGTGGGGTTTTTGTCCTC CCTCTGAGATAACTATACAATCTACTGTCTTTCCTAAGGAGAC	LG5:14696745-14696836	+
cac-let-7g-1	AGCTGTGGGATGAGGTAGTAGTTTGTATAGTTTTAGGATCACACCAGAT CTGGGAGATAACTATACAGCCTACTCTCTTTCTCACGGCAACC	LG7:32952936-32953027	-
cac-let-7g-2	TGCTGTGGGATGAGGTAGTAGTTTGTATAGTTTTAGGATCACACCAGAT CTGGGAGATAACTATACAGTCTACTGTCTTTCTTATGGCTACG	LG7:21074106-21074197	+
cac-let-7h-1	TGTGCTGTGGTGAGGTAGTAAGTTGTGTTGTTGTTGGGGATAAGGATTG TTCACCCCGTTCAGGAGATAACTATACAACTTACTGCCTTCCTCAGTGC AGGC	LG7:21074284-21074385	+
cac-let-7i-1	ATGCACTGGCTGAGGTAGTAGTTTGTGCTGTTGGTTGGGTTGTGACACT GCCCGCTATGGAGATGACTGCGCAATCTACTGCCTTGCTAGTGCTGGT	LG6:31422588-31422684	+
cac-let-7i-2	ATGCGCTGGTTGAGGTAGTAGTTTGTGCTGTTGGTCGGGTTGTTATATT ACCCCTTGTGGAGATGACTGCGCAATCTACTGCCTTGCTGGCGCTGGT	LG23:15956748-15956844	-
cac-let-7j	CACTTTGGTCTGAGGTAGTTGTTTGTACAGTTTGAGGGTCTGTGATTCTG CCCCATACAGGAGCTAACTGTACAACTGACTGCCTTGCCCACGGTTGA	LG5:25524602-25524699	-
cac-mir-100-1	CATGGTCCCAAACCCGTAGATCCGAACTTGTGTTATCTGACACCACAAG CTTGTGTCTACAGGTCTGCGTCTG	LG13:9475205-9475277	+
cac-mir-100-2	AGCTGCCACAAACCCGTAGATCCGAACTTGTGGTGACTGGCTGCACAA GCTCGTATCTATAGGTATGTGTCTTCCTG	LG14:29508344-29508420	-
cac-mir-101a	CTTCCCTGGTTCAGTTATCACAGTGCTGATGCTGTCCCCATCGAAGGTA CAGTACTGTGATAACTGAAGGATGGCTGCC	Ice_Fa_000733F:206957-207035	+
cac-mir-101b	CTGTCCATTTTCAGTTATCATGGTACCGGTGCTGTGTCCCTCTTAAGTAC AGTACTATGATAACTGAAGATTGGCAGTG	LG12:15446173-15446251	+
cac-mir-103a	CTTTGCGTTCAGCCTCTTTACGGTGCTGCCTTGTGACGTCTTGATCAAGC AGCATTGTACAGGGCTATGAAGGCGTAGAG	LG22:23549660-23549739	+

cac-mir-103b	CTACGCTTTTAGCCTCTTTACAGTGCTGCCTTGTCTGATCATGTTCAAGC AGCATTGTACAGGGCTATGACAGCATAGAG	LG10:28275928-28276007	-
cac-mir-107a	GTGTGCTGTGAGCTTCTTTACAGTGTTGCCTTGTGGCATGGGGATCAAG CAGCATTGTACAGGGCTATCACTGCACAC	LG15:28318936-28319013	-
cac-mir-10a	CACCTATATATACCCTGTAGATCCGAATTTGTGTAAAAATAGCAGCAGC CACAAATTCGCTTCTTGGGGAGTATGTAGGTGA	LG19:6743208-6743289	-
cac-mir-10b-1	CGTCTATATATACCCTGTAGAACCGAATTTGTGTGATAAAACCACAATC ACAGATTCGATTCTAGGGGAGTATATGGTCGA	LG21:18076974-18077054	-
cac-mir-10b-2	TGTCTATATGTACCCTGTAGAACCGAATTTGTGTGAAGTACAAACAATC GCAAATACGTCTCTACAGGAATATATGGGCAA	LG7:22098995-22099075	+
cac-mir-10c	CTTCTATATCTACCCTGTAGATCCGGATTTGTGTACAAATCATTGAAAC AATCACAAATTCGCTTCTAGGGGAGTATATAGTG	LG8:1699781-1699863	+
cac-mir-10d	CGCCCCTACATACCCTGTAGAACCGAATGTGTGCGGAGCTGCTTCAGAC ACAGATTGGGTTCTAGGGGAGTCTATGGGCGATGACTAACCA	Ice_Fa_000968F:130223-130313	-
cac-mir-1-1	TGCTTGGGGGACATACTTCTGTATATGCCCATATGAACAAGAGCAACTA TGGAATGTAAAGAAGTATGTATCCCAGGTTGG	Ice_Fa_000302F:824290-824370	-
cac-mir-1-2	CTCCTTGGTGTACATACTTCTTTATGTACCCATATGAACATATGATAGCT ATGGAATGTAAAGAAGTATGTATTCTTAGTGGG	LG17:22231114-22231196	-
cac-mir-122	TCCAGAGCTGTGGAGTGTGACAATGGTGTTTGTGTCCTGTGAATCAAAC GCCATTATCACACTAAATAGCTCCAGTGTGAAA	LG12:8779683-8779764	+
cac-mir-124-1	TTGAGCTCTTTGTGTTCACAGTGGACCTTGATTTAATTTCAATACAATTA AGGCACGCGGTGAATGCCAAGAGAGAAGCC	Ice_Fa_000169F:967395-967474	+
cac-mir-124-2	TGTGTCTCTCCGTGTTCACAGCGGACCTTGATTTAATGTCTTACAATTAA GGCACGCGGTGAATGCCAAGAGATGAGCC	LG20:18871716-18871794	+
cac-mir-124-3	TGTCACTCTGCGTGTTCACAGCGGACCTTGATTTAATGTCCATACAATT AAGGCACGCGGTGAATGCCAAGAGAGGAATC	LG7:26145867-26145946	-
cac-mir-124-5	CGTCTCTCCTCGTGTTCACAGCGGACCTTGATTTAAATGTCCATACAATT AAGGCACGCGGTGAATGCCAAGAGAGTGACC	LG24:30332422-30332502	-
cac-mir-125a-1	TGCCCCTCTGTCCCTGAGACCCTTAACCTGTGAGGTCAAAGTAGGTCAC AGGTGAGGTCCTCGGGAACAGGGCTGCAT	LG16:19230756-19230833	+
cac-mir-125a-2	TGCTTCATTGTCCCTGAGACCCTTAACCTGTGATGATGTGAAAGGTCAC AGGTGAGGTCCTTGGGAACAAAGTCGCAT	Ice_Fa_000667F:204664-204741	-
cac-mir-125b-2	CCTCTCTCATTCCCTGAGACCCTAACTTGTGACGTTTTCGTCATGTGCAC GGGTTGGGTTCTCGGGAGCTGCGAGGGGCG	LG14:29502955-29503034	-
cac-mir-125b-3	CTTCACCTGCTCCCTGAGACCCTAACTTGTGAGCTCTTTTAATAAAAAA CCACGGGTTAGGCTCTTGGGACGTGGGCGGAGG	LG14:5192530-5192611	+
cac-mir-125c	TCTCTCTCCGTCCCTGAGACCCTAACTTGTGACGTTGTGTTTTCATGTCC ACGGGTTAGGCTCTCGGGAGCTGAGGGGGAT	LG13:9481803-9481883	+
cac-mir-126b	CCTCGCGGTCCATTATTACTTTTGGTACGCGCTATGCCACTCTCAACTCG TACCGTGAGTAATAATGCACTGCGACTGG	LG12:3547849-3547927	+
cac-mir-128-1	AGGCGGGATTCGGGGCCGGGACGCTGTCTGAGAGACCTCTATGAATCT CACAGTGAACCGGTCTCTTTTGCAGCCTCT	Ice_Fa_000589F:76688-76765	+
cac-mir-128-2	CAGTAGAGGAGGGGGCCGTTACACTGTCAGAGATGTAGTCTGAGGGTC TCACAGTGAACCGGTCTCTTTTCCTGCTGCT	LG11:10319945-10320023	+
cac-mir-128-3	AGGCGGGATTCGGGGCCGGGACGCTGTCTGAGAGACCTCTATGAATCT CACAGTGAACCGGTCTCTTTTGCAGCCTCT	LG2:10617292-10617369	+
cac-mir-129-1a	CTTCACGAATCTTTTTGCGGTCTGGGCTTGCTGTTCATAACTATTAATCT GGGAAGCCCTTACCCCAAAAAGCATTTGCGGAGGG	LG6:34110770-34110854	-
cac-mir-129-1b	CGTCACGAATCTTTTTGCGGTCTGGGCTTGCTGTGCATACATGAATCTG GGAAGCCCTTACCCCAAAAAGCATTTGTGGAGGA	LG3:18585819-18585901	-
cac-mir-129-2a	CTTTTCAGGTCTTTTTGCGGTCTGGGCTTGCTGTCAAATGACTATCCAGG AAGCCCTTACCCCAAAAAGCATCTACAAGGGA	LG6:20318027-20318108	-
cac-mir-129-2b	CTGTGTGGGTCTTTTTGCGGTCTGGGCTTGCTGTTCCTAAGGCAGTAGC CAGGAAGCCCTTACCCCAAAAAGTATCTGCACTGGACTCTGG	LG23:6689968-6690058	+

cac-mir-130b	TCTGCTTGACACTCTTTCCCTGTTGCACTACTGTGGGAGCTGCAGCAAGCAGTGCAATAATGAAAGGGCATCGGTCAGCT	LG9:1598929-1599008	-
cac-mir-130c-1	GTTGTCCAAAGCCTTTTTTCTGTTGTACTACTGTGAAATCAGATGAGCAGTGCAATATTAAAAGGGCATTGGCTGACA	LG9:1604812-1604889	-
cac-mir-130c-2	GGAGTCTGATGGTGTCCATGTTGTTGTCCACTGCCCTTTTTTTTGGTGCACTACTGGACATTGAGGTGAGCAGTGCAATATTAAAAGGGCATTGGCTGATG	LG12:14133244-14133344	-
cac-mir-132-1	CTCCATGGCGACCGTGGCATTAGATTGTTACTGTAGCAACAGCACCACTGGTAACAGTCTACAGCCATGGTCGCTAGGGGGCA	LG13:18746562-18746644	-
cac-mir-132-2	CTCCATGGCGACCGTGGCTTTAGATTGTTACTCTAGCAACAGCACCATGGTAACAGTCTACAGCCATGGTCGTTAGGGGCAG	LG14:22162712-22162793	+
cac-mir-133a-1	TTGCTAAAGCTGGTAAAATGGAACCAAATCACCTCTTGAATGGATTTGGTCCCCTTCAACCAGCTGTAGCTATGCTT	LG17:22227837-22227913	-
cac-mir-133a-2	TTGCTAAAGCTGGTAAAATGGAACCAAATCAACCGTTCAATGGATTTGGTCCCCTTCAACCAGCTGTAGCTGTGCAT	Ice_Fa_000302F:800380-800456	-
cac-mir-133b	CCTTGCTGTGGCTGGTCAAACGGAACCAAGTCAGGTGTTTCTGTGAGGTTTGGTCCCCTTCAACCAGCTACTGCGTCGTG	Ice_Fa_000312F:266116-266195	+
cac-mir-135a-1	CGTTGTGTCTTATGGCTTTTTATTCCTACGTGATGGCCGATGGGTTCATGTAGGAGTAGAAGCCACTAAACACGCGG	Ice_Fa_001615F:35347-35423	+
cac-mir-135a-2	CAACGTGTACTATGGCTTTTTATTCCTATGTGATGATGAAACATGTTCGTGTAGGGATAGAAGCCATTTTACACG	LG6:22856818-22856892	+
cac-mir-135b	TAGTGCGCTGTATGGCTTTTTATTCCTATCTGACTGTACTGATGGTTCATATAGGGATGGAAGCCATGCACCGCGCTGGG	LG7:15956568-15956647	-
cac-mir-135c-1	GCTGTGTTTTTATGGCTTTCTATTCCTATGTGATTTTTCTCTGGCATGTCACATAGGGTCTTAAGCCATTGGGTACAGAGCGGGAGATCTGCAGACGGGGA	LG5:39674953-39675053	+
cac-mir-135c-2	AACTGTGTCTTATGGCTTTCTATTCCTATGTGAGTTTGTTCGAACATTTCATGTAGGGTTCAAAGCCATTGGATACACACTGGATAGTCAC	LG7:30997971-30998061	+
cac-mir-137-1	AGGTGGAACGACCGTTTGCTCCCGTCTCGACCACGGGTATTCTTGGGTTGATAATACAGATGTCGATGTTATTGCTTGAGAATACGCGTAGCTGAGCGGAA	LG8:29677814-29677914	-
cac-mir-137-2	GGAGGTGGGCCTCTTTTGCTCCGGTCTCGACCACGGGTATTCTTGGGTTGATAATACAGATGTGGATGTTATTGCTTGAGAATACGCGTAGTCGAGTGGAA	LG1:23865642-23865742	-
cac-mir-138	GGGGAGGGTCAGCTGGTGTTGTGAATCAGGCCGCTGACGCGTCACACACGGCTTCTTCACAACCCCAGGGACCCTCCCTACTACTTCCTGC	Ice_Fa_000630F:37231-37321	-
cac-mir-1388	TAGTTCTTCGAGGACTGTCCTACCTGAGAATGGTGATTTCAGGCTCAATCTCAGGTTCGTCAGCCCATGAAAAGCTAAC	LG7:25907325-25907403	+
cac-mir-138b	TGTGTGCTGCAGCTGGTGTTGTGAATCAGGCCGATGACAGACACCTCCTAAAACCCGGCTATTTCACAACACCAGGGTGGCACCGCACCAC	LG6:10428335-10428425	-
cac-mir-138bb	GGGGCGGGACAGCTGGTGTTGTGAATCAGGCCGCCGAAATCCAAGGACCGGCTACTTCCCAACACCAGGGTCCCACCCTCACCCCTCACAT	LG11:20343644-20343734	-
cac-mir-139	TTGGCTGTATTCTACAGTGCATGTGTCTCCAGTGTTAGTGATGCTACTGGAGACCAAGCTCTGTTGGAATAACAATCA	LG13:30614625-30614702	+
cac-mir-140	AGTGTCACGTCAGTGGTTTTACCCTATGGTAGGTGACATCATGCTGTTCTACCACAGGGTAGAACCACGGACGGGATGTTG	LG7:4303492-4303572	+
cac-mir-142a	CAGTCATCCATAAAGTAGAAAGCACTACTAAACTCCTCGCCACAGTGTAGTGTTTCCTACTTTATGGATGAGTGT	LG14:11530310-11530384	+
cac-mir-142b	TTGTCACCCATAAAGTAGAAAGCACTACTAAACGTCATTACACAGTGTAGTGTTTCCTACTTTATGGATGAGTAT	LG13:17534039-17534113	-
cac-mir-143	CCAATCCAGACAGATGAGGTGAGGGTCAGAGTTTGAGAAGGGGGGAGGGGGGCGTGTTCAGATCTATGGTTCAGATGAAGCTCTGTAGCTCAAGGCAGTTT	LG24:26719997-26720097	-
cac-mir-144	TGCCCCGGACAGGATATCATCTTATACTGTAAGTTTAATAAAGAGACACTACAGTATAGATGATGTACTATCCAGGGT	LG14:11099725-11099802	-

cac-mir-145	CTTCCATTGTGGCCAGTTTTCACAGGAATCAGGAAGTCAATGTTTATAGTGATAATATCTATTACATCTTATATTTGTTGTGTGAGTCTTAGGCTGACAGA	LG17:32176763-32176863	-
cac-mir-146a	TGCTGTACTATGAGAACTGAATTCCATAGATGGTGGTGTGTTCAGGTGTCATCTATGGGCTTAGTTCTTTTGGAATGGTACT	LG15:18765471-18765552	+
cac-mir-148	TTTCCAAATAAAGTTCTGTGATACACTTAGACTCTAATTGCCTGCAGTCAGTGCATTACAGAACTTTGTTTTGTGAGT	LG21:17936178-17936255	-
cac-mir-152	TTCCTGGTCTAAGTTCTGTGATACACTCCGACTGTGAATCTATGCTAGTCAGTGCATAACAGAACTTTGTCCCGGCTCT	LG8:2567655-2567733	-
cac-mir-153a-1	CCTCCTCCCCCTCCTAACTAGCGGTTGCCAGTGTCATTTTTGTGATGTTGCAGCTAGTAATATGAGCCCAGTTGCATAGTCACAAAAGTGATCATTGGAGA	Ice_Fa_001706F:9962-10062	-
cac-mir-153a-2	CACTAAGCGCTTTTCTACCTCGTTCTTAGGTTCAGCTCTTTGGGAGAATTGTGTAACTCACACCCCAAACATTGCATAGTCACAAAAGTGACTTTGAACGG	LG22:23619626-23619726	+
cac-mir-153b	GCGGCGGGTTAAACTTGAACATATCTGTCTGTGTCATTCTTGTGGTTTGCAGCTAGTTTGTGTCTCCAGTTGCATAGTCACAAAAATGATCATGGACTGAT	Ice_Fa_003589F:21741-21841	-
cac-mir-153c	CCAGTGTCATTTTTGTGATTTGCAGCTAGTACTCTGGCTCCAGTTGCATAGTCACAAAAATGAGCATTGGCAGGT	LG21:26060585-26060659	-
cac-mir-155	CATGGTGAGGTTAATGCTAATCGTGATAGGGGTTGTTTATTACCAGACACCTAACATGTTAGCATTAGCTTCACTCTGGG	LG3:26468235-26468314	+
cac-mir-15a-2	GGCGATGCTGTAGCAGCACGGAATGGTTTGTGGGTTACACAGAGATGCAGGCCATGCTGTGCTGCCGCAGAAGAGCCT	LG21:28574032-28574109	+
cac-mir-15b	GGGTGCGCTCTAGCAGCACATCATGTTTTGCAGATATTTGCTGAATCACTCCAAATCATTTTGTGCTGCCACCGTGAGCCCTG	LG18:1184741-1184823	-
cac-mir-15c	TAGACTGCTATAGCAGCGCATCATGGTTTGAAACAGTGTGGAAAAGGTGCGAACCATTATTTGCTGCTTTAGAATTTT	LG13:22636013-22636090	+
cac-mir-16a	TGCCACGCTTTAGCAGCACGTAAATATTGGCGTGTGAGACCCCAACCCCAATATTGGCAGTGCTGCTTCAGTGTGGCCGG	LG13:22636252-22636331	+
cac-mir-16c	CGCCTTACTGTAGCAGCACGTAAATATTGGAGTTAAGACTTTAGCTGAAGCCTCCAGTATTGATCGTGCTGCTGAAGCAAAGCTG	LG21:28574235-28574319	+
cac-mir-1788	ATACAGTCCAGCTTTCGAGGCTTGTTTTAAGTTGCCTGCGACTCTTACTTGGACACAGGCAGCTAAAGCAAGTCTGGGATGCAGAGACA	LG21:12236854-12236942	+
cac-mir-17a-1	TGTGTATTGTCAAAGTGCTTACAGTGCAGGTAGTTCTATGTCATACCTACTGCAATGGAGGCACTTACAGCAATACCCTGA	LG3:26485595-26485675	+
cac-mir-17a-2	ACTGTAGTGTCAAAGTGCTTACAGTGCAGGTAGTTTTATAAGATCTACTGCAGTGAAGGCACTTTCAGCACTATTCTGA	LG21:17070943-17071021	+
cac-mir-17a-3	AGTTTGTGATAAAGTGCTTACAGTGCAGGTAGTGATTATAACCTGGCCTACTGCAATGTGAGCACTTCTTTCCCACGGCG	LG10:28714426-28714505	-
cac-mir-181a-1	TGCCCCAGTGAACATTCAACGCTGTCGGTGAGTTTCAACGAAATTGAAAACCATCGACCGTTGATTGTACCCTGTGGCCAG	LG4:11780372-11780452	+
cac-mir-181a-2	GCTCACGGAGAACATTCAACGCTGTCGGTGAGTTTGAGCTCTGACCAAACCATCGACCGTTGCATGTACCCTGAGGGACG	LG9:10820887-10820966	+
cac-mir-181a-3	TGCCTCGGTGAACATTCAACGCTGTCGGTGAGTTTTGATATGGATCACATAAAAACCATCGACCGTTGACTGTGCCCCGGGGCTTG	LG17:15578636-15578721	+
cac-mir-181b-1	GGTCACAATCAACATTCATTGCTGTCGCTGGGTTGGACTGTGTAGAAAGCTCACTGAACAATGAGTGCAACTGTGGCCCAGATCTGCCCGA	LG17:15578849-15578939	+
cac-mir-181b-2	GGCTGCAATAAACATTCATTGCTGTCGGTGGGTTTACTTGAGAACAACTCACTGATCAATGAATGCAGACTGCGGTTCA	LG9:10821192-10821270	+
cac-mir-181b-3	GGTCACAATTAACATTCATTGCTGTCGGTGGGTTTAACTATGTAGAAAAGCTCGCTGAACGATGAATGCAACTGTGTCCC	LG4:11780572-11780651	+
cac-mir-182-1	TCTGGTGGTGTTTGGCAATGGTAGAACTCACACTGGTGAGGTAGATGGATCCGGTGGTTCTAGACTTGCCAACTACTACCTGAG	LG23:4351801-4351884	+
cac-mir-182-2	GACGGCTGCACTCTCCCACAGTGTTTGGCAATGGTAGAACTCACTCTGGTGGGCTGGAAGGATCCGGTGGTTCTAGACTTGCCAACAACTGACTGAGAGCTTTGACCTGCC	LG6:18739153-18739263	+

cac-mir-183-1	TGTTCTGTGTATGGCACTGGTAGAATTCACTGTGAGCGCTCACTATCAGTGAATTACCATAGGGCCATAAACAGAGCAGAGAAAGAACCAC	LG23:4350972-4351062	+
cac-mir-183-2	TGTTCTGTGTATGGCACTGGTAGAATTCACTGTCACGGCACACAATCAGTGAATTACCATAGGGCCATAAACAGAGTAGAGACTGAACCAC	LG6:18738739-18738829	+
cac-mir-184-1	TTCTGTGGTCGCTCACATCTCCTTATCACTTTTCCAGCCCAGCTATAGATTTTGTATCCGTTGGACGGAGAACTGATAAGGGCATGTGCAT	LG3:37654036-37654126	+
cac-mir-184-2	TAATGTTGTCGAGCACATCTCCTTATCACTTTTCCAGCCCAGCTATCTATTCAATATGTGTTGGACGGAGAACTGATAAGGGTATGTGTCC	LG6:23917881-23917971	-
cac-mir-187-2	CTGGGCCAGGGCTGCAACACAGGACATGGGTCCCGCTTGTCCTCCCCGCTCGTGTCTTGTGTTGCAGCCAGTGGTCTCGC	LG11:10758791-10758870	+
cac-mir-18a	AGTCCTTTGCTAAGGTGCATCTAGTGCAGATAGTGAAGTAGACTAGCACCTACTGCCCTAAGTGCTCCTTCTGGCATAA	LG21:17071379-17071457	+
cac-mir-18b	GTCTTTGTGCTAAGGTGCATCTAGTGCAGATAGTGAAATAGACTAGCACCTACTGCCCTAAGTGCTCCTTCTGGCATAA	LG3:26485722-26485800	+
cac-mir-18c	GTCCTCCAGCTAAGGTGCATCTAGTGTAGTTAGTGAAATAATTCAGAATCTACTGCCCTAGTTGCTCCTTCTGGCTGGAGGGCTTGTTGTT	LG10:4337230-4337320	+
cac-mir-1905-1	AACGATGTACGTGCAGGTTTGCTGCTGGATGCGTTTGATGGTGGAGCCTTTAGGCCCGACTACCAGCCCCACCACGCGGTATGGCACTCTCACCTGAATGGT	LG16:24403958-24404059	-
cac-mir-1905-2	CACGATGTAGGTGTGGGTCTGCTGCTGGATGCGCTTGATGGTGGCCCCTTTGGGACCTACAACGAGCCCCACCACACGGTAAGGCACCCGCACCTGGATGGT	LG3:5431636-5431737	-
cac-mir-1905-3	CACGATGTAGGTGTGAGTCTGCTGCTGGATGCGCTTGATAGTCGCTCCCTTTGGTCCAACAACTAAACCAACT	LG17:14689748-14689820	+
cac-mir-190a-1	GGGCTCTGTCTGATATGTTTGATATATTAGGTTGTTATTCAGTCCCAACTATATATCAAACATATTCCTACAGTGTCCCG	LG3:37443038-37443117	+
cac-mir-190a-2	GGGCTCTGTCTGATATGTTTGATATATTAGGTTGTTATTCAGTCCAACTATATATCAAACATATTCCTACAGTGTCCC	LG6:24299041-24299118	-
cac-mir-190b	AGAAGCTGTGTGATATGTTTGATATTCGGTTGTTCTTGTGCTTCATCGTGTCAACTAAATATCAGACATATTCCTACAGAGTCTGGCAAAA	LG16:21893141-21893231	+
cac-mir-192	ACACAAGGGGATGACCTATGAATTGACAGCCAGTGATCGTAACATTTGCCTGTCAGTTCTGTAGGCCACTGCTGTGTTGTCCATCTGACTG	LG14:9134974-9135064	-
cac-mir-193a	AGTTAGATGCTGGGTCTTTGCGGGCAAGGTGAGTCCTCACTTCGTTCAACTGGCCTACAAAGTCCCAGTTTCTGGCT	LG1:24202776-24202852	-
cac-mir-194a	GCTCGCTGGATGTAACAGCAACTCCATGTGGAAGCTGTGAGTGTTCCAGTGGAGGTGCTGTTACCTGCAGAG	LG14:9135142-9135213	-
cac-mir-194b	GCCTTGTCTGTGGGTCTCACCAGCTGTAACAGCATCTCCATATGGAACAACTCTGGCTTCCAGTGGAGCTGCTGTTATCTGTGGTGGGACACCTCTTG	LG10:23391190-23391287	-
cac-mir-196a-1	TCGAGTGGTTTAGGTAGTTTCATGTTGTTGGGGTCCATTTCTAACTCTGCAACATGAAACTGTCTTAATTGCCCCAG	LG11:19356718-19356794	-
cac-mir-196a-2	GAAGCGTGGTTAGGTAGTTTCATGTTGTTGGGGTTGGCTTCCTGGCTCGGCAACAAGAAACTGCCTTGATTACGTCAGTTCGTCTTCATCAAGGGCACAAT	LG7:22062661-22062761	+
cac-mir-196b	CTGTGTGATGTAGGTAGTTTCAAGTTGTTTGGGCTGGATGTTTAAGAACACAGGAACCTGAACTGCCCTGAATCACACCGACCTCAACTGCACAGAGGAAA	LG8:18332982-18333082	-
cac-mir-196d	TTGAGTGGTTTAGGTAGTCTCATGTTGTTGGGCTTTTATATTTCTCCCACAACACGAAACTGTCTTGATTACCACAGT	LG16:17946285-17946362	-
cac-mir-199-1	AGCCCGCCTGCCCAGTGTTCAGACTACCTGTTCAGGAAGTAGTGGTTGTACAGTAGTCTGCACATTGGTTAGGCTGGCCGG	LG12:11666948-11667028	-
cac-mir-199-2	CCCCCGCCTGCCCAGTGTTCAGACTACCTGTTCATCAGGTTACAGCTGAACAGTAGTCTGCACATTGGTTAGGCTGGGCTG	LG8:19542342-19542422	+
cac-mir-199-3a	GCTCCGTCTACCCAGTGTTCAGACTACCTGTTCATTGTCATACTGGTGTACAGTAGTCTGCACATTGGTTAGACTGGGCAT	LG17:6603714-6603794	-
cac-mir-199-3b	GCTCCGTCATCCCAGTGTTCAGACTACCTGTTCAGGATCTTACTGGTGTACAGTAGTCTGCACATTGGTTAGACTGTGCAA	LG4:29844093-29844173	+

cac-mir-19a-1	GTTCTCTGCTAGTTTTGCATAGTTGCACTACAAGAATAGATGAGTTGTG CAAATCTATGCAAAACTGATGGTGGCCT	LG3:26485865-26485941	+
cac-mir-19a-2	GTTCTCTATTAGTTTTGCATAGTTGTACTGCAAGAAGAATTGAGTTGTG CAAATCTATGCAAAACTGATGGTGGCCT	LG21:17071527-17071603	+
cac-mir-19b-1	TCACTTGGTCAGTTTTGCTGGTTTGCATCCAGCTTTTTCTGATGTTCGCT GTGCAAATCCATGCAAAACTGACTAGGTTGA	LG3:26486210-26486290	+
cac-mir-19b-2	GTCTCTGGTTAGTTTTGCTGGTTTGCTTTCAGCTTATCACTGTACTGCTG TGCAAATCCATGCAAAACTGATCATAGAAC	LG21:17071813-17071892	+
cac-mir-19c	GTTTACAGCCAGTTTTGTTGGTTTGCTCTCAGCTGTTGCCAGTCTCTGCT GTGCAAATCCATGCAAAGCTCTCTGTGTCTCA	LG10:4338682-4338763	+
cac-mir-19d	GATCCTGGCCAGCTTTGCAGGGTCGGCAGTCAGCCTGTGTACTCTAGGT GCCGCTGTGCAAACCCATGCAAAACTGACCATGGCCA	LG10:28713961-28714046	-
cac-mir-200a	TCTCAGGATCCATCTTACCTTACAGTGCTGGATTGTACTTATGTGGTTCT AACACTGTCTGGTAACGATGTTTCCTGGGT	LG7:13021182-13021261	+
cac-mir-200b	TGATTATCTCCATCTTACGAGGCAGCATTGGATACTCATCACTCTCTCTA ATACTGCCTGGTAATGATGATGATAGTCAT	LG7:13020998-13021077	+
cac-mir-202	CTGTTCCTTTTTCCTATGCATATACCTCTTTCAGATGTCACTTTAAAGAG GCATAGGGCATGGGAAAATGGGGCTGCAGAGGTTTTCCGCC	Ice_Fa_000412F:424132-424222	+
cac-mir-203b	CCTCTGATTAAGTGGTTCTCAACAGTTCAACAGTTCTTTACAAAAATTG TGAAATGTTTAGGACCACTTGACCAGTCAGA	LG22:16832204-16832283	+
cac-mir-204-1	GCCCGTGGGCTTCCCTTTGTCATCCTATGCCTGGAGCTCGGTTAAGGCA GGGACAGCAAAGGGATGCTCAAATGTCACCAAAGACTTCACT	LG6:24937925-24938015	+
cac-mir-204-2	ACCAGTGGGTTTCCCTTTGTCATCCTATGCCTTGAGCTGTGTAAGGCAG GGACAGCAAAGGGAGGCCCAGCCGTCACTACCTTCAGCTTTA	Ice_Fa_002768F:3032-3122	+
cac-mir-204-3	ACCTATGGACTTCCCTTTGTCATCCTATGCCTGGACTCATACAAAAGGG GCTGGGAAGGCAAAGGGACGGCCAGTCGTCACACAAGCGTCT	LG9:36657568-36657658	+
cac-mir-205-1	ATGTGTTCTATCCTTCATTCCACCGGAGTCTGTATCTGCATCCAACCAG ATTTCAGTGGTGTGAAGTGTAAGAGACATGGGG	LG7:38173290-38173371	+
cac-mir-205-2	ATGTATTCTATCCTTCATTCCACCGGAGTCTGTGTAAAGTGCCAATCAG ATTTCTGTGGTTTGAAGAGTAAAACACATG	LG5:12875409-12875487	-
cac-mir-206-1	CTTGTGAGGACATGCTTCCTTATATCGCCATATTAATTCACCACTTATGG AATGTAAGGAAGTGTGTGGTTTCAATG	Ice_Fa_000312F:265355-265431	+
cac-mir-20a-1	TCGGTAGTATTAAAGTGCTTATAGTGCAGGTAGTTGTATGCATTTCTAC TGCAGTGTGAGCACTTGCAGTACTCCTA	LG21:17071688-17071764	+
cac-mir-20a-2	TCAGCAGTGCTAAAGTGCTTATAGTGCAGGTAGTGTCTTTCTCCTATCT ACTGCAATGTAAGCACTTAAAGTACTTCTAAC	LG3:26486074-26486154	+
cac-mir-20b	CTGGTAGTCCCAAAGTGCTCACAGTGCAGGTAGTACTCACTGACCTACT GCAGTTTGTGCACTTCAGGTGCTGCCGGTCACCTTCCCTCAC	LG10:4338559-4338649	+
cac-mir-210	AAAAGCAGGTAAGCCACTGACTAACGCACATTGTGCCAGTTGACAATT CCACTGTGCGTGTGACAGCGGCTAACCTGGTTTT	LG6:18152801-18152882	+
cac-mir-21-1	GCCTGTCAGATAGCTTATCAGACTGGTGTTGGCTGTTAACGTTGCAAGG CGACAACAGTCTGTAGGCTGTCTGACATTTC	LG14:29216399-29216478	-
cac-mir-21-2	CTGCGTCAGCTAGCTTATCAGACTGGTGTTGGCTGTTATATTTAAAAGG CAACATTGGTTTGTAAGCTGGCTGAAGTCTG	LG13:35444789-35444868	+
cac-mir-212-1	GTGCATCAATACCTTGGCTCTAGACTGCTTACTGCTAAAACTCCTCCAA AGTACAGTAACAGTCTACAGTCATGGCTACTGACGT	LG13:18746823-18746907	-
cac-mir-212b	GAGCATCAGCACCTTGGCTTTAGACTGCTTACTGCTTATACAGCGTTGC AGTACAGTAACAGTCTACAGTCATGGCTACTGAAGC	LG14:22162343-22162427	+
cac-mir-214a	GCAGTGTGTCTGCCTATCTACACTTGCTGTGCAGAATATCCTCCAACCT GTACAGCAGGCACAGACAGGCAGATAGACA	LG17:6602196-6602274	-
cac-mir-214b	GTGATGTGTCTGCCTGTCTACACTTGCTGTGCAGACCTTCTGCTCCTGTA CAGCAGGCACAGACAGGCAGACAGATG	LG4:29846779-29846855	+
cac-mir-216a-1	TGTTTTGGTGAAATCTCAGCTGGCAACTGTGAGTCGTTCACTAGCTGCT CTCACAATGGCCTCTGGGATTATGCTAAACGCAG	LG15:20465579-20465661	+

cac-mir-216a-3	TGGTCCAGTTTAATCTCAGCTGGCAACTGTGAGCTTTGAACGCCTCACAGAGCCGGCTAGGGTTCTCCTGAACGCAGCACCATCATTTCCT	LG1:36596133-36596223	-
cac-mir-216b-1	GAGGACTGGGTAATCTCTGCAGGCAACTGTGATGGTGCTTTATATTCTCACAATCACCTGGAGAGATTCTGCAGTTTAGTC	LG15:20464861-20464941	+
cac-mir-216b-2	GTCGCCTTGTTAATCTCTGCAGGCAACTGTGATGTCACGTTTTCTCACAATCACCTGCAAAGATGATCCAGTCGCTCTGCTCAGACCAGCA	LG1:36596298-36596388	-
cac-mir-217a	ATGTGGATGATACTGCATCAGGAACTGATTGGCTGATGCTCAATAGCCAACAGAACCTGATGCATTGCCTTCAGCATCA	LG15:20466090-20466168	+
cac-mir-217b	ATGTTGAAGATACTGCATCAGGAACTGATTGGAGAGCGATGAGGCCATCAGCTCCTGATGCAATGCCTTCAGCATTAAAAGACACTTCCCC	LG1:36594737-36594827	-
cac-mir-2184	CTCAGCCCTGAACAGTAAGAGTTTATGTGCTGTTGTTAATCTGTCAGCACATGAGCTTTTACGGTGCAGGGAAGCAGACGACCAACATCTT	LG14:22164003-22164093	-
cac-mir-2187a	GGAAGATCTGATTGATGATTCTGGCTTTAATTAGTATAGCCTGTTTTAGTGATATCAGCAATTCTTTACAGGCTATGCTAATCTGTGCCAGAATTAGCAATGCAATTGGAACGGACGCA	LG15:25192894-25193012	-
cac-mir-2187b	TGAGGAGATGTGATGGTTCTGGCTTTAATTAGTACAGCCTGTATTAGTAATGTCATCTATTCTTTACAGGCTATGCTAATCTATGCCAGAACCAGCAATGCAATGGGAGACAGAAC	LG19:1662919-1663034	+
cac-mir-2188	TGGTGTGGGAAAGGTCCAACCTCACATGTCCTGTGCGGCTGAAGGAAGGCTGTGTGAGGTCAGACCTATCCCACACGGCT	LG9:35226710-35226789	-
cac-mir-218a-1	CAGCTGTCTCTTGTGCTTGATCTAACCATGTGGCCCCGCCTACTGTGGCATCACATGGTTCCGTCAAGCGCCAGGGACCGCCGAGCACACT	LG10:29435375-29435465	+
cac-mir-218a-2	GGTTGTTCCTTTGTGCTTGATCTAACCATGTGGCTGCAAGGTTCCTAAAGTGGAACATGGTTCCGTCAAGCACCATGGAACGGC	LG1:21010467-21010550	+
cac-mir-218b	CAGGACCCCATTGTGCTTGATCTAACCATGCAGTGTATCTTCTGTCCATGGTTCTGCCAAGCACCTTGGAGGCTTGTGAGCACTCTCGGCA	LG12:14063738-14063828	-
cac-mir-219-1	CTCTAGCTGCTGATTGTCCAAACGCAATTCTTGTTACATTTTAATCCAACCCGAGAATTGTGCATGGACATCTGTTGCTTGACGCTCGTGC	LG9:3032198-3032288	-
cac-mir-219-2	AGTCTGGCGTTGATTGTCCAAACGCAATTCTTGTGTTGATTCTCCATATCCAGGAGTTGTGCATGGACATCTTGCCTCAGACTCTCATTTA	LG16:18536377-18536467	+
cac-mir-219-3	CTTTAGCGACTGATTGTCCAAACGCAATTCTTGAGTAAACTCAAATTCAACCCCAAGAATTGTGTATGGACATCTGTTGCTGCAGACTCAC	LG12:15027720-15027810	-
cac-mir-219-4	GGTCCGGAGCTGATTGTCCAAACGCAATTCTTGCGTCTGCCTTTGTGAAACCAGGAGTTGTGCATGGACATCACGCCCCTGACCTCTGAAA	LG11:7309399-7309489	+
cac-mir-2196	GTGCATGTCGGGGTAGTTGCTGCGGATGTTCCTCTCTGTGCTCCCGTTCGGGACCGTCCCAAAGCGAAAAGGAGGATAATGCTCATGTGGTTTCTGGAACTGTAGGAAGACA	LG19:11335126-11335237	-
cac-mir-221a	TTTGTCCTGAACCTGGCATACAATGTAGATTTCTGTGTGGTTTATTTCTACAGCTACATTGTCTGCTGGGTTTCTGGCTAGCA	Ice_Fa_003231F:26032-26114	-
cac-mir-221b	TGAGGTTTGAACCTGGCATACAGTGTAGGATGCTGTGTGTGTCAGTCTACAGCTACATTGTCTGCTGGGTTTCATACCTTTA	LG2:6896178-6896259	-
cac-mir-222a	TGCTGTCAGTTGCTCAGTAGGCAGTGTAGATCCTGTGTAGAAATCAGCAGCTACATCTGGCTACTGGGTCTCTGACGGCTCTG	Ice_Fa_003231F:26824-26906	-
cac-mir-222c	GCCCAGCAGTTGCTCAGTAGTCAGTGTAGATCCTGTGGGGCTGGCAGCAGCTACATCTGGCTACTGGGTCTCTGCTGGCATCC	LG2:6896411-6896493	-
cac-mir-223	CCCCACTTCGTGTATTTGACAAGCTGAGTTTGACACTCTTGTTTCGCTGAGTGTCAGTTTGTCAAATACCCCAAGTGAGGTGT	LG14:6538352-6538434	-
cac-mir-22a-1	GACCTACAGCAGTTCTTCACTGGCAAGCTTTATGTCCTCATGTACCAGCTAAAGCTGCCAGCTGAAGAACTGTTGTGGTCGGC	LG13:27228880-27228962	-
cac-mir-22a-2	GACACACAGCAGTTCTTCACTGGCAAGCTTTATGTTCCTGTACACATGCTAAAGCTGCCAGCTGAAGAACTGCTCTGGTCTG	LG14:21486247-21486328	-
cac-mir-22b	GCCTCACAGTCGTTCTTCACTGGCTAGCTTTATGTCCCACGCCCCACACTAAAGCTGCCAGTTGAAGAGCTGTTGTGTGC	LG9:34049271-34049350	+
cac-mir-23a-1	GTGGTGGGGAGGGTTCCTGGCACCGTGATTTGATGCACAAAGAGAAACAAAAATCACATTGCCAGGGATTTCCACTCCTTCACAG	LG4:39604012-39604096	-

cac-mir-23a-2	GATGGCCAGGGGAATTCCTGGTAGAGTGATTTTTGAGACTACAGGACTGAATCACATTGCCAGGGATTTCCAATGGCTGACA	LG8:22605266-22605347	-
cac-mir-23a-3	TTAGCTGGAGGGATTCCTGGCAGAGTGATTTGGTTGTGATGTCATGTAAATCACATTGCCAGGGATTTCCAACCAGCTACA	LG17:22723991-22724071	-
cac-mir-23a-4	CCCAGTGTGTGTGTGGTGATGAAACAGAAAAAGAGTCTGGTGCATATGGCAGCTATCTGGCTTAATTGCCAGGGATTTCCATTCCCATCGC	LG2:6958299-6958389	-
cac-mir-23b-1	GTGAGGGTCTCTGGCATGATGATTTGGGACAGAAAACGAAAATCACATTGCCAGGGATTACTACACTGCTG	LG12:23509140-23509210	+
cac-mir-23b-2	GAGACGGTCTTGTTCTTTCATGTCTGGGCTGTGAGGGTTCCTGGCGTGCTGATTTGTGATTTATGATAAAATCACATTGCCAGGGATTACCACAACCCTGA	LG9:19563022-19563122	+
cac-mir-24-1	GATTACTCCCAGGAAACATCTGGCCTCCCTCTGGTGCCTTCTGAGCTGAGCGCAGTTAGATCTCCACACACTGGCTCAGTTCAGCAGGAACCGGAGTCACG	LG12:23510684-23510784	+
cac-mir-24-2	TCAGTCTCCTGTGCCTGCTGTGCTGATAATCAGTGTGTGATGTCGGCTGGCTCAGTTCAGCAGGAACAGGAGACTGGT	LG8:22603570-22603647	-
cac-mir-24-3	TGGCTTCCTGTGCCTACTGAACTGATTAACAGTCCTACAGAACCACTGGCTCAGTTCAGCAGGAACAGGAGTCCAG	LG9:19569675-19569750	+
cac-mir-24-4a	TACCTTCCCGTGCCTACTGAACTGGTATCAGTGTTTTACCAAAAAAACTGGCTCAGTTTCAGCAGGACCAGGAGTGAAGTCCACTTTCAACCACATGTGAC	LG4:39600098-39600198	-
cac-mir-24-4b	TGTACTCTTGTGCCTACTGGACTGGATTCAGTGTGTCTCTGCAAAACTGGCTCAGTCCAGCAGGAACAAGAGTGACT	LG17:27201815-27201891	-
cac-mir-24-6	CAATCTCCTGTGCCTACTGAGCTGATAACAGTTTGATGTTAAACAAACACTGGCTCAGTTCAGCAGGAACCAGAGTTAGG	LG17:22722005-22722084	-
cac-mir-25	TGGTGTTGAGAGGCGGAGACTTGGGCAATTGCCGGCCATACCAGAGGGCATTGCACTTGTCTCGGTCTGACAGTGCCGGC	LG10:28713761-28713840	-
cac-mir-26a-1a	CTGGGTCTGTTTCAAGTAATCCAGGATAGGCTTGTTAAAGTGGGGAAAGCCTATACGGGATGACTTGGTTCAGAAACAAGG	LG21:10818639-10818719	+
cac-mir-26a-1b	CTGGGCCTGATTCAAGTAATCCAGGATAGGTTTGTTCAGACAAGCAAAGCCTATTCCGTATGACTTGATTCAGGAACGCTA	LG2:3487349-3487429	+
cac-mir-26a-2	TGTGATCCAGTTCAAGTAATCCAGGATAGGCTGTGTGTATCCTGATTGGCCTATTCATGATTACTTGCACTGGGTGGCAGC	LG17:9186821-9186901	+
cac-mir-26b	CTCTGCCTGGTTCAAGTAATCCAGGATAGGCTGGTTAACTCTGGCACGGCCTATTCTTGATTACCTGTTTCAGGAACTGGC	LG7:33407907-33407987	+
cac-mir-27a	ATGGAGGGGCAGGACTTAGCTTGCTCCGTGAACAGTGCAGTTGAAGCCTGTGTTCACAGTGGCTAAGTTCCGCTCCTCACAAG	LG17:22723484-22723566	-
cac-mir-27b	CAGCCAGGCACAGAGCTTAGCTGATTGGTGAACAGTGATTGATTTCCTCTTTGTTCACAGTGGCTAAGTTCTGCACCTGAGG	LG9:19563482-19563563	+
cac-mir-27c	TTGCGGCAGCAGGACTTAGCCCACATGTGAGCAGTGAGTGTCTGCCATGTTCACAGTGGTTAAGTTCTGCCGCCACAGGG	LG8:22605049-22605128	-
cac-mir-27d	CTGCTGTTTCTGATCAGGTGCAGAGCTTAGTCGACTGCTGGACAGGGAGTCTCAGCAATTTGTTCACAGTGGCTAAGTTCTTCACTTGTGC	LG12:23509392-23509482	+
cac-mir-27e-1	CTAAAGGCACAGAGCTTAGCTAATTGGTGAGCATTGATCCCTGCTATGTGTTGTTCACAGTGGCTAAGTTCAGTGCCTGAGGTG	LG4:39603464-39603547	-
cac-mir-27e-2b	TTTCTGAAGATGCAGAACTTAGCTCATTAGTGAGCATTGAAACAAACAAGAATTGTTCACAGTGGCTAAGTTCAGTGTCTGACATG	LG17:27202143-27202228	-
cac-mir-2985-1a	GCTACGCCGAAAGGATCCTCATTAAGGTGGGTGGAATAGTATAACAATGTGTCCAATGTTGTTATAGTATCCCACCTACCCTGATGTAGCTTTGCTTATTT	LG10:30161300-30161400	+
cac-mir-2985-2b	AAGGCTCACAGTTAAGGTGGGTGGAATAGTGTAACAATAGAAGACAGTAACAAAGCTGATACTGTCCTATTGCTGCAATATTCCACCTACCCTGCTGTGCCTTTTATACTTC	LG13:29395312-29395423	-
cac-mir-2985a	GCGACGCCCAAAGGCTCAGCATTAGGGTGGGTGGAATAATATAACAATATCCTCCCTGTTGTTATAGTATTCCACCTACCCTGATGTCCCCTTGTTT	LG10:11512999-11513095	-
cac-mir-2985b	ACCTAAAGGCTCTGCATTAGGGTGGGTGGGATAATATAACAATATCCTCCATGTTGTTATAGTATTCCACCTACCCTGATGTTTTCTTTTGCCA	LG14:28054216-28054309	-

cac-mir-29a-1	TCTCCAGGGAACTGGTTTCGGATGGTGTTTTAGAGTGTAACATATGATCTAGCACCATTTGAAATCGGTGTTCTTGAG	LG5:13035688-13035765	+
cac-mir-29a-3a	CCCTTGGAAGCTGGTTTCATGTGGTGGCTTAGAGTCTCATCATCTATCTAGCACCATTTGAAATCGGTGGTCTTGGG	LG7:38610704-38610780	-
cac-mir-29a-3b	CTAAAAGATGACTGATTTCTCCTGGTGTTTAGAGCCCGCTGCAGCCTTCTAGCACCATTAGAAATCGGTTATAAAACTGTG	LG23:19621638-19621718	+
cac-mir-29b-1a	CCCCCAGAAGCTGGTTTCCCATGGTGGATTAGATGTTCCCATTGTCTAGCACCATTTGAAATCAGTGTTCTTGGCGA	LG23:19621492-19621568	+
cac-mir-29b-1b-1	CCCTTGGAAGCTGGTTTCATGTGGTGGCTTAGAGTCTCATCATATATCTAGCACCATTTGAAATCAGTGGTCTTGGGGT	LG7:38604864-38604942	-
cac-mir-29b-1b-2	CCCTTGGAAGCTGGTTTCATGTGGTGGCTTAGAGTCTCATCATATATCTAGCACCATTTGAAATCAGTGGGTCTTGGGG	LG7:38608321-38608399	-
cac-mir-29b-2	ACCAAGGCTGACCGTTTTCGTCTGGTGTTCAGAGTTTCCATCATGTCTAGCACCATTTGAAATCGGTTACAATGTA	LG5:13036020-13036095	+
cac-mir-29b-4	CACTCAGAAACTGATTTCATTTGGTGACGTAGATGTTTGTACCCAGTCTCTAGCACCATATGAAATCAGAGTTCTGGGAGGT	LG6:28927436-28927517	-
cac-mir-301a	GTTGGCAGGTGCTCTGACTTCATTGCACTACTGTATCAGACAGCTAGCAGTGCAATAGTATTGTCAAAGCATCTGAAAAC	LG14:5442428-5442507	-
cac-mir-301b	GAGGTCAGCTGCTTTGACGATGTTGCACTACTGTACCATCCTAAAGCAGTGCAATAGTATTGTCATAGCATCCGGCCTT	LG9:1604473-1604551	-
cac-mir-301c	AGAGTCAGCTGCTTTGACAATGTTGCACTACTGTACCATCCATTCTAGCAGTGCAATAGTATTGTCATAGCATTTGGCCTC	LG12:14132784-14132864	-
cac-mir-30a	AGTTTGGGGCTGTAAACATCCCCGACTGGAAGCTTTTTATCCACAGAGCTTTCAGTCCGATGTTTGCCGCTGCCGACTGC	Ice_Fa_000685F:92951-93030	-
cac-mir-30bb	ACTCAGTCCATGTAAACATCCTACACTCAGCTGTGAGAGGCTGAGGGAAGGTTGTTTACTTGGACTGGCTGGA	Ice_Fa_000685F:92776-92848	-
cac-mir-30c-1	TTATAGGCAGTGTAAACATCCTACACTCTCGGTGTCTCCCCCTGGTGGCCGGGAGGGGGTATGTTTATTCTGCCTGGCTCAGGAACTCAAC	LG15:7695779-7695869	+
cac-mir-30c-2	TCTCCAGGCATGTAAACATCCTACACTCTCAGCTGTGTTATCATGGAGCTGGGAGAAGGGTGTTTACCCTCCTGGTGATA	LG11:11863439-11863518	+
cac-mir-30e-1	AGGTGCTGTAAACATCCTTGACTGGAAGCTGGGGTTAGACAGCGTGAGCTTTCAGTCGGATGTTTGCAGCATCTTATTG	LG11:11863113-11863191	+
cac-mir-30e-2	GGCTACTGTAAACATCCTTGACTGGAAGCTGGTGTTTGGTTCTTGGGCTTTCAGTCGGATGTTTGCAGCAGCCAACTG	LG15:7691932-7692009	+
cac-mir-31	CCAGGAAGGAAGGCAAGATGTTGGCATAGCTGATGAAGTTAAAACCCTGCTATGCCACCACATTGCCATCTTTCTCCCCCCTCTGCAGCAA	LG18:7892411-7892501	-
cac-mir-338-1a	TGCTTCCTGGAACAATATCCTGGTGCTGCCTGAGTGATCCTCACAGACTCCAGCATCAGTGATTTTGTTGCAGGTGGT	LG19:5395655-5395732	-
cac-mir-338-1b	CTCTCCCTGCAACAACATCCTGGTGCTGCCTGAGTAATTGTCACAAACTCCAGCATCAGTGATTTTGTTGCCGGGGGC	LG8:20402927-20403004	-
cac-mir-338-2	GCCTCCTGGGAACAGCATCCTGATGCTGTCAGGGTGTGTGAAGCAGAACTCCAGCATCAGTGATTTTGTTGCCAGGGGG	LG19:22095349-22095427	+
cac-mir-33a-1	GGTGGCTGTGGTGCATTGTAGTTGCATTGCATGGTCCTGATGACGAGTGCAATGTGTCTGCAGTGCAACACAGAGGCCTC	LG8:27616571-27616650	-
cac-mir-33a-2	CAGAGCTGTGGTGCATTGTAGTTGCATTGCATGTTGGGTAACTCCGTGCAATGGATCTGCTCTGCAACACAGAACT	LG19:22527441-22527516	-
cac-mir-33b	CAGAGCTGTTGTGCATTGTAGTTGCATTGCATGTGTGTCTCAGCGGAGTGCAATGTTCCTGCAGTGCAACACAGAGCTGGC	LG8:18223309-18223389	+
cac-mir-34a	GGTGTTTCTCTGGCAGTGTCTTAGCTGGTTGTTGTGAGGAGTGAAAACGAAGCAATCAGCAAGTATACTGCCGCAGAAACTCTTC	Ice_Fa_000343F:226310-226394	+
cac-mir-3618	AATGCATTGTTGATTTCCAATAATTGAGACAGTGATTCTGAAAGCTGTCTACATTAATGAAAAGAACAATGTAGACAGCT	LG9:41067940-41068019	+

cac-mir-363	TGACATTGCTGCTTGTCTCGCTGTTTTCGGGTGGATGACTTCACAATTTCAATACTCGAAAAAATTAGAAATTGCACAGTATCCATCTGCAAGCCGCCAGA	LG10:4338916-4339016	+
cac-mir-365	GGGAGAGAGAAAAAGGCAGCAAGAAAAGTGAGGACTTTTAAGGGCAGCTGTGTTTTATTAACCCAGTCATAATGCCCCTAAAAATCCTTATTGCTCTTGCA	LG1:24200163-24200263	-
cac-mir-375	GTATTTGCTTCACGTTGAGCCTCACGTACAATACCTGAAGATTAAGTTTTGTTCGTTCGGCTCGCGTTACGCAGGTACG	LG21:12224058-12224136	+
cac-mir-429	TCCTTCCAGCCTGTTGATAGGCGTCTTACCAGACATGGTTAGATGTAATTATTGGTGTCTAATACTGTCTGGTAATGCCGTCCATTAAATG	LG7:13022488-13022578	+
cac-mir-449	ATGCTCATATTGTGGGTGGTTGGTAGGCAGTGTCTTGTTAGCTGGTTTGTTCTTTGCGTGCCAGCTTACCTGCTGCTGCCCTCCAACTTTCCATTCAAACCCAC	LG9:17977903-17978006	+
cac-mir-451	GGGGGCCGTCAAACCGTTACCATTACTGAGTTTCAGTTAGGGTAAGGGTTATACGGCCCTCGC	LG14:11099605-11099667	-
cac-mir-454b	ACGCACCCCTATGTTGCCTCTGCTTTCAATAGAGTCAACAGAGTAGTGCAATATGGCTTAGGGTCTTGACTCTGAGGGGTGGTCTCT	LG9:1599902-1599988	-
cac-mir-455a	GATGTGAGGGTATGTGCCCTTGGACTACATCGTGGAACCCAGCACCATGCAGTCCATGGGCATATACACTTTCCTCAAGG	LG9:14598393-14598472	-
cac-mir-455b	GGCGTGAGGGTATGTGCCCTTGGACTACATCGTGGAAGCCAGCACCATGCAGTCCATGGGCATATACACTTGCCTCATGG	LG12:1228182-1228261	+
cac-mir-456	AGGCAGCTGCTGGCTCTGTGAGCAGGCATCTTCCCAGCCTACATGTAGATCCAGGAATCTGCAGGCTGGTTAGATGGTTGTCACATGTCTG	LG15:1466389-1466479	+
cac-mir-458	TGCAGACGGCAGCGCCATTTTCAGAGCTATCAGTGTGAAAGTGATCATAGCTCTTTAAATGGTACTGCTGTCGGTAT	LG10:37571761-37571837	+
cac-mir-459	CGTGTCGCTGTCAGTAACAAGGATTCATCCTGTTGTGACTCTGCA	LG2:5672106-5672150	-
cac-mir-460-1	CTTTACAGTTCCTGCATTGTACACACTGTGCGTATAAGTGACTTAAGCACAGCGCATACAATGTGGATGCTGTGCTA	LG17:21784965-21785041	-
cac-mir-462	GGTGGGATTGTAACGGAACCCATAATGCAGCTGTAATTCTGAGCTCCAGCTGGTTATAGGCTCCGTTTCCATCCCATGATG	LG7:15851978-15852058	-
cac-mir-489	GGTGGCCTGGTGGTCGTATGTATGACGTCATTTACTTCAGTGTTTGGAGTGACATCATATGTACGGCTGCTAAACTGCTAC	LG11:18260879-18260959	-
cac-mir-499	ACGGAGGCAGTTAAGACTTGCAGTGATGTTTAGGGCAATGATCACATGAACATCACTTTAAGTCTGTGCTGGCTCTCTC	LG5:35637055-35637133	-
cac-mir-551	TGCCTCCACAGATACATTCTGCCTGCCCTTAGAAACCAAGTGTGGGTGTGGCCGGGTTAAACACACAATGGCGACCCATCCTTAGTTTCTGAGGTCCTGCA	LG13:36890714-36890814	-
cac-mir-7132	TTTGCCCCTGGACTTGGTCAAAGCTCCTCAGCAGAATAAGAGACTCTGAGGCGTTTAGAACAAGTGCAGGGTCCTGGA	LG1:25135918-25135995	-
cac-mir-7133	AAATGGCTCAGATGTTGAGTATCAAACTGTATGTTGTATACGTCATATATAGTTTGATACACAGCACAATAAGCCATTTCC	LG7:7476438-7476518	+
cac-mir-7147	TGCCCAGTGCTGTACCATGCTGGTAGCCAGTGTGTGGTCGGCTTTGCTGGTGACCAGTGTTGTGCCTCACTGGCTGCTCAGTGGAGAGCCC	LG20:14488165-14488255	+
cac-mir-722a	GACTGGAACAGAGTGGAATTTGAAACGTTTTAGCCAAAAATGTTTCCATGGTCAAGGTGTTTTTTGCAGAAACGTTTCAGATTTCGTTTTG	LG1:13532886-13532976	+
cac-mir-722b	GACTGGAACAGAGTGGAATTTGAAACGTTTTCGCCAAAAATGTTTCAATGGTCAAGGTGTTTTTTGCAGAAACGTTTCAGATTTCGCTCCG	LG8:19731018-19731108	+
cac-mir-723a	GCTGGTCTATAAAGGCATGGATAAAGGCAGTTTTGATGATGTTACTTCTTTTATTTTTAAGAAGACATCAGATAAATCTGTGCTTATCTTT	Ice_Fa_000509F:337262-337352	+
cac-mir-723b	CCCATATACTACGGCAAGGATAACGGCAGCTTTGTATGATGTTACTTCTGACATATCGGAGAAGACATCAGATAAATCTGTGCTTATCCTT	LG8:7011400-7011490	-
cac-mir-724a	AGACTGGATTTAAAGGGAATTTGCGACTGTTAGTCAAAACATTAGAACAGCCACACCTTCCTTTTAAGATCTTGCCTGCTG	LG5:31878431-31878511	-
cac-mir-724b	AGACTGGGTTTAAAGGGAATTTGCGACTGTTAATCAAACCTTTCGAACAGCCACACCTTCCTTTTAAGATCTTGTCTGCTG	LG7:3006957-3007037	+

cac-mir-725-1	TTATCCGCACAGCTGGAAACTTTGCCTGGGTATTTATGTGCTTAAAATTTCAGTCATTGTTTCTGGTCGTGTGTGTAAG	LG4:27912239-27912317	-
cac-mir-726	TGTTTCGAGGGTAGGTCTGGAATTCCGCTAGTTCTGAACTATTCATGGTTGGTAAAAGTTCTCTACTAGCAGAACTCGGATTTGCTCCCGACTAGCGACGG	LG5:35909231-35909331	-
cac-mir-727a	TTTCAGTCTTCAGTTCCTCCCAGCCCATAACTATGGCAGAAGTGTGTTGAGGCGAGTTGAATACTTAAAGCGCTGCA	LG20:11563739-11563815	+
cac-mir-727b	ACTGAATGTCCTGTATGTCATTTTCAGTCTTCAATTCCTCCCAGCCCGTTACTATGGAAACTGTGAGTTGAGGCGAGTTGAAGACTAAAAATGCTGTACAGATACAAGGAA	LG17:7384846-7384956	+
cac-mir-728a	GCCCTGGGGAAATGTAGTTGACTTAAAGTATACGTGTGGAACCAGAGAGTATACTAAGTACACTACGTTTAT	LG20:11565444-11565515	+
cac-mir-728b	TTTTGCGCTCATGAGAAAATGTAGTAGACTATAAGTATACCTGAACATGTACGTATACTAAGTATACTACGTTTACTCAAGAGTGGCGTCTTC	LG17:7390537-7390629	+
cac-mir-730	TGTTCAGGCCTCCTCATTGTGCATGCTGTGTGTATTTCTGTGAGTACCACACAGCGCCTGCAATGTGGAGGCTGAGACACGCAGAGACGCCTCACACACAC	LG18:14296988-14297088	-
cac-mir-731	GCTTTTCCTCAATGACACGTTTTCTCCCGGATTGCTGAGAACAGGAAACGCAACCGGGAATTTCGTGTCAGCAAAGAATGACA	LG7:15851506-15851588	-
cac-mir-734	CCTCACCGCGCTGCCTTTGATTTCAAGAGCTGAACTATTCTGCAACATTTGTTGATGTGGTCCTGCAAGTAAATGCTGCAGAATTGTGCTCCTCTTGGAAT	LG21:14805856-14805956	+
cac-mir-736	GCAGATCAGCTTTTTGTTTGTGTTATGTTCCTACTAAAATGTAAGACGAACAAAAAGTGTGTCTGTTAGAT	LG17:12203394-12203464	-
cac-mir-737	CTCCTCTGTTGTTTTTTTAGGTTTTGATTTTTGTAAGCGCTAAATGAGAAAATCAAAACCTAAAGAAAGTCCAGCGCTGAC	Ice_Fa_004695F:7602-7682	-
cac-mir-7552a	TCTCTTTTCTTTACAATTAAAGGATATTTCTCGTGAAACATCGACACAGAAATGTATTTTAATTGTTTGGATAAGTTTAGTCCGGCTCACG	Ice_Fa_001027F:25872-25962	+
cac-mir-7552b	AGGCATAATCTTACAATTAAAGGATATTTCTCGTGACTGCAATAAAAACGGAAATATCTCTTAATTGCTTGGTTACGTCTGTCAGAGGCAC	LG15:25894555-25894645	+
cac-mir-7a-1	TTACGCGTTGTGGAAGACTAGTGATTTTGTTGTTGTACATTTTCGTTCATGACAACAAGTCCCAGTCTCCCTCAGCGAGTCACGCCACCAC	LG6:6287604-6287694	+
cac-mir-7a-3	TGGTGCTGAGTGGAAGACTAGTGATTTTGTTGTGATGCTGATCAATGAACAACAAGTCCCAGTCTCCCTCTCAGCACAAGCAGAGCTCTTC	LG17:14995766-14995856	+
cac-mir-7a-4	CATAGCAGCATGGAAGACTAGTGATTTTGTTGTTTTGTGATTAAGCTGACAACAAGTCATTGTCCTCCTCACTGCTT	LG16:24839300-24839376	-
cac-mir-7bb	TGCTGCTGTGTGGAAGACTAGTGATTTTGTTGTTTTTAGTTAGATCAACCAACAACAAATCACAGTCTGCCAAATAGCACGGGCTCCTCCT	LG9:24299842-24299932	-
cac-mir-8159	GCCCCCAGGCTCAGTAACTGGAATCTGTCCCTGCAGAGTGAAAGAGTGCAGGGCCTGGCTGGTTACTGCTCCCAGGGGCCAGGCTACTCTCCAAAGGGGCC	LG14:24804996-24805096	-
cac-mir-9-1	TTGTCTGTTATCTTTGGTTATCTAGCTGTATGAGTGATGCACATTTTTCATAAAGCTAGATAACCGAAAGTAACAAGAATC	Ice_Fa_000667F:118856-118936	-
cac-mir-9-2	TAGGTTGTTATCTTTGGTTATCTAGCTGTATGAGTGTTCTGCTCGTCATAAAGCTAGATAACCGAAAGTAAAAACTACT	LG9:31822425-31822503	+
cac-mir-92a-1	CTTTCTGTGCAGGTTGGGAGAGGAAGCAATGCTCTGTACTTGTGTGGTATTGCACTTGTCCCGGCCTGTTGAGGACATG	LG21:17071919-17071997	+
cac-mir-92a-2	CTTCCTGTGCAGGTGGGGATTTGTAGCAATGCTGAGTACCTGGAGGTATTGCACTTGTCCCGGCCTGTATAGGAACGA	LG3:26486378-26486455	+
cac-mir-93	GCTGGGTGTCAAAAGTGCTGTTTGTGCAGGTAGCAGTCATCCACCTACTGCAAAACCAGCACTTCAGGCACACGGTTC	LG10:28714207-28714284	-
cac-mir-9-3	TTGGTTGTTATCTTTGGTTATCTAGCTGTATGAGTGATGTACATTCTTCATAAAGCTAGATAACCGAAAGTAACAAGAATC	LG16:37207042-37207122	-
cac-mir-9-4	TTAGTTTTTCTCTTTGGTTATCTAGCTGTATGAGTTATGTAATATCATAAAGCTAGAGAACCGAATGTACAAACTAATT	LG4:31266933-31267011	+
cac-mir-9-5	CCTGTTACTATCTTTGGTTATCTAGCTGTATGAGTGTTAAACCTCCATCATAAAGCTAGATAACCGAAAGTAGGAATGACC	Ice_Fa_000223F:455562-455642	+

cac-mir-9-6	TCTGTTTCTGTCTTTGGTTATCTAGCTGTATGAGTTTTAAATACAGCATA AAGCTAGATAACCGAAAGTAGAAACGACT	LG6:15993720-15993798	+
cac-mir-96-1	CTTTGCCCATTTTGGCACTAGCACATTTTTGCTTCTGTATATATACTTTG AGCAATCATGTGTAGTGCCAATATAGGACAAGACAGACTTC	LG23:4351232-4351322	+
cac-mir-96-2	CTTTGCCCATTTTGGCACTAGCACATTTTTGCTTTGTTTCTTTTCGTTTGA GCAATCATGTGTGGTGCCAATATAGGACAAGATAGGCAAT	LG6:18738868-18738958	+
cac-mir-9-7	TTAGTTTGTATCTTTGGTTATCTAGCTGTATGAGTTTTAATTTCATAAAG CTAGAGAACCGAAAGTACGAACTGACG	LG17:15341992-15342068	+
cac-mir-99-2	ATTTGGCACAAACCCGTAGATCCGATCTTGTGGCGAATCTGACAGCACA AGCTCGCCTCTGTGGGTCTTTGTCATCGT	LG14:5184288-5184365	+

Supplementary Table 7. Statistics for the analysis of icefish repetitive elements.

		Repeats	Number	Length occupied	Percentage genome (%)
Transposable element	LTR retrotransposon	LTR	1,512	154,453	0.01
		LTR/Copia	1,423	1,254,557	0.12
		LTR/DIRS	28,935	21,963,956	2.06
		LTR/ERV1	14,347	9,558,534	0.90
		LTR/ERVK	953	147,808	0.01
		LTR/Gypsy	54,611	21,218,816	1.99
		LTR/Ngaro	3,291	1,806,225	0.17
		LTR/Pao	6,780	3,393,081	0.32
		**Total**	**111,852**	**59,497,430**	**5.58**
	non-LTR retrotransposon	SINE	15,410	2,555,151	0.24
		SINE/5S	1,996	186,954	0.02
		SINE/ID	563	47,443	0.00
		SINE/MIR	19,288	3,038,263	0.29
		SINE/tRNA	570	206,591	0.02
		**Total**	**37,827**	**6,034,402**	**0.57**
		LINE	10,421	5,399,187	0.51
		LINE/CR1	3,384	1,119,014	0.11
		LINE/I	8,488	2,563,237	0.24
		LINE/Jockey	5,508	1,365,271	0.13
		LINE/L1	8,227	3,347,285	0.31
		LINE/L2	100,525	40,927,414	3.84
		LINE/Penelope	1,301	284,035	0.03
		LINE/Proto2	2,374	780,797	0.07
		LINE/Rex	72,096	16,705,191	1.57
		LINE/RTE	8,466	4,759,124	0.45
		**Total**	**220,790**	**77,250,555**	**7.25**
	DNA transposon	DNA	116,939	25,573,600	2.40
		DNA/Academ	3,694	896,244	0.08
		DNA/CMC	13,082	3,359,603	0.32
		DNA/Crypton	9,700	2,343,498	0.22
		DNA/Ginger	601	194,239	0.02
		DNA/Harbinger	2,760	1,440,879	0.14
		DNA/hAT	332,737	93,810,547	8.80
		DNA/Kolobok	8,025	2,297,447	0.22
		DNA/Maverick	10,958	3,101,964	0.29
		DNA/Merlin	291	111,556	0.01
		DNA/MULE	250	19,659	0.00
		DNA/Novosib	2,194	641,603	0.06
		DNA/P	4,543	2,087,502	0.20
		DNA/PIF	32,016	12,688,102	1.19
		DNA/PiggyBac	7,135	2,199,829	0.21
		DNA/Sola	484	201,769	0.02
		DNA/TcMar	15,385	7,248,703	0.68
		DNA/Zisupton	889	198,058	0.02
		RC_Helitron	2,519	2,163,670	0.20

	**Total**	**564,202**	**160,578,472**	**15.07**
	**Unclassified**	628,836	201,836,786	18.94
**Total Interspersed**		**1,563,507**	**505,197,645**	**47.41**
	**Small RNA**	**1,074**	**454,551**	**0.04**
	**Satellites**	**5,834**	**2,808,492**	**0.26**
	**Simple repeats**	**324,248**	**26,602,004**	**2.50**
	**Low complexity**	**26,275**	**1,647,308**	**0.15**
**Total**			**536,710,000**	**50.36**

Supplementary Table 8. Genome assemblies/Gene models used in this study.

Common name	Species	Assembly/Gene model
Icefish	*Chaenocephalus aceratus*	This study
Dragonfish	*Parachaenichthys charcoti*	PRJNA330735
Bullhead notothen	*Notothenia coriiceps*	GCA_000735185.1
Stickleback	*Gasterosteus aculeatus*	BROAD S1
Platyfish	*Xiphophorus maculatus*	Xipmac4.4.2
Amazon molly	*Poecilia formosa*	Poecilia_formosa-5.1.2
Medaka	*Oryzias latipes*	HdrR
Tilapia	*Oreochromis niloticus*	Orenil1.0
Takifugu	*Takifugu rubripes*	FUGU 5.0
Tetraodon	*Tetraodon nigroviridis*	TETRAODON 8.0
Cod	*Gadus morhua*	gadMor1
Zebrafish	*Danio rerio*	GRCz10
Cavefish	*Astyanax mexicanus*	AstMex102

Supplementary Table 9. Summary of orthologous gene clusters analyzed in 13 species*.

Species name	No. of coding genes	No. of gene families	No. of genes in gene families	No. of orphan genes	No. of unique gene families	Ave. No. of genes in gene families
Icefish	30,773	18,636	24,159	6,614	581	1.3
Dragonfish	32,713	21,417	26,550	6,163	429	1.2
Bullhead notothen	32,635	20,153	23,387	9,248	34	1.2
Stickleback	20,786	16,131	19,601	1,185	48	1.2
Platyfish	20,379	16,966	19,748	631	6	1.2
Amazon molly	23,615	18,019	22,488	1,127	116	1.2
Medaka	19,699	14,981	18,136	1,563	132	1.2
Tilapia	21,437	16,282	20,541	896	139	1.3
Takifugu	18,523	14,767	17,747	776	47	1.2
Tetraodon	19,602	14,835	17,783	1,819	47	1.2
Cod	20,094	15,292	18,107	1,987	72	1.2
Zebrafish	25,397	16,334	22,815	2,582	320	1.4
Cavefish	23,042	16,014	19,522	3,520	124	1.2

* Species list are Supplementary Table 8.

Supplementary Table 10. Gene Ontology of expanded genes families in the icefish genome relative to the 13 species*.

Category	GO_acc	Term	p-value
P	GO:0043623	cellular protein complex assembly	6.40E-07
P	GO:0050906	detection of stimulus involved in sensory perception	2.00E-06
P	GO:0034622	cellular macromolecular complex assembly	2.20E-05
P	GO:0007606	sensory perception of chemical stimulus	3.00E-05
P	GO:0007600	sensory perception	2.90E-05
P	GO:0006461	protein complex assembly	4.80E-05
P	GO:0065003	macromolecular complex assembly	0.00023
P	GO:0071205	protein localization to juxtaparanode region of axon	0.029
P	GO:0007626	locomotory behavior	0.0092
P	GO:0034728	nucleosome organization	0.04
P	GO:0032507	maintenance of protein location in cell	0.04
P	GO:0042742	defense response to bacterium	0.027
P	GO:0065004	protein-DNA complex assembly	0.013
P	GO:0000050	urea cycle	0.004
P	GO:0019627	urea metabolic process	0.004
P	GO:0071466	cellular response to xenobiotic stimulus	0.0031
P	GO:0002460	adaptive immune response based on somatic recombination of immune receptors built from immunoglobulin superfamily domains	0.028
P	GO:0035113	embryonic appendage morphogenesis	0.044
P	GO:0045185	maintenance of protein location	0.04
P	GO:0006935	chemotaxis	0.024
P	GO:0043604	amide biosynthetic process	0.017
P	GO:0002449	lymphocyte mediated immunity	0.0048
F	GO:0019001	guanyl nucleotide binding	9.30E-07
F	GO:0004984	olfactory receptor activity	1.80E-06
F	GO:0017111	nucleoside-triphosphatase activity	0.001
F	GO:0004053	arginase activity	0.0011
F	GO:0004190	aspartic-type endopeptidase activity	0.0014
F	GO:0070001	aspartic-type peptidase activity	0.0014
F	GO:0016818	hydrolase activity, acting on acid anhydrides, in phosphorus-containing anhydrides	0.0019
F	GO:0016462	pyrophosphatase activity	0.0018
F	GO:0005506	iron ion binding	0.002
F	GO:0005093	Rab GDP-dissociation inhibitor activity	0.004

F	GO:0016813	hydrolase activity, acting on carbon-nitrogen (but not peptide) bonds, in linear amidines	0.0091
F	GO:0008158	hedgehog receptor activity	0.01
F	GO:0004617	phosphoglycerate dehydrogenase activity	0.01
F	GO:0035004	phosphoinositide 3-kinase activity	0.04
F	GO:0004842	ubiquitin-protein ligase activity	0.012
F	GO:0003964	RNA-directed DNA polymerase activity	0.014
F	GO:0003796	lysozyme activity	0.017
F	GO:0020037	heme binding	0.027
F	GO:0003779	actin binding	0.033
F	GO:0019787	small conjugating protein ligase activity	0.022
F	GO:0032549	ribonucleoside binding	0.029
F	GO:0016881	acid-amino acid ligase activity	0.025
F	GO:0005540	hyaluronic acid binding	0.016
F	GO:0016303	1-phosphatidylinositol-3-kinase activity	0.04
F	GO:0004601	peroxidase activity	0.047
C	GO:0034702	ion channel complex	0.029
C	GO:0034703	cation channel complex	0.012
C	GO:0005581	collagen	0.014
C	GO:0043232	intracellular non-membrane-bounded organelle	0.01
C	GO:0005577	fibrinogen complex	0.0091
C	GO:0005604	basement membrane	0.024

* Species list are Supplementary Table 8.

Abbreviations: C, Cellular component; F, Molecular function; P, Biological process

Supplementary Table 11. Gene Ontology for contracted gene families in the icefish genome relative to the 13 species*.

Category	GO acc	Term	p-value
P	GO:0046835	carbohydrate phosphorylation	1.90E-13
P	GO:0006003	fructose 2,6-bisphosphate metabolic process	1.30E-12
P	GO:0006000	fructose metabolic process	6.70E-10
P	GO:0044237	cellular metabolic process	4.60E-08
P	GO:0010467	gene expression	1.30E-07
P	GO:0006004	fucose metabolic process	4.00E-07
P	GO:0005996	monosaccharide metabolic process	5.20E-07
P	GO:0044260	cellular macromolecule metabolic process	5.20E-07
P	GO:0006066	alcohol metabolic process	1.40E-06
P	GO:0019318	hexose metabolic process	1.50E-06
P	GO:0044238	primary metabolic process	3.90E-06
P	GO:0031145	anaphase-promoting complex-dependent proteasomal ubiquitin-dependent protein catabolic process	4.20E-06
P	GO:0007200	activation of phospholipase C activity by G-protein coupled receptor protein signaling pathway coupled to IP3 second messenger	4.90E-06
P	GO:0007202	activation of phospholipase C activity	7.60E-06
P	GO:0010863	positive regulation of phospholipase C activity	9.40E-06
P	GO:0007067	mitosis	9.00E-06
P	GO:0022613	ribonucleoprotein complex biogenesis	9.60E-06
P	GO:0000280	nuclear division	1.10E-05
P	GO:0010518	positive regulation of phospholipase activity	1.40E-05
P	GO:0010517	regulation of phospholipase activity	1.40E-05
P	GO:0048285	organelle fission	1.70E-05
P	GO:0030071	regulation of mitotic metaphase/anaphase transition	2.10E-05
P	GO:0043170	macromolecule metabolic process	2.20E-05
P	GO:0007091	mitotic metaphase/anaphase transition	2.10E-05
P	GO:0060193	positive regulation of lipase activity	2.50E-05
P	GO:0007178	transmembrane receptor protein serine/threonine kinase signaling pathway	2.90E-05
P	GO:0060191	regulation of lipase activity	3.00E-05
P	GO:0000087	M phase of mitotic cell cycle	3.80E-05
P	GO:0008152	metabolic process	3.90E-05
P	GO:0042254	ribosome biogenesis	3.90E-05
P	GO:0016070	RNA metabolic process	4.20E-05
P	GO:0090092	regulation of transmembrane receptor protein serine/threonine kinase signaling pathway	4.30E-05
P	GO:0048015	phosphoinositide-mediated signaling	4.30E-05
P	GO:0006607	NLS-bearing substrate import into nucleus	7.90E-05
P	GO:0045333	cellular respiration	9.30E-05
P	GO:0007176	regulation of epidermal growth factor receptor activity	0.00011
P	GO:0007175	negative regulation of epidermal growth factor receptor activity	0.00011
P	GO:0018345	protein palmitoylation	0.00011
P	GO:0010469	regulation of receptor activity	0.00011
P	GO:0009987	cellular process	0.00011
P	GO:0006457	protein folding	0.00012
P	GO:0006396	RNA processing	0.00012
P	GO:0006364	rRNA processing	0.00014
P	GO:0055014	atrial cardiac muscle cell development	0.00014
F	GO:0003873	6-phosphofructo-2-kinase activity	3.80E-13

F	GO:0019200	carbohydrate kinase activity	8.80E-13
F	GO:0008443	phosphofructokinase activity	3.00E-11
F	GO:0003746	translation elongation factor activity	1.40E-05
F	GO:0031386	protein tag	2.70E-05
F	GO:0017124	SH3 domain binding	4.80E-05
F	GO:0051219	phosphoprotein binding	0.00011
F	GO:0001784	phosphotyrosine binding	0.00011
F	GO:0045309	protein phosphorylated amino acid binding	0.00011
C	GO:0030684	preribosome	2.20E-10
C	GO:0030529	ribonucleoprotein complex	1.70E-09
C	GO:0043231	intracellular membrane-bounded organelle	1.60E-08
C	GO:0043227	membrane-bounded organelle	1.80E-08
C	GO:0044424	intracellular part	2.90E-08
C	GO:0005634	nucleus	3.70E-08
C	GO:0005622	intracellular	3.40E-07
C	GO:0043229	intracellular organelle	7.80E-07
C	GO:0043226	organelle	9.10E-07
C	GO:0034457	Mpp10 complex	1.10E-06
C	GO:0005737	cytoplasm	2.60E-06
C	GO:0030686	90S preribosome	2.40E-06
C	GO:0045121	membrane raft	6.70E-06
C	GO:0030672	synaptic vesicle membrane	1.40E-05
C	GO:0032040	small-subunit processome	2.00E-05
C	GO:0030687	preribosome, large subunit precursor	2.10E-05
C	GO:0044428	nuclear part	5.80E-05
C	GO:0032991	macromolecular complex	6.10E-05
C	GO:0044455	mitochondrial membrane part	6.70E-05
C	GO:0030665	clathrin coated vesicle membrane	9.80E-05
C	GO:0016469	proton-transporting two-sector ATPase complex	0.0001
C	GO:0005689	U12-type spliceosomal complex	0.00013
C	GO:0044452	nucleolar part	0.00018
C	GO:0005680	anaphase-promoting complex	0.00018
C	GO:0005687	U4 snRNP	0.00022
C	GO:0031410	cytoplasmic vesicle	0.00029
C	GO:0031307	integral to mitochondrial outer membrane	0.0003
C	GO:0044444	cytoplasmic part	0.00028
C	GO:0000152	nuclear ubiquitin ligase complex	0.0003
C	GO:0005686	U2 snRNP	0.00041
C	GO:0071564	npBAF complex	0.00041
C	GO:0031982	vesicle	0.00039
C	GO:0016507	fatty acid beta-oxidation multienzyme complex	0.00041
C	GO:0000243	commitment complex	0.00042
C	GO:0030532	small nuclear ribonucleoprotein complex	0.00055
C	GO:0071011	precatalytic spliceosome	0.00058
C	GO:0000276	mitochondrial proton-transporting ATP synthase complex, coupling factor F(o)	0.00072
C	GO:0031306	intrinsic to mitochondrial outer membrane	0.0007
C	GO:0044446	intracellular organelle part	0.0009
C	GO:0044422	organelle part	0.00087
C	GO:0030688	preribosome, small subunit precursor	0.00089

* Species list are Supplementary Table 8.

Abbreviations: C, Cellular component; F, Molecular function; P, Biological process

Supplementary Table 12. CAFE[5] gene family analysis results.

Species	No. of coding genes	No. of Expanded families	No. of Contracted families.	No. of Genes lost
Icefish	30,773	1793 (280)	6831 (6)	6869
Dragonfish	32,713	1174 (162)	4057 (1)	4080
Bullhead notothen	32,661	1178 (8)	5534 (2)	5640
Stickleback	27,576	465 (59)	10704 (2)	10732
Platyfish	20,779	184 (3)	1928 (32)	2002
Amazon molly	24354	1195 (165)	780 (1)	781
Medaka	20459	452 (78)	4953 (3)	5018
Tilapia	27088	800 (133)	4047 (0)	4057
Takifugu	18,523	390 (47)	1924 (3)	1935
Tetraodon	23,118	530 (24)	1895 (7)	1927
Cod	22,100	391 (21)	14427 (3)	14523
Zebrafish	25,403	1183 (78)	2205 (0)	2241
Cavefish	23698	775 (8)	2542 (16)	2753

Supplementary Table 13. CAFE[5] results for significantly expanded genes in icefish.

Category	GO acc	Term	*p*-value
F	GO:0005488	binding	2.94E-03
F	GO:0005200	structural constituent of cytoskeleton	5.48E-03
F	GO:0030246	carbohydrate binding	5.98E-03
F	GO:0005506	iron ion binding	1.07E-02
F	GO:0003924	GTPase activity	2.12E-02
F	GO:0008199	ferric iron binding	1.79E-02
F	GO:0016702	oxidoreductase activity, acting on single donors with incorporation of molecular oxygen, incorporation of two atoms of oxygen	3.04E-02
F	GO:0016701	oxidoreductase activity, acting on single donors with incorporation of molecular oxygen	3.04E-02
F	GO:0004984	olfactory receptor activity	4.29E-02
P	GO:0051258	protein polymerization	1.42E-02
P	GO:0043623	cellular protein complex assembly	1.86E-02
P	GO:0007017	microtubule-based process	2.30E-02
P	GO:0034622	cellular macromolecular complex assembly	3.31E-02
P	GO:0006461	protein complex assembly	4.28E-02
P	GO:0070271	protein complex biogenesis	4.34E-02
P	GO:0006826	iron ion transport	2.04E-02
P	GO:0000041	transition metal ion transport	2.54E-02
P	GO:0006879	cellular iron ion homeostasis	2.79E-02
P	GO:0046916	cellular transition metal ion homeostasis	2.79E-02
P	GO:0055072	iron ion homeostasis	3.29E-02
P	GO:0055076	transition metal ion homeostasis	3.54E-02
P	GO:0050911	detection of chemical stimulus involved in sensory perception of smell	4.29E-02
P	GO:0050907	detection of chemical stimulus involved in sensory perception	4.53E-02
P	GO:0050906	detection of stimulus involved in sensory perception	4.53E-02
P	GO:0006875	cellular metal ion homeostasis	4.78E-02
P	GO:0009593	detection of chemical stimulus	4.78E-02
C	GO:0005874	microtubule	1.00E-02
C	GO:0015630	microtubule cytoskeleton	2.79E-02

Abbreviations: C, Cellular component; F, Molecular function; P, Biological process

Supplementary Table 14. Positively selected genes in the icefish genome. (Likelihood ratio test: α=0.05).

Gene ID	Descriptions
Cac000489	synaptosomal-associated protein 47
Cac000662	septin-10-like isoform X2
Cac001617	ester hydrolase C11orf54 homolog
Cac001771	T-complex protein 1 subunit alpha
Cac002279	delta-type opioid receptor-like
Cac003235	protein FAM3C
Cac003886	tripeptidyl-peptidase 1
Cac004140	poly(ADP-ribose) glycohydrolase ARH3
Cac004885	nuclear pore complex protein Nup205
Cac004994	thioredoxin domain-containing protein 5
Cac005124	integrin alpha-6
Cac005514	high-affinity choline transporter 1-like
Cac006594	phosphatidylinositol 4-kinase type 2-alpha
Cac011177	neutrophil cytosol factor 1
Cac011177	neutrophil cytosol factor 1
Cac012351	lengsin
Cac013420	cerebral cavernous malformations 2 protein isoform X2
Cac013753	sonic hedgehog protein
Cac016183	translation initiation factor eIF-2B subunit beta
Cac016183	translation initiation factor eIF-2B subunit beta
Cac019931	pyruvate dehyrogenase phosphatase catalytic subunit 1
Cac019931	pyruvate dehyrogenase phosphatase catalytic subunit 1
Cac020370	zinc finger MYND domain-containing protein 10
Cac020655	beta-1:4 N-acetylgalactosaminyl transferase 1
Cac020945	mitotic checkpoint protein BUB3
Cac020991	F-box only protein 11 isoform X4
Cac021124	NADPH--cytochrome P450 reductase isoform X1
Cac021496	PX domain-containing protein 1
Cac021612	translocon-associated protein subunit alpha isoform X2
Cac022143	reticulon-4-interacting protein 1: mitochondrial isoform X1
Cac023068	UNC93-like protein MFSD11
Cac023070	mpv17-like protein
Cac025005	protein CYR61
Cac025225	rod cGMP-specific 3',5'-cyclic phosphodiesterase subunit beta
Cac025247	RNA 3'-terminal phosphate cyclase-like protein
Cac026046	AP-1 complex subunit gamma-1 isoform X2
Cac026882	sorting nexin-12
Cac027018	merlin-like
Cac027509	peripheral myelin protein 22-like
Cac027988	transcription cofactor vestigial-like protein 2 isoform X1
Cac028923	glomulin-like
Cac029538	FAST kinase domain-containing protein 1
Cac029831	general transcription factor 3C polypeptide 4

Supplementary Table 15. Gene ontology for positively selected genes in the icefish genome.

Category	GO accessory	Term	p-value
F	GO:0035091	phosphatidylinositol binding	7.25E-05
F	GO:0005543	phospholipid binding	2.48E-04
F	GO:0008289	lipid binding	2.05E-03
F	GO:0016651	oxidoreductase activity, acting on NAD(P)H	8.93E-04
F	GO:0003958	NADPH-hemoprotein reductase activity	3.26E-03
F	GO:0016175	superoxide-generating NADPH oxidase activity	3.26E-03
F	GO:0050664	oxidoreductase activity, acting on NAD(P)H, oxygen as acceptor	3.26E-03
F	GO:0016653	oxidoreductase activity, acting on NAD(P)H, heme protein as acceptor	6.50E-03
F	GO:0010181	FMN binding	1.30E-02
F	GO:0004722	protein serine/threonine phosphatase activity	3.21E-02
F	GO:0004114	3',5'-cyclic-nucleotide phosphodiesterase activity	3.53E-02
F	GO:0004112	cyclic-nucleotide phosphodiesterase activity	3.53E-02
P	GO:0030908	protein splicing	6.50E-03
P	GO:0016539	intein-mediated protein splicing	6.50E-03
P	GO:0016485	protein processing	3.53E-02
P	GO:0051604	protein maturation	3.53E-02
C	GO:0000151	ubiquitin ligase complex	3.53E-02

Abbreviations: C, Cellular component; F, Molecular function; P, Biological process

Supplementary Table 16. Comparison of zona pellucida (*zp*) gene family members in icefish and other Antarctic fish.

	ZPAX1	ZPAX2	ZPC1	ZPC2	ZPC3	ZPC4	ZPC5	ZPB	ZPD	CGL	CGH	**Total**
C. aceratus	29	7	22	13	7	2	33	2	1	8	7	**131**
**N. coriiceps*	1	2	2	1	1	2	1	3	1	4	0	**18**
**P. charcoti*	4	1	2	2	2	1	3	6	1	7	1	**30**
***D. mawsoni*	1	1	1	1	1	1	8	1	1	1	1	**18**
***C. hamatus*	1	4	4	2	0	2	4	1	1	0	0	**19**
***G. acuticeps*	3	1	3	2	2	2	4	2	1	0	0	**20**
***T. bernacchii*	1	1	2	1	1	1	2	1	1	0	0	**11**
***N. angustata*	0	0	1	0	0	0	1	0	0	0	0	**2**
***D.eleginoides*	1	0	1	1	0	0	5	0	0	0	0	**8**
***L. nudifrons*	1	1	1	1	1	1	1	1	1	0	0	**9**
***E.maclovinus*	1	1	2	1	1	1	3	1	0	0	0	**11**

* The number of *zp* genes was annotated from our previous research[6,7].

** The number of *zp* genes of 8 species of Antarctic fish was derived from the Supplementary Table 1 in Cao et al., 2016[8].

Supplementary Table 17. **Genomic distribution of icefish zona pellucida (*zp*) family genes**. The total number of annotated *zp* genes on each contig is indicated. Of the 131 icefish *zp* genes, 109 are distributed on 20 different scaffolds that have more than two genes, and 22 scaffolds have a single *zp* gene.

Contig	ZPAX1	ZPAX2	ZPC1	ZPC2	ZPC3	ZPC4	ZPC5	ZP3	ZP4	ZPB	ZPD	Total
Ice_000281					2		18					20
Ice_010079			7	3								10
Ice_000114					5		5					10
Ice_030120	7	3										10
Ice_040384	3	1					4					8
Ice_051004			4	4								8
Ice_060311	1	1	4									6
Ice_070981			5									5
Ice_080050								1	3			4
Ice_090470			2	2								4
Ice_100683							4					4
Ice_110055								3				3
Ice_120371	3										1	4
Ice_130009						1				1		2
Ice_140019						1				1		2
Ice_150293	2											2
Ice_160991	2											2
Ice_171379	1	1										2
Ice_182290	2											2
Ice_192443	2											2
Ice_200015									1			1
Ice_210036									1			1
Ice_220080								1				1
Ice_230090	1											1
Ice_240126									1			1
Ice_250182				1								1
Ice_260204							1					1
Ice_270271		1										1
Ice_280276				1								1
Ice_290336								1				1
Ice_300443	1											1
Ice_310473								1				1
Ice_320486								1				1
Ice_330519	1											1
Ice_340709							1					1
Ice_350845									1			1
Ice_361246	1											1
Ice_371821				1								1
Ice_382268	1											1
Ice_392717				1								1
Ice_402947	1											1
**Total**	**29**	**7**	**22**	**13**	**7**	**2**	**33**	**8**	**7**	**2**	**1**	**131**

Supplementary Table 18. Annotation of antioxidant defense system related genes in five teleosts. Genes were selected from the gene set of antioxidant activity (M15021) of Gene Set Enrichment Analysis[9] (GSEA, http://software.broadinstitute.org/gsea/index.jsp).

	Icefish	Dragonfish	Antarctic bullhead	Takifugu	Medaka	Platyfish	Zebrafish
Superoxide dismutase	5	3	3	3	3	3	4
Catalase	1	1	1	1	1	1	1
NAD(P)H:quinone acceptor oxidoreductase	33	2	2	2	3	3	1
Glutathione peroxidase	4	7	4	9	6	6	9
Selenoprotein S	1	1	1	1	0	1	1
Eosinophil peroxidase	7	4	4	3	6	4	2
Microsomal glutathione S-transferase 3	2	2	2	2	2	2	2
Cytoglobin	2	2	2	2	2	2	2
8-oxoguanine DNA glycosylase	2	1	1	1	1	1	1
Thioredoxin reductase	3	3	2	2	2	3	2
Peroxiredoxin	6	6	6	6	6	6	6
Apolipoprotein A-IV	4	2	3	6	2	1	4
Total	70	34	31	38	34	33	35

Supplementary Table 19. ***sod* genes identified in teleost genomes**. The Genbank accession number is indicated for each *sod* gene.

	SOD1	SOD2	SOD3
Icefish	CacV3p_001772	CacV3p_016306	CacV3p_021817-1<br>CacV3p_021817-2<br>CacV3p_021817-3
Antarctic Bullhead	XP_010771234.1	XP_010770367.1	XP_010775098.1
Dragonfish	DFtranscrip-31740	DFtranscrip-29679	DFtranscrip-21872
Stickleback	ENSGACG00000020581	ENSGACG00000009000	ENSGACG00000017840
Takifugu	XP_003971372	XP_011610563	XP_011610687
Platyfish	XP_005807113	XP_005802672	XP_005807205
Amazon molly	XP_007555641	XP_007546844	XP_007551838
Medaka	XP_004076261	XP_004083519	XP_004084336
Zebrafish	NP_571369.1	NP_956270	NP_001092706 (chr7)*<br>XP_001332758 (chr1)

* Zebrafish has two *sod3* genes (*sod3a* and *sod3b*); *sod3a* is located on chromosome 7 with similar synteny to that of other teleosts, whereas *sod3b* is located on chromosome 1.

Supplementary Table 20. **Circadian rhythm-related genes identified in teleost genomes**. The location of each gene in each chromosome or scaffold is noted for each gene. Chromosomal location was based on the updated Supplementary Material 13 of Toloza-Villalobos et al. (2015)[10]. Updated information is marked with an asterisk (*). A dash means that the gene was not found on a defined chromosome or scaffold.

	Icefish	Stickleback	Takifugu	Medaka	Platyfish	Zebrafish
per1a	-	-	-	-	-	Chr10:23010981-23028617
per1b	Cacp_005339 Ice_000086:2255858..2264187	groupIII:1517985-1529008	Scaffold_63:506313-512694	Chr18:15189752-15199550	JH556673.1:326107-343686*	Chr7:52277645-52301471
per2a	-	groupI:1855721-1863275*	Scaffold_344:165475-173866	Chr13:31788175-31800229	JH556784.1:457717-480890	-
per2b	Cacp_024627 Ice_000019:2927559..2939828	groupVII:3993749-4000926*	Scaffold_123:243442-251642	Chr17:25658720-25680938	JH556667.1:3479242-3504286	Chr2:48423772-48489514
per3	-	-	Chr19:11805147-11815446*	Chr5:29288111-29298254	JH556773.1:1356945-1376982	Chr11:41435220-41490992
cry1aa	Cacp_026503 Ice_000025:3445991..3456768	groupIV:32004462-32008670	Scaffold_4395:1-440	Chr23:19786254 - 19805276	JH556703.1:1996368-2009995	Chr4:12011692-12027887*
cry1ab	-	groupXIX:17896031-17900731:1*	Scaffold_21:12588914-1264794	Chr6:30902546-30911031	Chr2:17084212-17094297*	Chr18:15104200-15132014*
cry1ba	Cacp_006784 Ice_000125:1848029..1834983	groupXII:13140358-13147524*	Scaffold_278:81132-99203*	Chr7:28299032 - 28474995*	Chr1:29806902 - 29840394*	Chr8:21222300-21255421*
cry1bb	-	-	-	-	-	Chr22:783514-811211*
cry2	-	groupXIX:1002682-1016637	Scaffold_591:4558-10348*	Chr6:22492682-22503706	Chr2: 4478878-4495387*	Chr25:13845092-13873484
clocka	Cacp_021314 Ice_000042:1091634..1109722	-	Scaffold_13:1587298-1594020	-	JH557215.1:159758-185684	Chr20:22202472-22246469

clockb	Cacp_021654 Ice_000015:997555..1012718	groupIX:489361-499374	Scaffold_563:28933-37732	Chr1:14996520-15007939	JH556907.1:802434-820177	Chr1:18174899-18208348
npas2a	Cacp_011192 Ice_000386:348502..366485	groupVII:17209951-17217908	Scaffold_6:1321121-1331856	Chr14:17135493-17147943	-	Chr5:24572014-24614052
arntla	Cacp_017407 Ice_000301:749264..791055	groupXIX:1196030-1206038	Scaffold_30:314818-322668	Scaffold212:822889-852119	JH556669.1:1164407-1191899	Chr25:18381074-18406100
arntlb	-	-	-	-	-	Chr7:67945182-68002908
arntl2a	Cacp_009803 Ice_000337:138232..147037	-	Scaffold_105:333609-341010	Chr6:16473669-16484384	JH556735.1:1789641-1801148	Chr18:15249987-15289314
arntl2b	Cacp_026454 Ice_000025:673361..679455	groupIV:22530990-22537106	Scaffold_657:8465-12073	Chr23:2931241-2954154	JH556873.1:690137-707607	-
csnk1ea	Cacp_012338 Ice_001973:12374..33989	groupV:5628628-5633878	Scaffold_3:2837555-2840657	Chr19:803568-811280	JH559539.1:29-8132	Chr12:20401738-20414656
csnk1eb	Cacp_029385 Ice_000244:45..10972	groupXI:4260952-4268829	Scaffold_253:334382-342511	Chr8:19677134-19684994	JH556752.1:1560221-1567996	-
csnk1da	Cacp_014005 Ice_000322F:247084..274355	Scaffold54:477509-486247	Scaffold_41:895893-902348	Chr8:25232642-25239805	JH557172.1:17306-26673	-
csnk1db	Cacp_006446 Ice_000123F:682442..704673	groupXI:1303431-1312998	-	-	-	Chr12:34815412-34831695
csnk1dc	-	-	-	-	-	Chr3:36172321-36209462
timeless	Cacp_000716 Ice_000119F:944982..953940	Scaffold27:1143834-1158386	Scaffold_47:1354118-1362657	Chr5:27745117-27758015	JH556773.1: 50419-64006	Chr11:3195071-3229802

Supplementary Table 21. **The number of olfactory receptor genes in 6 teleost species.**

	Icefish	Medaka	Stickleback	Takifugu	Tetraodon	Zebrafish
alpha	0	0	0	0	0	0
beta	1	3	1	1	0	7
gamma	2	1	3	0	0	1
delta	161	44	112	55	14	69
epsilon	2	4	4	3	2	13
zeta	10	12	23	10	2	41
eta	35	30	12	31	12	45
theta	0	1	1	1	1	1
theta-2	0	0	0	0	0	1
kappa	1	1	3	1	1	2
lambda	0	1	1	0	0	1
**Total**	**212**	**97**	**160**	**102**	**32**	**181**

References

1. Chalopin, D., Naville, M., Plard, F., Galiana, D. & Volff, J.-N. Comparative analysis of transposable elements highlights mobilome diversity and evolution in vertebrates. *Genome Biol. Evol.* **7**, 567-580 (2015).
2. Niimura, Y. On the origin and evolution of vertebrate olfactory receptor genes: comparative genome analysis among 23 chordate species. *Genome Biol. Evol.* **1**, 34-44 (2009).
3. Park, H., Lee, S. H., Kim, M., Kim, K. H. & Lim, H. S. Polychlorinated biphenyl congeners in soils and lichens from King George Island, South Shetland Islands, Antarctica. Ant. Sci. 22, 31-38 (2010)
4. Opazo, J. C., Butts, G. T., Nery, M. F., Storz, J. F. & Hoffmann, F. G. Whole-genome duplication and the functional diversification of teleost fish hemoglobins. *Mol. Biol. Evol.* **30**, 140-153 (2013).
5. Han, M. V., Thomas, G. W., Lugo-Martinez, J. & Hahn, M. W. Estimating gene gain and loss rates in the presence of error in genome assembly and annotation using CAFE 3. *Mol. Biol. Evol.* **30**, 1987-1997 (2013).
6. Shin, S. C. *et al.* The genome sequence of the Antarctic bullhead notothen reveals evolutionary adaptations to a cold environment. *Genome Biol.* **15**, 468 (2014).
7. Ahn, D.-H. *et al.* Draft genome of the Antarctic dragonfish, Parachaenichthys charcoti. *GigaScience* **6**, 1-6 (2017).
8. Cao, L. *et al.* Neofunctionalization of zona pellucida proteins enhances freeze-prevention in the eggs of Antarctic notothenioids. *Nat. Commun.* **7**, 12987 (2016).
9. Subramanian, A. *et al.* Gene set enrichment analysis: A knowledge-based approach for interpreting genome-wide expression profiles. *Proc. Natl. Acad. Sci. U.S.A.* **102**, 15545-15550 (2005).
10. Toloza-Villalobos, J., Arroyo, J. I. & Opazo, J. C. The Circadian Clock of Teleost Fish: A Comparative Analysis Reveals Distinct Fates for Duplicated Genes. *J. Mol. Evol.* **80**, 57-64 (2015).
